# Catalpol Attenuates Pulmonary Fibrosis by Inhibiting Ang II/AT_1_ and TGF-β/Smad-Mediated Epithelial Mesenchymal Transition

**DOI:** 10.3389/fmed.2022.878601

**Published:** 2022-05-24

**Authors:** Qun Yu, Dewei Zhu, Yang Zou, Kai Wang, Peili Rao, Yunhui Shen

**Affiliations:** ^1^School of Pharmacy, Shanghai University of Traditional Chinese Medicine, Shanghai, China; ^2^Experiment Center for Science and Technology, Shanghai University of Traditional Chinese Medicine, Shanghai, China

**Keywords:** idiopathic pulmonary fibrosis, Ang II, TGF-β/Smad signaling pathway, EMT, catalpol

## Abstract

**Background:**

Idiopathic pulmonary fibrosis (IPF) is a progressive and devastating chronic lung condition affecting over 3 million people worldwide with a high mortality rate and there are no effective drugs. Angiotensin II (Ang II), as a major effector peptide of the renin angiotensin aldosterone system, has been shown to act in tandem with the transforming growth factor-β (TGF-β) signaling pathway to promote the infiltration of inflammatory cells, production of reactive oxygen species (ROS) and profibrotic factors after lung injury, and to participate in the process of epithelial mesenchymal transition (EMT). Catalpol (CAT) has been shown to have anti-inflammatory and antifibrotic effects. However, the effects and mechanisms of CAT on pulmonary fibrosis are not clear.

**Purpose:**

To assess the effects and mechanisms of catalpol on bleomycin-induced pulmonary fibrosis in mice.

**Methods:**

We used bleomycin-induced mouse model of pulmonary fibrosis to evaluate the alleviation effect of CAT at 7, 14, 28d, respectively. Next, enzyme-linked immunosorbent assay, hematoxylin-eosin staining, immunofluorescence, Masson trichrome staining and western blotting were used to study the underlying mechanism of CAT on bleomycin-induced pulmonary fibrosis.

**Results:**

It's demonstrated that CAT exerted a potent anti-fibrotic function in BLM-induced mice pulmonary fibrosis via alleviating inflammatory, ameliorating collagen deposition, reducing the level of Ang II and HYP and alleviating the degree of EMT. Moreover, CAT attenuate BLM-induced fibrosis by targeting Ang II/AT_1_ and TGF-β/Smad signaling *in vivo*.

**Conclusion:**

CAT may serve as a novel therapeutic candidate for the simultaneous blockade of Ang II and TGF-β pathway to attenuate pulmonary fibrosis.

## Highlights

- Catalpol significantly ameliorates bleomycin-induced pulmonary fibrosis (PF) in mice.
- Catalpol attenuates pulmonary fibrosis via TGF-β/Smads signaling pathway.
- Catalpol inhibits bleomycin-induced epithelial mesenchymal transition.
- Catalpol decreases the expression of angiotensin II/type 1 receptor (AT_1_) in PF mice.

## Introduction

Pulmonary Fibrosis (PF) is a chronic interstitial lung disease characterized by massive proliferation of fibroblasts and accumulation of extracellular matrix (ECM). Pulmonary Fibrosis is accompanied by inflammatory injury, the epithelial-mesenchymal transition (EMT) and destruction of tissue structure (1). Pulmonary Fibrosis will cause lung function damage, scar of lung tissue obstructing the flow of oxygen from lungs into the bloodstream for distribution to other organs and patients will have dry cough, dyspnea and other symptoms. As the condition continues to deteriorate, the integrity of lung tissue structure is destroyed, respiratory function can be further affected, seriously endangering the patient's quality of life and even becoming life-threatening (2). Toxic insults, autoimmune injuries, drug-induced injuries, infectious injuries, traumatic injuries or other unknown factors may cause pulmonary fibrosis. Among them, idiopathic pulmonary fibrosis (IPF) is the most common and prevalent type of pulmonary fibrosis and is associated with risk factors such as genetic alterations, viral infections, lifestyle habits, environmental influences, occupational hazards (3). IPF prevalence has a correlation with age and sex. And the elderly is more prone to IPF and males have a higher incidence rate than females (4, 5). Pulmonary fibrosis is also a sequela in severe patients with the Coronavirus Disease 2019 (COVID-19) and severe acute respiratory syndrome (SARS).

The latest ATS/ERS/JRS/ALAT clinical practice guidelines recommend the conditional use of nintedanib and pirfenidone. In addition, glucocorticoids, immunosuppressive agents, and antioxidants are also used clinically in combination with western medicines to treat pulmonary fibrosis (6–9). The application of angiotensin inhibitor combined with glucocorticoid to treat pulmonary fibrosis also has been reported by many clinicians (10). However, due to the inability of existing drugs in reversing the course of pulmonary fibrosis and the lack of lung organ donors, as well as high treatment costs and so on, the median survival of patients with IPF after diagnosis is only between 3 and 5 years (11, 12). Therefore, the development of affordable and effective anti-fibrotic drugs is urgently needed.

Catalpol (CAT), an iridoid compound, exhibits anti-cancer, neuroprotective, anti-inflammatory, diuretic, hypoglycemic, anti-hepatitis virus effects and anti-fibrosis effect in multiple organs. It has been shown that CAT may inhibit bleomycin-induced pulmonary fibrosis in rats through downregulation of Wnt3a and GSK-3β, β-catenin and phosphorylation of Smad3 (13). CAT has become very popular for clinical medication candidate because of its good safety, fewer toxic side effects and convenient administration. In this context, the research and development of CAT have gradually become hot topics of interest. However, the mechanism by which CAT acts in the treatment of pulmonary fibrosis has not been thoroughly investigated. In this study, a mouse pulmonary fibrosis model was established to further explore the mechanism of action of CAT in the treatment of pulmonary fibrosis, in order to provide a reference basis for the pharmacological research and clinical application of this drug.

## Materials and Methods

### Reagents

Catalpol (CAT, CAS: 2415-24-9, MW: 362.331, purity: 98%, dissolved in normal saline (NS) before use, Chengdu Purifa Technology Co., Ltd., Lot No.: PRF21111722,); Bleomycin for injection (chemical for lung-fibrosis modeling, BLM, Harbin Laibotong Pharmaceutical Co., Ltd., Lot No.: 20120727); Pirfenidone (positive control drug, PFD, Beijing Contini Pharmaceutical Co., Ltd., Lot No.: 20210511); Telmisartan (positive control drug, TEL, Shanghai Xinyi Tianping Pharmaceutical Co., Ltd. Lot No.: 87210302); Haematoxylin and eosin (H&E) (719033, Zhuhai Baso Biotechnology Co., Ltd., China); Masson Staining Kits (C210801, Zhuhai Baso Biotechnology Co., Ltd., China); HYP Kit (Nanjing Jiancheng Bioengineering Institute, Nanjing, China); Ang II Kit (Nanjing Jiancheng Bioengineering Institute, Nanjing, China); RIPA lysis buffer (Beyotime, China); Phosphorylated proteinase inhibitor (Beyotime, China); Phenyl methyl sulfonyl fluoride (PMSF, Beyotime, China); TGF-β1 (ab179695, abcom, USA); Anti-Phospho-Smad2 (18338T, CST, USA); Anti-Phospho-Smad3 (9520T, CST, USA); Anti-Smad2/3 (8685S, CST, USA); Anti-Snail (3879T, CST, USA); Anti-Angiotensin II Type 1 Receptor (ab124734, abcom, United States); Anti-MMP2(ab92536, abcom, USA); Anti-MMP9 (ab283575, abcom, USA); Anti-β-actin (AF7018, Affinity, USA); Horseradish peroxidase (HRP)-linked anti-mouse (A0286, Beyotime, China); Anti-rabbit (A0208, Beyotime, China); α-SMA (19245T, CST, USA); E-cadherin antibody (3195T, CST, USA), N-cadherin antibody (13116T, CST, USA); Goat anti rabbit IgG secondary antibody (A0468/A0423; Beyotime; Biotechnology Co. Ltd., Shanghai, China) and 4', 6-diamidino-2-phenylindole (DAPI; Beyotime; Biotechnology Co. Ltd., Shanghai, China).

### Animal Care and Handling

Male C57BL/6 mice (18–20 g), an applicable animal model for BLM-induced PF model, were obtained from Shanghai Jihui Experimental Animal Breeding Co., LTD. The animals were housed in a specific pathogen-free environment and observed under a 12 h light-12 h dark cycle in a well-ventilated room at 23 ± 2°C.They were fed with standard pellet food and tap water *ad libitum*. Being approved by the Animal Care and Use Committee of Shanghai University of Traditional Chinese Medicine, the study was performed in accordance with the guidelines for care and handling of animals of the National Institute of Health (approval number PZSHUTCM220124010).

After a three-day adaptive feeding, the mice were randomized into six groups (n = 20 mice per group): (1) normal saline (NS) + vehicle control group; (2) BLM + vehicle control group, (3) BLM + PFD (300 mg/kg/day) group (14, 15); (4) BLM + TEL (10 mg/kg/day) group (16, 17); (5) BLM + High CAT (200 mg/kg/day) group; (6) BLM + Low CAT (100 mg/kg/day) group (18). On day 0, a single intratracheal instillation of BLM (5 mg/kg) was performed to induce pulmonary fibrosis in C57BL/6 mice (19). Mice in NS group (control group) received an equal volume of NS. One day after BLM induction, mice in PFD groups and TEL group were intragastrically administrated with PFD or TEL, CAT group was intragastrically administrated with high or low dose of CAT, NS group and BLM group were intragastrically administrated with equal volume of NS for 28 days. Body weight was measured every 3 days. On days 7, 14, and 28, 5, 5, and 10 mice were euthanized in each group, respectively, the mice anesthetized with pentobarbital sodium (50 mg/kg) intraperitoneally, lung tissues and blood were collected for subsequent studies.

### Histological Examination and Evaluation

Lung tissues from mice were fixed with 4% paraformaldehyde (48 h) and blocked with paraffin, sectioned (5 μM thickness) after deparaffinization in xylene. Haematoxylin and eosin (H&E) and Masson Staining Kits were used for pathological evaluation. The tissues were morphologically analyzed using a StrataFAXS II image analysis system (StrataFAXS II, Vienna, Austria).

### Western Blotting Assay

Proteins were extracted from lung tissue using RIPA lysis buffer with 2% phosphorylated proteinase inhibitor, 1% phenyl methyl sulfonyl fluoride, and 2% protease inhibitor, according to the manufacturer's protocol. 30–50 μg of protein from each sample was separated by sodium dodecyl sulfate polyacrylamide gel electrophoresis (10% SDS-PAGE) and transferred to PVDF membranes. After blocking in 5% BSA solution for 2 h at room temperature, the membranes were incubated overnight at 4°C with anti-TGF-β1, anti-Phospho-Smad2, anti-Phospho-Smad3, anti-Smad2/3, anti-Snail, anti-Angiotensin II Type 1 Receptor, United States), anti-MMP2, anti-MMP9 or anti-β-actin.

After washing three times in TBS-T, the membranes were incubated in horseradish peroxidase (HRP)-linked anti-mouse or anti-rabbit for 2 h at room temperature. Detection of protein signal and analysis was performed using Tanon 4600SF (Tiangong Technology Co., Ltd., Shanghai, China) and ImageJ software (National Institute of Mental Health, Bethesda, MD, USA), respectively.

### Immunofluorescence Assay

Immunofluorescence was used to verify E-cadherin, N-cadherin and α-SMA expression in lung tissue. Sections were incubated as previously described with anti-α-SMA or anti-E-cadherin antibody, anti-N-cadherin antibody at 4 °C overnight and then incubated with Goat anti rabbit IgG secondary antibody at 37 °C for 1.5 h. After washing, they were counterstained with 4', 6-diamidino-2-phenylindole for 10 min at room temperature in the dark. Images were captured with an immunofluorescence microscope (Leica SP8, Wetzlar, Germany).

### Hydroxyproline Assay

Collagen content was assessed by measuring the hydroxyproline (a major component of collagen) content of the tissue as previously described by Woessner (20). After lung tissue was homogenized, a HYP Kit was used to detect the HYP content, following the manufacturer's instructions.

### Ang II Assay

Angiotensin II (Ang II), a key vasoactive peptide of the RAAS, mediates pro-inflammatory and pro-fibrotic effects on the lung. After lung tissue was homogenized, an Ang II Kit was used to detect Ang II content following the manufacturer's instructions.

### Statistical Analysis

Data are shown as mean ± Standard Error of Mean (SEM). Differences between the groups were evaluated using one-way analysis of variance (ANOVA with Dunnett's *post-hoc* analysis). *P* < 0.05 was considered statistically significant. Statistical analyses and figures were obtained using GraphPad Prism Version 8.0 (GraphPad Software, San Diego, CA, USA).

## Results

### CAT Attenuates BLM Induced Pulmonary Fibrosis in Mice

**Figure 1:**
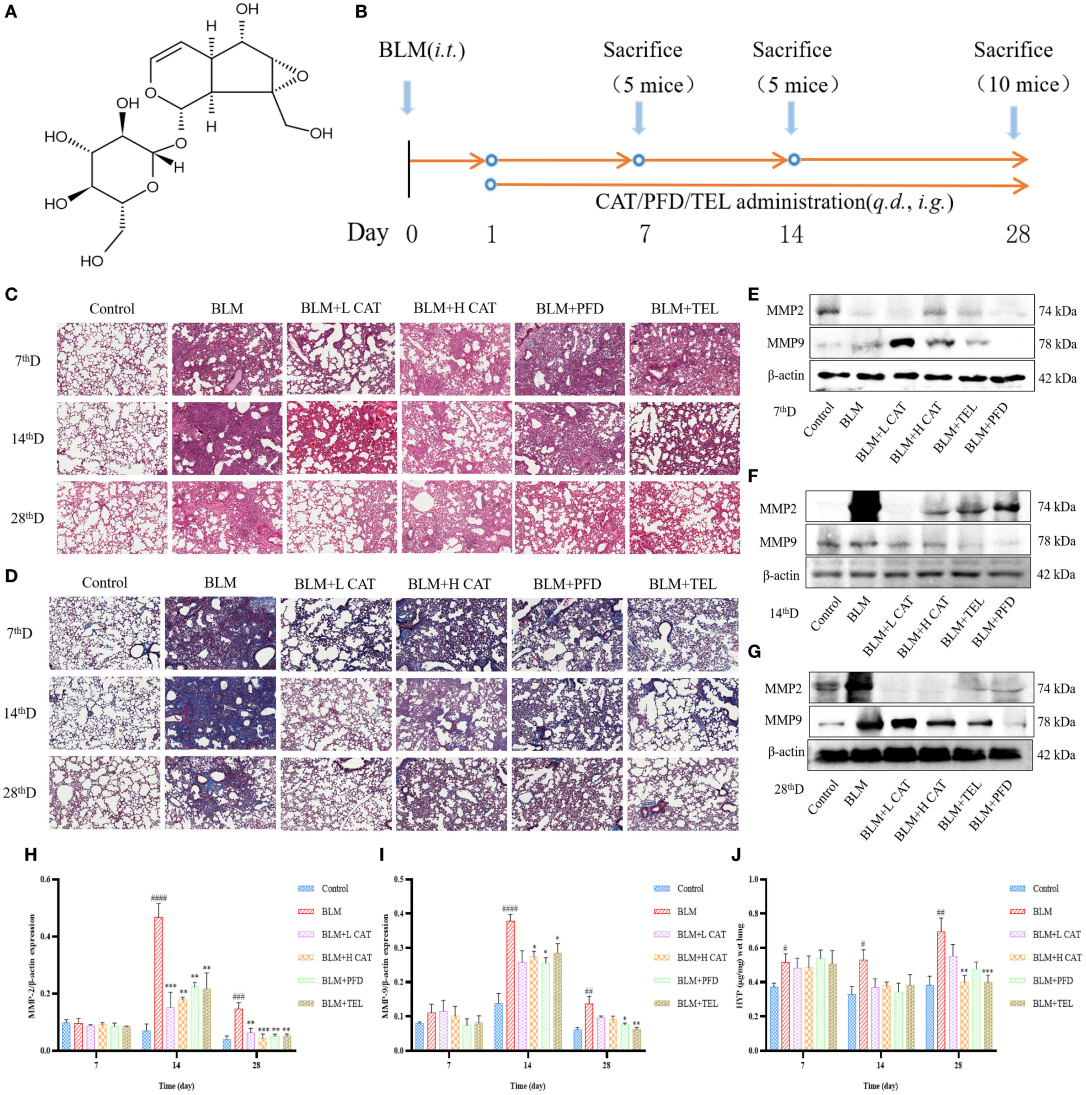
**(A)** Structure of catalpol **(B)** Animal experiment flowchart:C57BL/6 mice were treated by oral gavage with the different doses of CAT (100, 200 mg/kg/day), telmisartan (TEL) (10 mg/kg/day) and pirfenidone (PFD) (300 mg/kg/day) for 28 days followed by intratracheal instillation with BLM (5 mg/kg) or an equal volume of sterile normal saline (NS, Control group), respectively. Control group and BLM group received equal amounts of NS by oral gavage each day (*n* = 5-10, the number of mice sacrificed per group was 5, 5 and 10 on Day 7, 14 and 28 respectively). From left to right: Samples were collected at 7, 14 and 28th day after BLM administration. **(C)** Representative images of HE staining. Scale bar: 100 μm. **(D)** Representative images of Masson staining. Scale bar: 100 μm. **(E–G)** Expression of MMP2 and MMP9 in the lung tissues at 7, 14, 28d as detected by Western blot. **(H,I)** The MMP2 and MMP9 protein levels at 7, 14, 28d were analyzed by ImageJ software. **(J)** HYP in mouse serum at 7, 14, 28d was measured. ^#^*P* < 0.05, ^##^*P* < 0.01, ^###^*P* < 0.001, ^####^*P* < 0.0001 compared with the Control group, *p < 0.05, **p < 0.01, ***p < 0.001 compared with the BLM group (ANOVA with Dunnett's *post-hoc* analysis).

Intratracheal instillation of BLM in mice causes destruction of alveolar structure and the formation of fibrosis, and is a widely used model of pulmonary fibrosis. To explore the therapeutic effects of CAT on pulmonary fibrosis (21), we collected lung tissues from mice at 7, 14 and 28d of CAT treatment and performed relevant detection assessments (Figures 1A,B). H&E and Masson staining were used to examine the lung histopathological changes, which showed that after BLM modeling, the lung tissue injury was obvious, followed by an obvious interstitial inflammation and inflammatory cell infiltration, increasing with time, interstitial fibrosis was gradually aggravated, and the pathological morphological changes and collagen deposition in lungs were attenuated using CAT (Figures 1C,D). Some matrix metalloproteinases (MMPs) are overexpressed in pulmonary fibrosis and may be associated with more severe and advanced pathological stages in the lung (22, 23). In the present study, the protein expression levels of MMP2 and MMP9 in the lungs were evaluated by Western blot analysis at 7, 14, 28d. Figures 1E–I showed that compared with the Control group, the BLM-injured lung tissue showed distinct increases in the levels of MMP2 and MMP9 at 14th, 28th time point after modeling (*P* < 0.01), whereas CAT could down-regulate the protein levels of MMP2 and MMP9 in fibrotic lung tissue (*P* < 0.05, *P* < 0.01 or *P* < 0.001). To assess the effect of CAT on lung collagen and ECM, we assessed HYP (a major component of collagen) content in mouse lungs. As shown in Figure 1J, CAT obviously suppressed the expression of HYP compared with BLM group. It is known from the data that CAT could attenuate BLM induced pulmonary fibrosis progression.

### CAT Inhibited BLM Induced EMT in Mice

**Figure 2:**
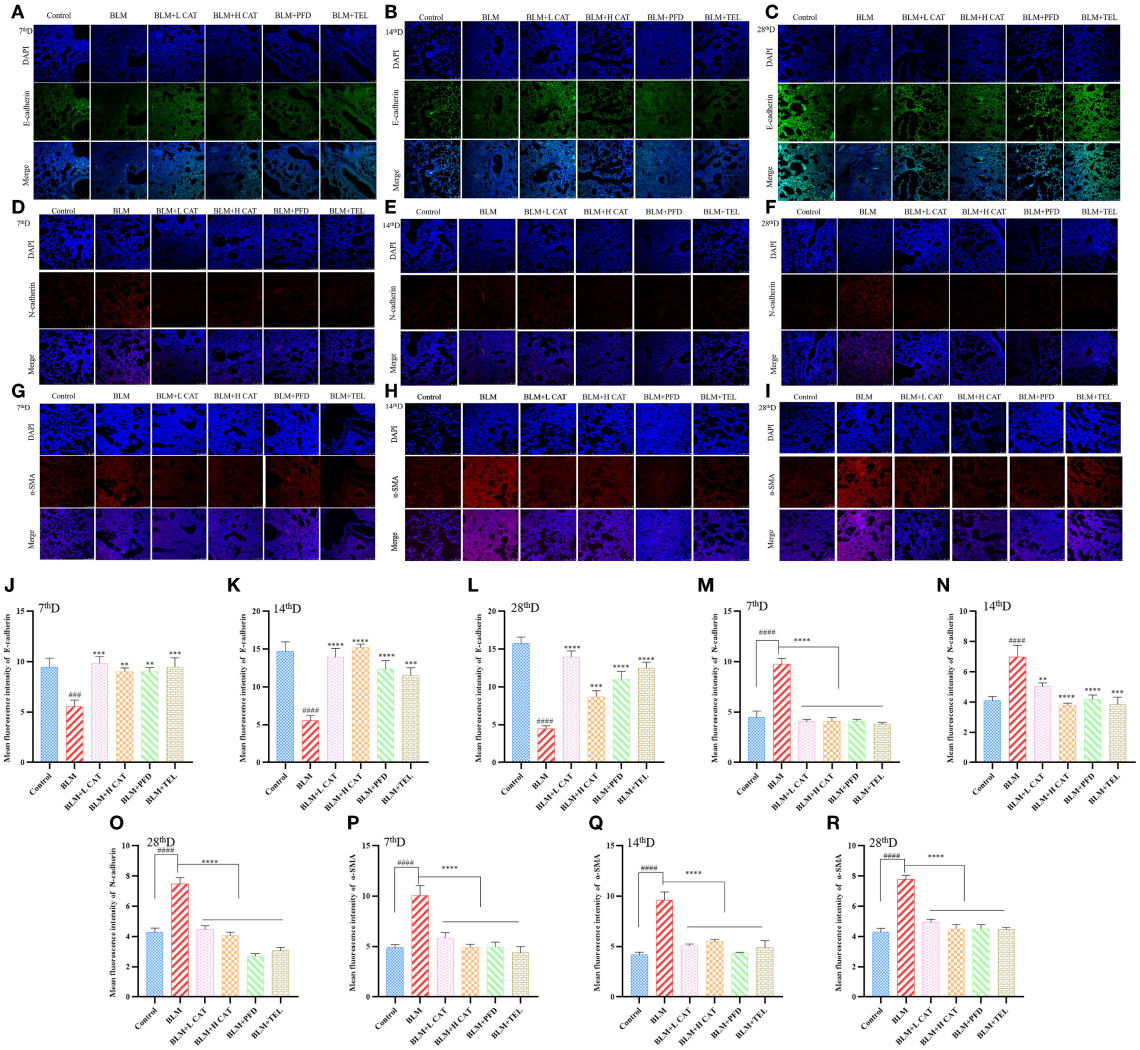
Catalpol inhibits epithelial mesenchymal transition (EMT) in bleomycin induced pulmonary fibrosis. **(A–C,J–L)** Immunofluorescence analysis of E-cadherin in lung sections at 7, 14, 28d, E-cadherin (green), DAPI (blue), the fluorescence intensity of E-cadherin was analyzed by ImageJ software. **(D–F,M–O)** Immunofluorescence analysis of N-cadherin in lung sections at 7, 14, 28d, N-cadherin (red), DAPI (blue), the fluorescence intensity of N-cadherin was analyzed by ImageJ software. **(G–I,P–R)** Immunofluorescence analysis of α-SMA in lung sections at 7, 14, 28d, α-SMA (red), DAPI (blue), the fluorescence intensity of α-SMA was analyzed by ImageJ software. ^###^*P* < 0.001, ^####^*P* < 0.0001 compared with the Control group, **p < 0.01, ***p < 0.001, ****p < 0.0001 compared with the BLM group (ANOVA with Dunnett's *post-hoc* analysis).

Previous studies have shown that EMT plays an important role in fibrosis in multiple organs, such as promoting epithelial to fibroblast differentiation and participating in the constitution of fibroblast/myofibroblast foci (24, 25). We further evaluated the role of CAT on EMT progression during BLM induced pulmonary fibrosis in mice. Immunofluorescence analyses revealed that BLM administration in mouse lungs resulted in a significant decrease in E-cadherin expression along with an increase in N-cadherin and α smooth muscle actin (α-SMA), suggesting an epithelial mesenchymal phenotype transition and an increase in myofibroblasts during PF. After CAT administration, the expression of E-cadherin increased, while the expression of N-Cadherin and α-SMA decreased (*P* < 0.01, *P* < 0.001 or *P* < 0.0001) and EMT was inhibited in the lungs of mice compared with that in model group (Figures 2A–R).

### CAT Attenuates Lung Fibrosis in Mice *via* the Ang II / AT_1_ Axis

**Figure 3:**
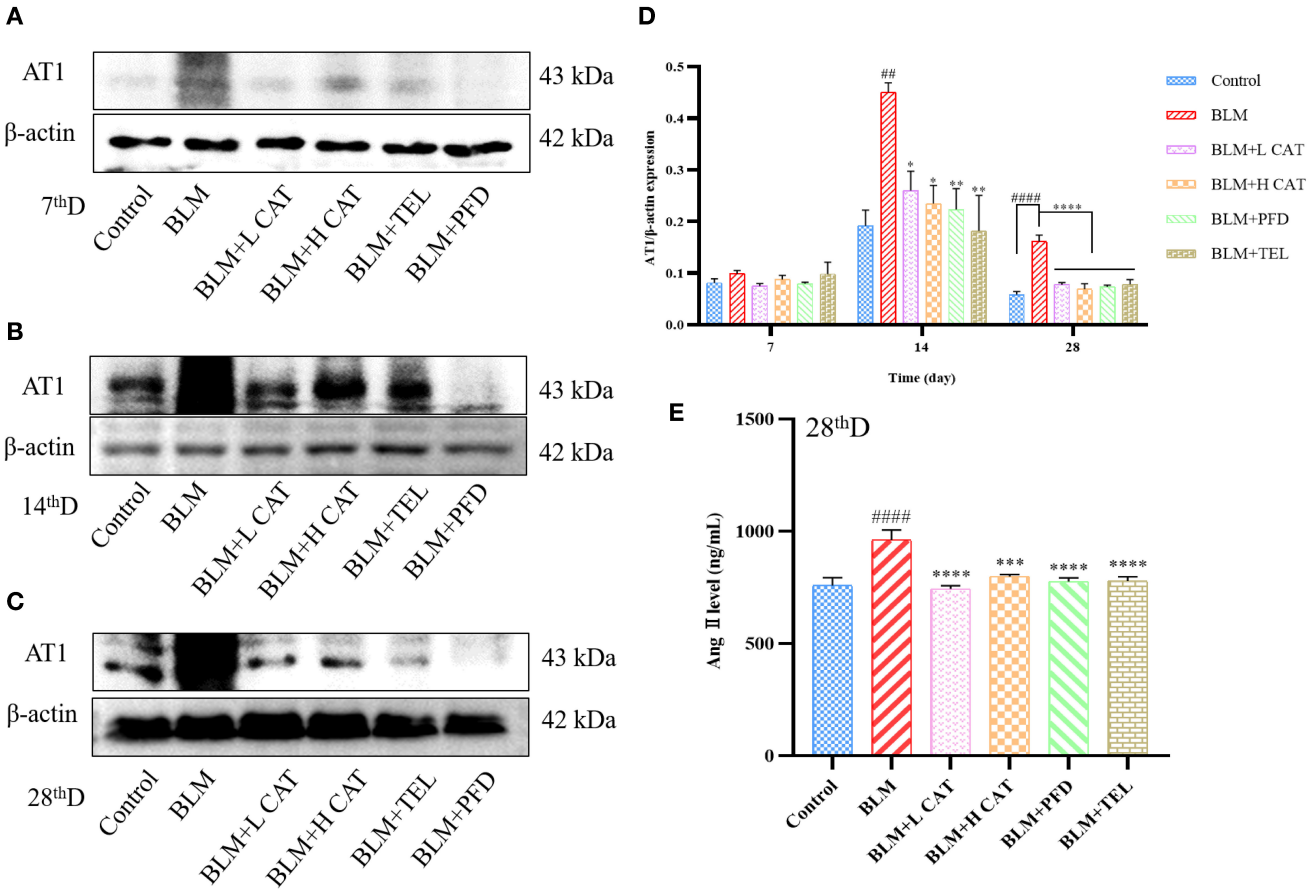
Catalpol inhibits the up-regulation of Ang II and AT_1_ in bleomycin-induced pulmonary fibrosis in mice. **(A–D)** Representative Western blots of AT_1_ and gray scale quantified data of AT_1_-to-β-actin expression ratio at 7, 14, 28d. **(E)** Ang II in mouse serum at 28 day was measured. ^##^*P* < 0.01, ^####^*P* < 0.0001 compared with the Control group, *p < 0.05, **p < 0.01, ***p < 0.001 ****p < 0.0001 compared with the BLM group (ANOVA with Dunnett's *post-hoc* analysis).

The physiological actions of Ang II are almost all mediated by type 1 angiotensin receptor (AT_1_) on the cytoplasmic membrane representations, thereby promoting multiple signaling pathways that exert physiological functions. Study has found that not only Ang II has a promoting effect on lung fibroblast proliferation *in vitro*, but also AT_1_ as well as TGF-β1 overexpression was found in the model of BLM induced lung fibrosis, accompanied by enhanced collagen synthesis with collagen deposition. Candessartan, an AT_1_ receptor antagonist, could reduce TGF-β1 expression and the degree of fibrosis, indicating that Ang II might promote TGF-β1 expression and aggravate the degree of pulmonary fibrosis (26). Figures 3A–E showed that treatment with CAT significantly decreased the amount of Ang II in serum (*P* < 0.001 or *P* < 0.0001) and protein of AT_1_ in lung tissue.

### CAT Attenuates Pulmonary Fibrosis in Mice *via* TGF-β/Smads Signaling Pathway

**Figure 4:**
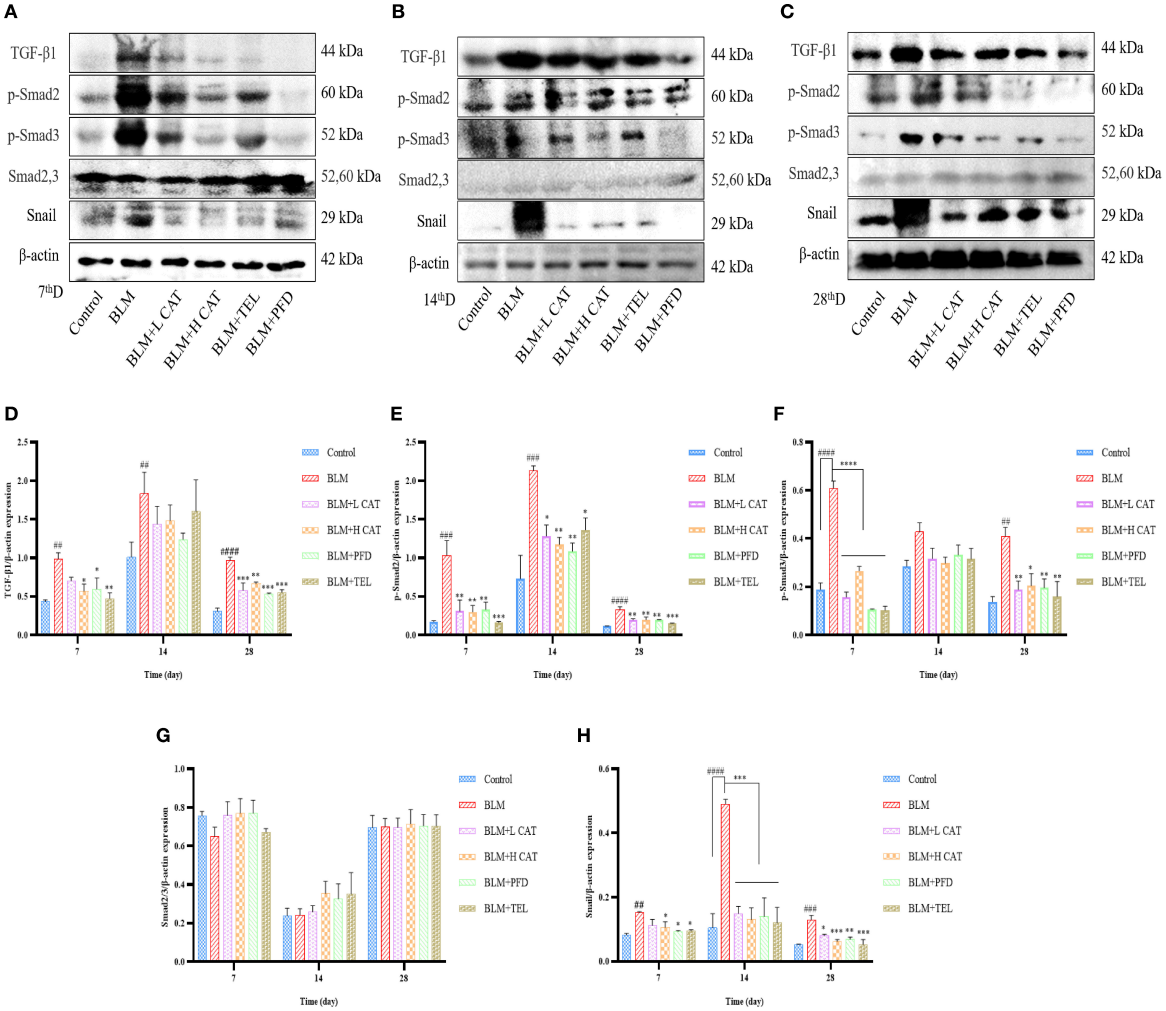
Effect of catalpol on TGF-β/Smad2/3 signaling pathway. **(A–C)** Western blotting was conducted to detect the expression levels of TGF-β1, Smad2,3, p-Smad2, p-Smad3 and Snail in different lung tissue groups at 7, 14, 28d. **(D–H)** Densitometry data of TGF-β1-to-β-actin, p-Smad2-to-β-actin, p-Smad3-to-β-actin, Smad2/3-to-β-actin, Snail-to-β-actin expression ratio at 7, 14, 28d. ^##^*P* < 0.01, ^###^*P* < 0.001, ^####^*P* < 0.0001 compared with the Control group, *p < 0.05, **p < 0.01, ***p < 0.001,****p < 0.0001 compared with the BLM group (ANOVA with Dunnett's *post-hoc* analysis).

Small molecules against decapentaplegic homologs (Smad) are classic mediators in the TGF-β signaling pathway and regulate the transcription of a variety of genes, such as AP-1 and snail, which promote pulmonary fibrosis. To determine whether CAT played an important role in the TGF-β signaling pathway in pulmonary fibrosis, we measured the protein expression levels of TGF-β1, Smad2/3, p-Smad2, p-Smad3, Smad7 as well as Snail. As shown in Figures 4A–H, TGF-β1, p-Smad2, p-Smad3 and Snail were significantly up-regulated in model group. After CAT treatment, the level of phospho-Smad2 was significantly decreased compared with that of model group (*P* < 0.05). CAT probably acts through TGF-β Signaling pathway to inhibit pulmonary fibrosis development.

**Figure 5:**
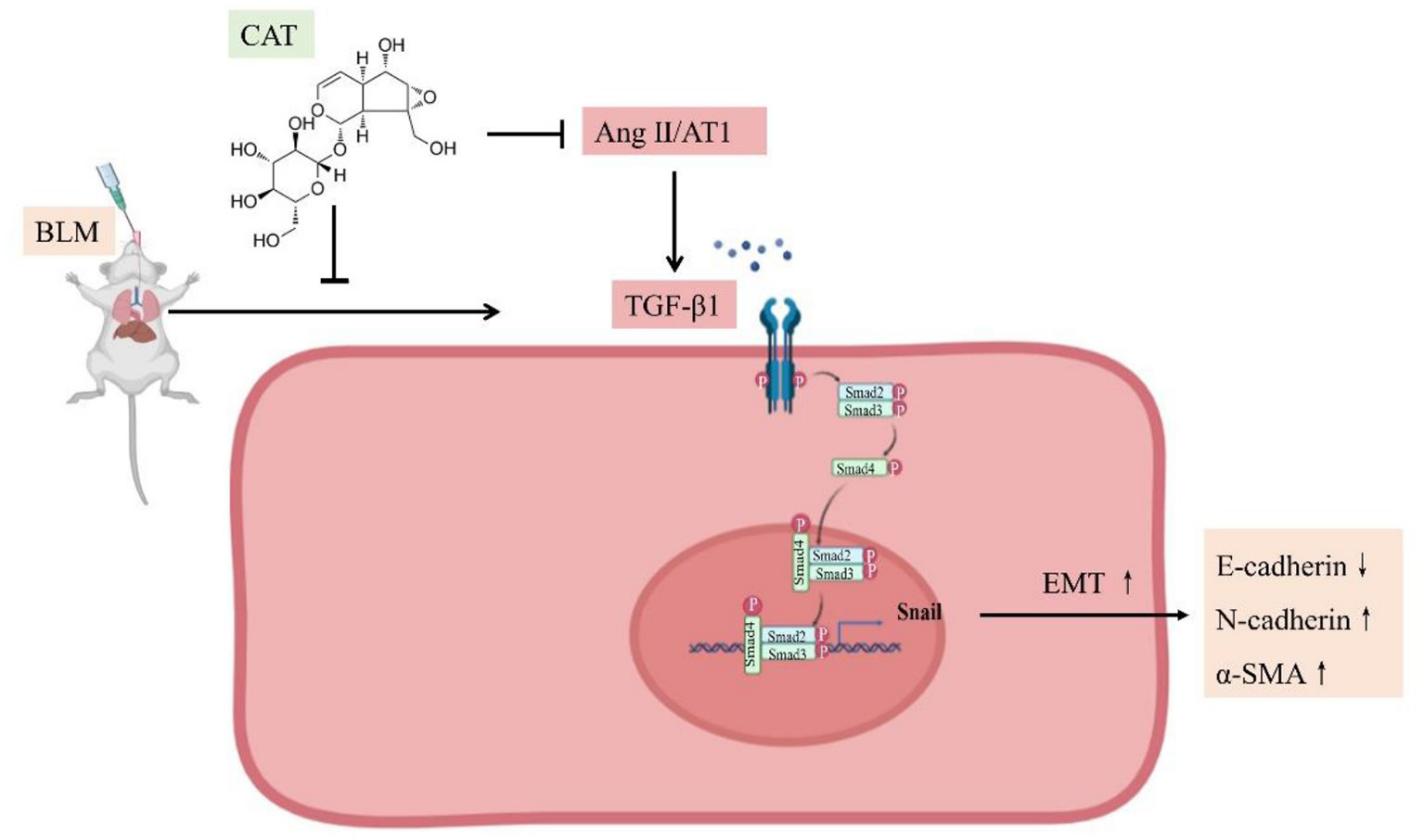
Schematic representation of catalpol regulation of the Ang II/AT_1_ and TGF-β/Smad pathway in pulmonary fibrosis.

Given all the above data, we conclude that catalpol attenuates pulmonary fibrosis by inhibiting Ang II/AT_1_ and TGF-β/Smad-mediated epithelial mesenchymal transition (Figure 5).

## Discussion

Following damage to lung tissue, barrier integrity is generally repaired through the formation of a provisional matrix, transmigration of myofibroblasts and contraction of the wound. Then epithelial cells regenerate and remodel, remove debris and extracellular matrix (ECM) to achieve normal lung healing (27). However, excessive, persistent damage to the alveolar epithelium and/or abnormal wound healing contribute to a dysfunctional, often overexuberant, repair process. The lung develops an inflammatory response in which macrophages and immune cells infiltrate, and produce and release a large number of cytokines and growth factors. Subsequent transformation of alveolar epithelial cells to a mesenchymal phenotype, fibroblast and myofibroblast foci formation, and excessive accumulation of ECM and collagen result in scar formation and destruction of lung architecture, impairing lung function (10, 28).

Renin-angiotensin system (RAS) is the main endocrine regulatory system of the body. Renin (EC 3.4.23.15) is an aspartyl protease that can catalyze the specific cleavage of angiotensinogen into the decapeptide Ang I. Then, angiotensin converting enzyme (ACE) converts Ang I into the biologically active octapeptide Ang II, and regulate pressure as well as sodium and water homeostasis and so on (29). All major components of the RAS exhibit profibrotic activity, such as Angiotensin converting enzyme (ACE) and angiotensinogen may contribute to increased Ang II production, and Ang II plays important roles in the development of renal, hepatic, as well as pulmonary fibrosis (30, 31). Ang II is hydrolyzed from Ang I under the action of ACE, and the binding of Ang II with AT_1_ may be involved in the process of pulmonary fibrosis as a pro-fibrosis mediating factor. Fibroblasts, which make up about 40% of lung cells, are responsible for the deposition of collagen and other ECM proteins. Myofibroblasts expressing α-SMA are extracellular in PF. It has been demonstrated *in vitro* that Ang II can activate the AT_1_ to induce human lung fibroblast growth, proliferation, and to mediate the transition of fibroblast phenotype to myofibroblasts as well as procollagen production. And Ang II may also be one of several growth factors that induce and enhance TGF-β and connective tissue growth factor (CTGF) expression at the site of injury (26, 32, 33). Telmisartan, one of the positive controls used in this study, was an Ang II receptor antagonist.

BLM, an agent that induces experimental pulmonary fibrosis, has been described in a variety of experimental animal model and this BLM-induced model has the advantages of being simple to perform, widely available and reproducible (21). Intratracheal instillation of BLM in mice causes inflammatory and fibrotic responses within a short period of time. The first 7 days after modeling of BLM induced an inflammatory response characterized by increased levels of proinflammatory cytokines (interleukin-1 β, tumor necrosis factor-α, IL-6, interferon-γ). Inflammation subsequently resolves and fibrosis is detected, in which the expression of profibrotic markers (TGF-β1, Fibronectin, procollagen-1) increased and peaked around day 14. The stage of fibrosis can be maintained for 3–4 weeks after BLM modeling (34). In this experiment, three time points (7, 14, and 28d) were designed to observe the effect of CAT on pulmonary fibrosis at each time point. In this study, Ang II combined with AT_1_ was up-regulated in BLM-induced pulmonary fibrosis mice model at 14 and 28 days, and in the CAT treated group, Ang II and AT_1_ was down regulated significantly compared with that of the model group. CAT may attenuate pulmonary fibrosis through Ang II/AT_1_ axis.

EMT is a crucial step in the process of lung development and many lung diseases (pulmonary fibrosis, chronic obstructive pulmonary disease (COPD), lung cancer). EMT is characterized by epithelial cells losing their characteristics of cell-cell adhesion and apical basal polarity and by gaining some mesenchymal characteristics for migration, invasion, and production of ECM components (35, 36). EMT in organ fibrosis is a major provider of pathogenic mesenchymal cell types with migratory and invasive behavior, such as myofibroblasts (37). Type II EMT is associated with wound healing, tissue regeneration and organ fibrosis, during lung fibrosis, it is generally defined by the detection of several biomarkers that reflect the loss of epithelial phenotype and the gain of mesenchymal phenotype. The changes of these biomarkers include decreased expression of the epithelial cell-cell adhesion molecule CDH1 (E-cadherin) and/or increased expression of mesenchymal markers (CDH2 (N-cadherin), VIM (vimentin), and/or α-SMA), as well as the change of ECM components (fibronectin and collagen) or degrading enzymes (MMP2 and MMP9) (38–40).

TGF-β, a key factor in fibrotic diseases and one of the most studied cytokines, it has various biological effects, such as increasing ECM deposition, recruiting inflammatory cells, contributing to fibrocyte differentiation and epithelial–mesenchymal transition (EMT) (41, 42). TGF-β induces EMT development and progression of lung fibrosis mainly through its canonical Smad dependent pathway. TGF-β family cytokines induce serine/threonine kinase type receptors on the cell membrane to form a functional complex that phosphorylates receptor regulated Smad2 and Smad3, and binds to Smad4, which in turn translocates into the nucleus and participates in transcriptional regulation of target genes (43). Among them, the transcription factors Snail1 and Snail2 downregulate E-cadherin expression and increase the expression of mesenchymal proteins such as N-cadherin, fibronectin and metalloproteinases (27).

The results of pulmonary TGF-β expression in this study may reflect the degree of lung inflammation/EMT in each experimental group. TGF-β and Smad2/3 were significantly upregulated in the model mice, indicating that TGF-β/Smad2/3 pathway is involved in BLM-induced pulmonary fibrosis. After treatment with CAT, the levels of TGF-β, phospho-Smad, as well as the expression of Snail were all decreased. Moreover, CAT can also up-regulate the epithelial cell marker E-cadherin and down-regulate the mesenchymal marker N-cadherin and α-SMA. Our study showed that CAT slowed down EMT progression and reduced myofibroblast proliferation in lung fibrosis. In addition, studies have shown that MMP2 and MMP9 can promote aberrant epithelial repair in fibrotic lungs, degrade ECM and thereby promote aberrant epithelial cell migration as well as promote EMT (22). CAT significantly reduced the MMP2 and MMP9 levels in fibrotic lungs and inhibited the progression of pulmonary fibrosis. CAT may inhibit TGF-β/Smad signaling pathway to attenuate EMT progression of pulmonary fibrosis. Ang II and TGF-β have been proved to promote pulmonary fibrosis. Some studies have shown that there is signal crosstalk between them (44). Ang II binds to AT_1_ on the cell membrane and phosphorylates Smad2/3 through the ERK/P38/MAPK pathway. It is then combined with Smad4 and is transferred into the nucleus as a transcription factor to regulate the transcription of TGF-β, procollagen I, procollagen III and fibronectin (33). In addition, Ang II-AT_1_ could also directly activate TGF-β signal transduction and induce collagen synthesis in human fetal lung fibroblasts (27). Similarly, Accumulating evidence shows that Ang II stimulates macrophages to release AT_1_-enriched exosomes, which promotes fibroblasts activation and lung fibrosis via transforming growth factor-β (TGF-β)/smad2/3 pathway (25, 45–47). In our study, treatment with CAT decreased Ang II and AT_1_ as well as TGF-β/Smad2/3, indicating that CAT may attenuate the progression of BLM-induced lung fibrosis in mice partly by inhibiting phosphorylation of Smad2/3 via decreasing Ang II expression.

## Conclusion

TGF-β1 and Ang II is a critical mediator of tissue fibrosis in disease. These pathways were disease-relevant because the levels of TGF-β and Ang II were increased and positively correlated with Smad2/3 in tissues from patients with idiopathic pulmonary fibrosis or scleroderma-associated interstitial lung disease. Chemicals capable of suppressing TGF-β1-induced production of collagen represent good candidates to treat IPF. Our study points out that the mechanism of CAT protecting against pulmonary fibrosis induced by BLM in mice is related to the regulation of TGF-β1, Smad2, Smad3, Ang II, AT_1_, E-cadherin, N-cadherin and α-SMA as well as the phosphorylation of Smad2 and Smad3, also it is given orally which makes it easier to put into clinical practice. In summary, our study points out that catalpol is able to inhibit pulmonary fibrosis, and the mechanism may lie in down-regulating Ang II and AT_1_ as well as inhibiting EMT progression through the TGF-β/Smad2/3 signaling pathway.

## Data Availability Statement

The raw data supporting the conclusions of this article will be made available by the authors, without undue reservation.

## Ethics Statement

The animal study was reviewed and approved by Animal Care and Use Committee of Shanghai University of Traditional Chinese Medicine.

## Author Contributions

Y-HS: conception and design, conceptualization, methodology, data curation, visualization, investigation, supervision, validation, writing-reviewing and editing, project administration, and funding acquisition. QY: software, methodology, data curation, data and image processing, visualization, and validation. D-WZ: investigation, conceptualization, methodology, data curation, visualization, validation, and writing-original draft preparation. YZ: investigation, data curation, data and image processing, and visualization. KW: designed and performed histological experiment. P-LR: carried out a portion of the animal experiments. All authors contributed to and have approved the final manuscript.

## Funding

This work was supported by the high-level construction funds of Shanghai University of Traditional Chinese Medicine.

## Conflict of Interest

The authors declare that the research was conducted in the absence of any commercial or financial relationships that could be construed as a potential conflict of interest.

## Publisher's Note

All claims expressed in this article are solely those of the authors and do not necessarily represent those of their affiliated organizations, or those of the publisher, the editors and the reviewers. Any product that may be evaluated in this article, or claim that may be made by its manufacturer, is not guaranteed or endorsed by the publisher.
