# Supplementary material for: Catalpol Attenuates Pulmonary Fibrosis by Inhibiting Ang II/AT1 and TGF-β/Smad-Mediated Epithelial Mesenchymal Transition
Source: Front Med (Lausanne). 2022 May 24;9:878601. doi: 10.3389/fmed.2022.878601 (PMC9171363; doi:10.3389/fmed.2022.878601)

7day-Control

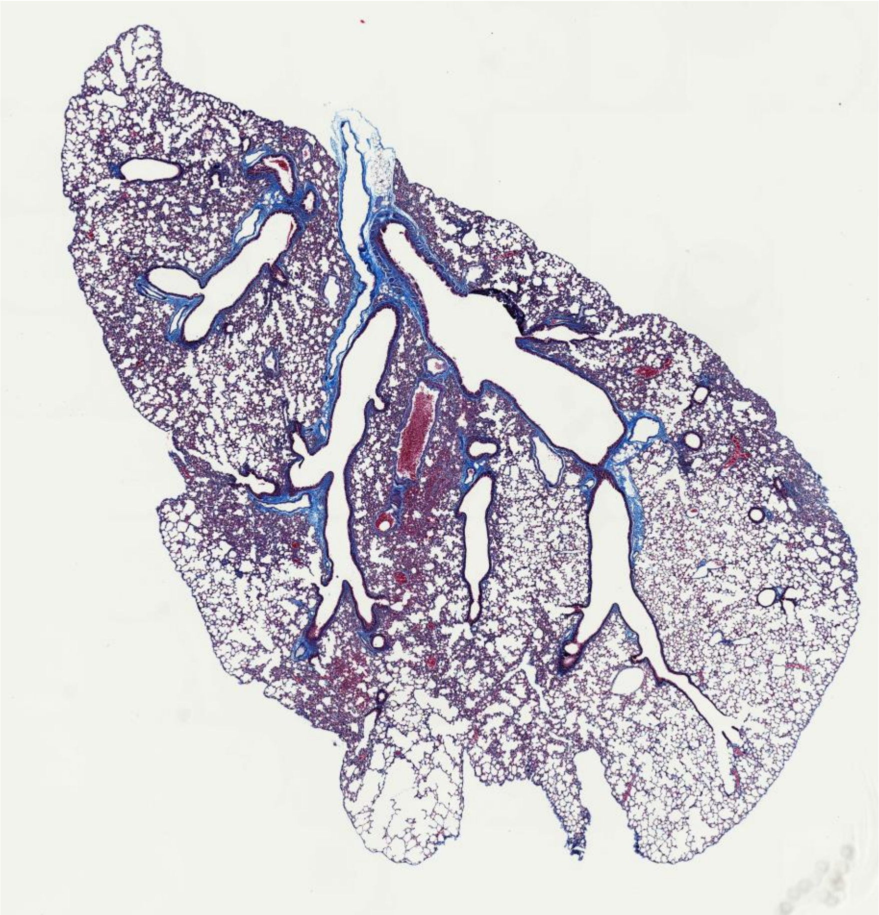

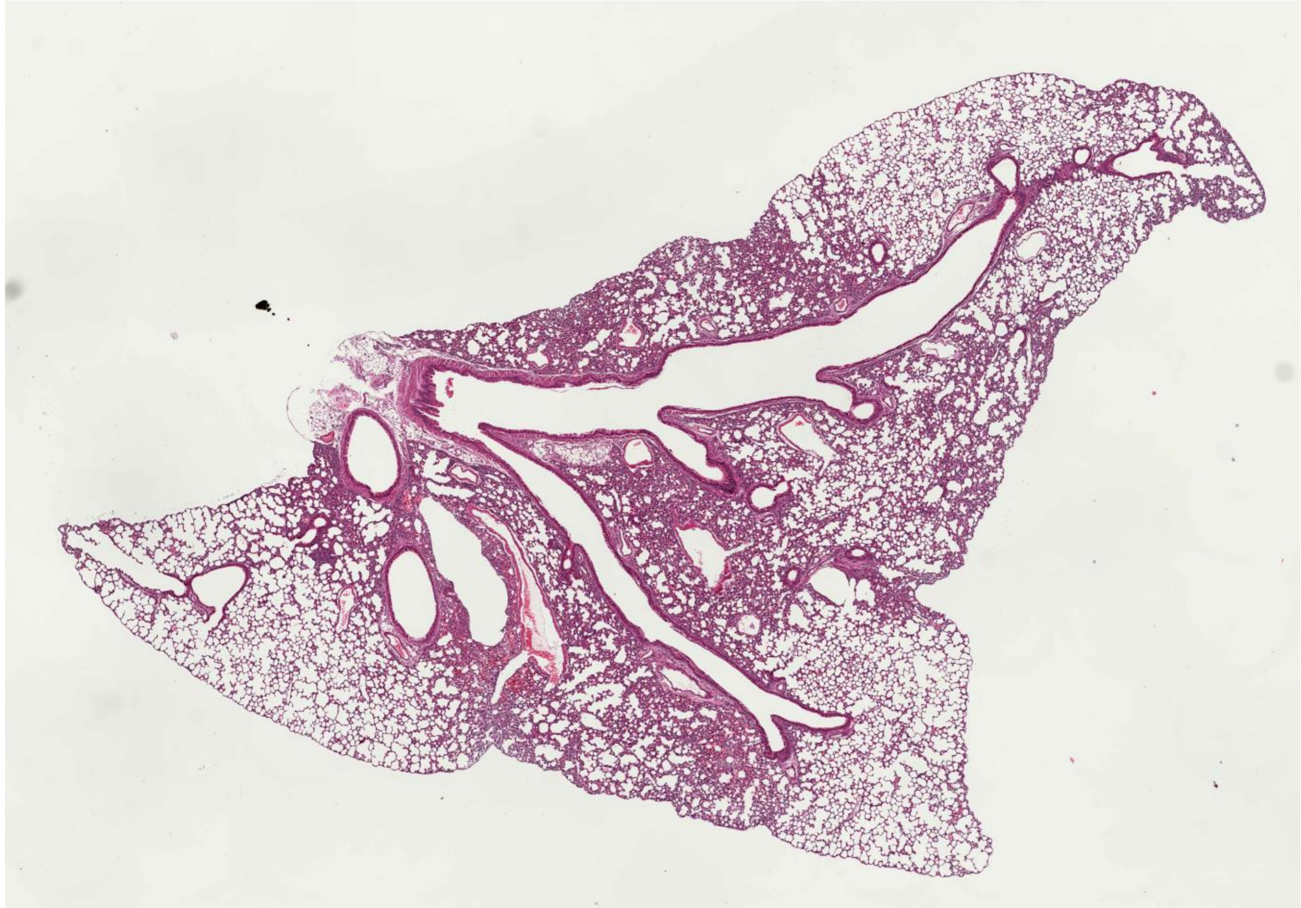

7day-BLM+H CAT

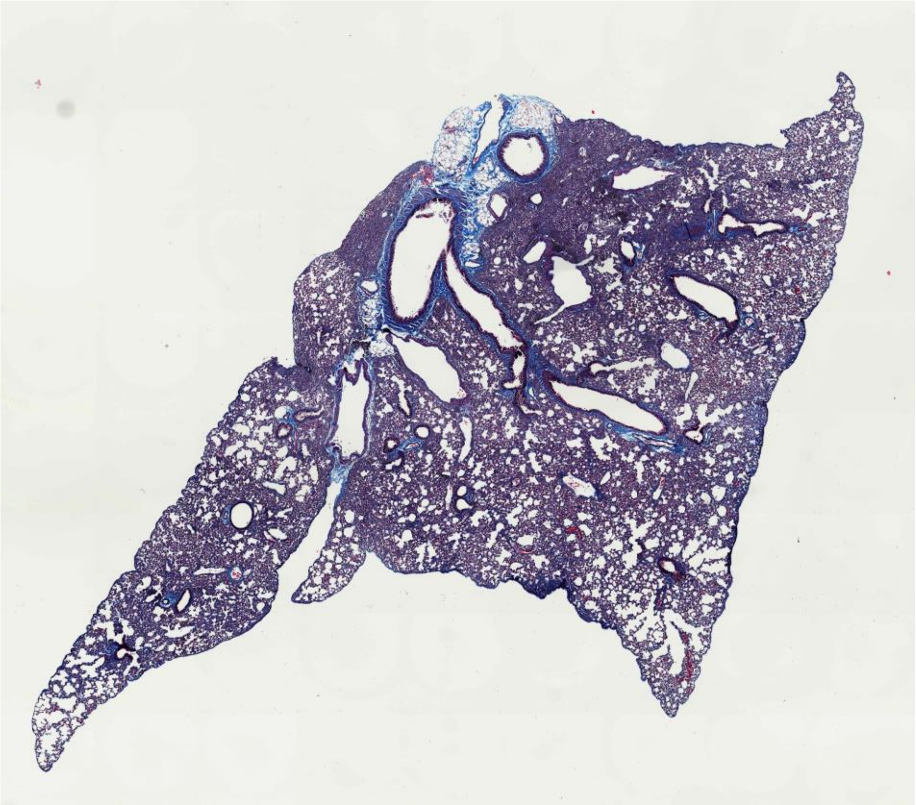

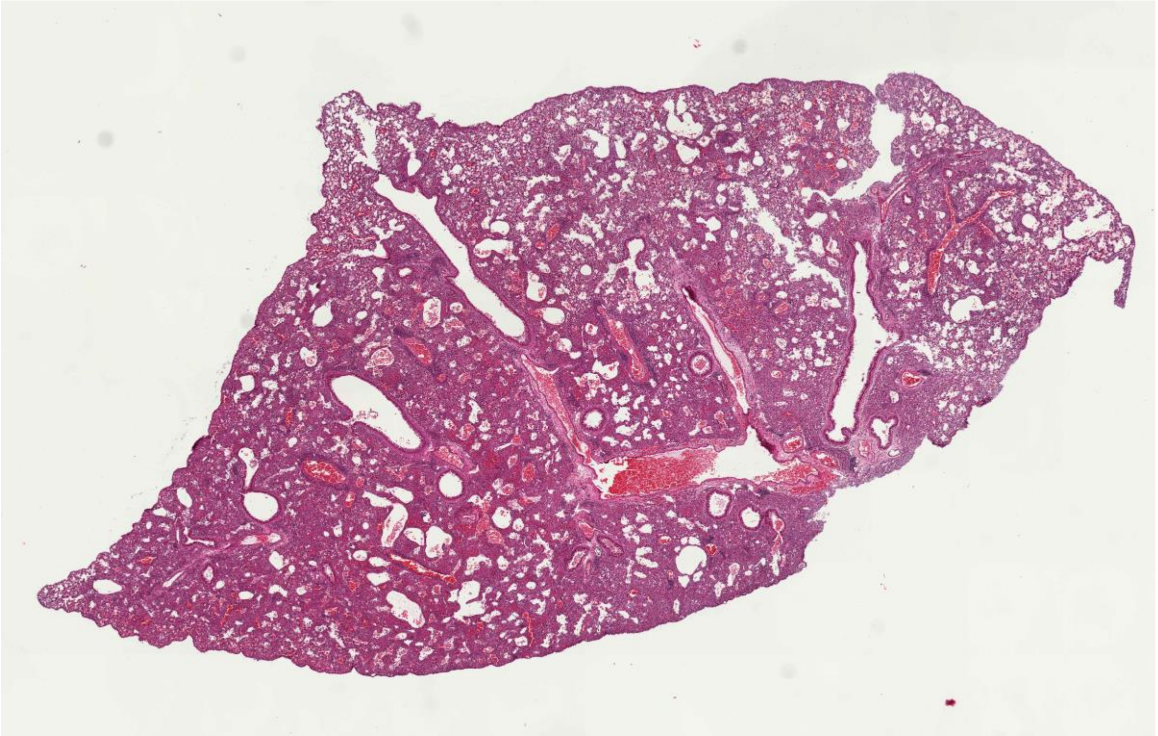

7day-BLM+L CAT

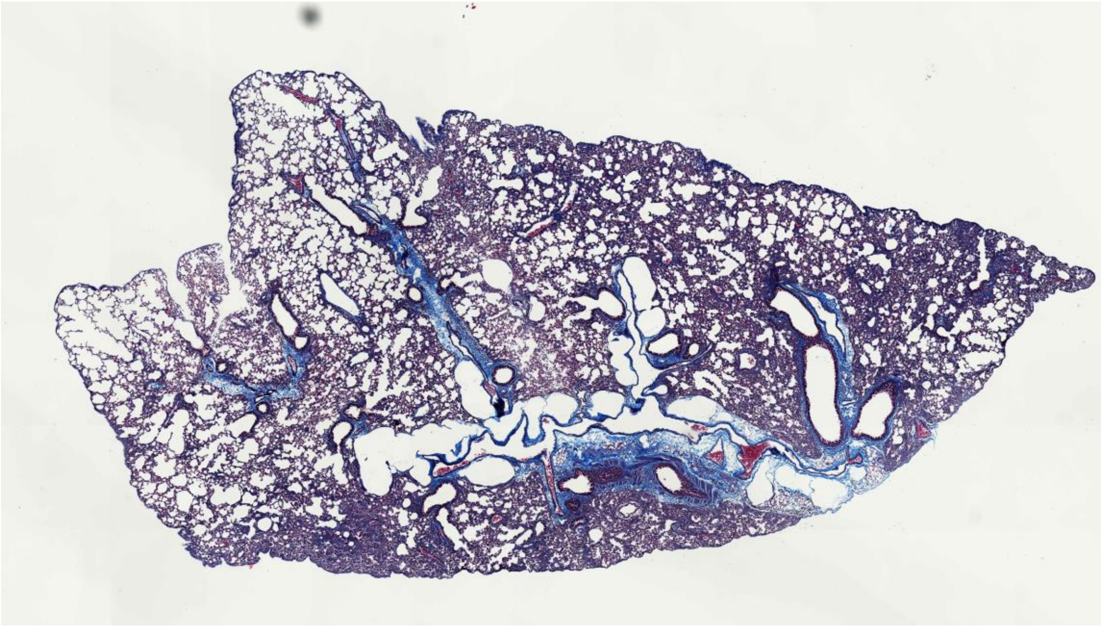

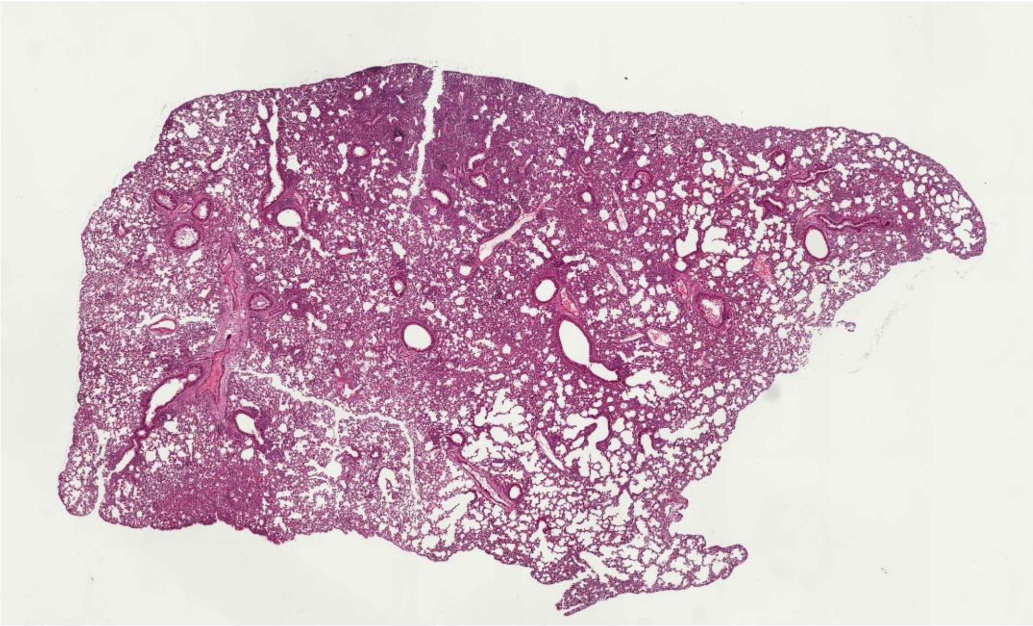

7day-BLM

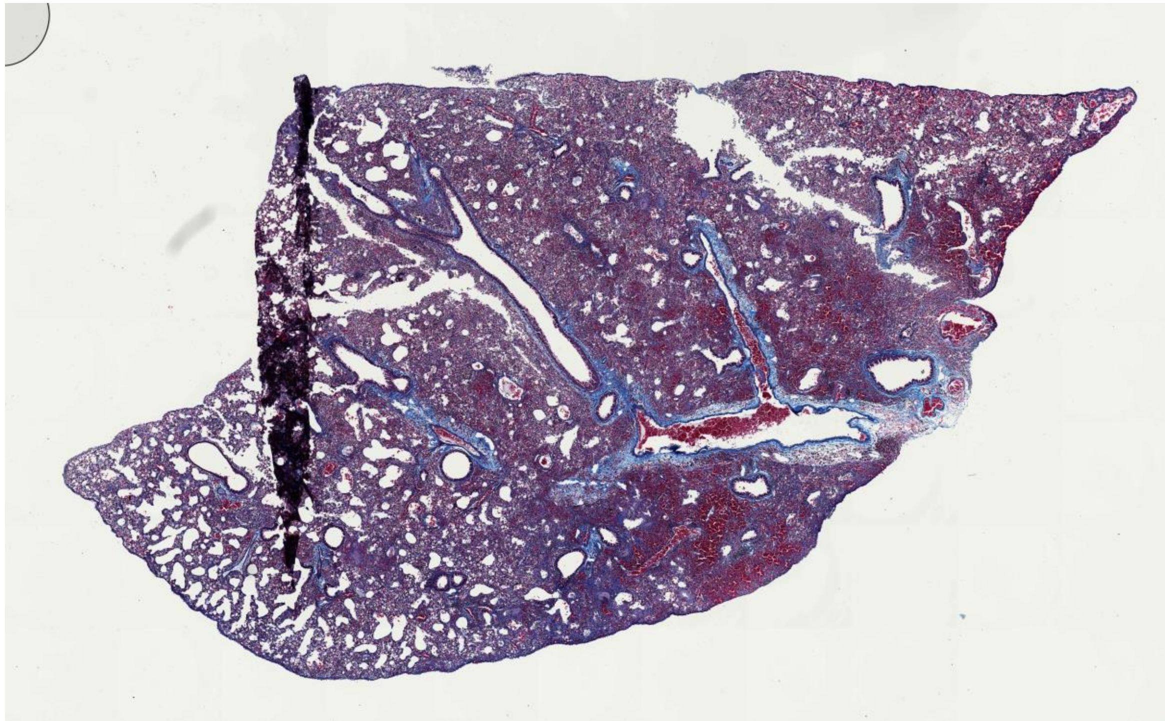

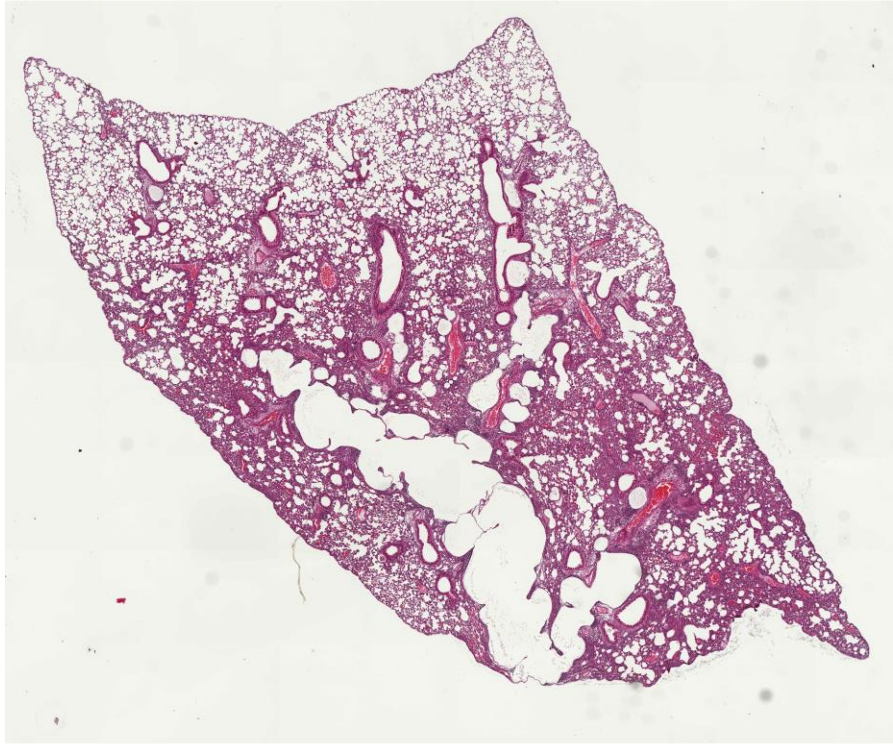

7day-BLM+PFD

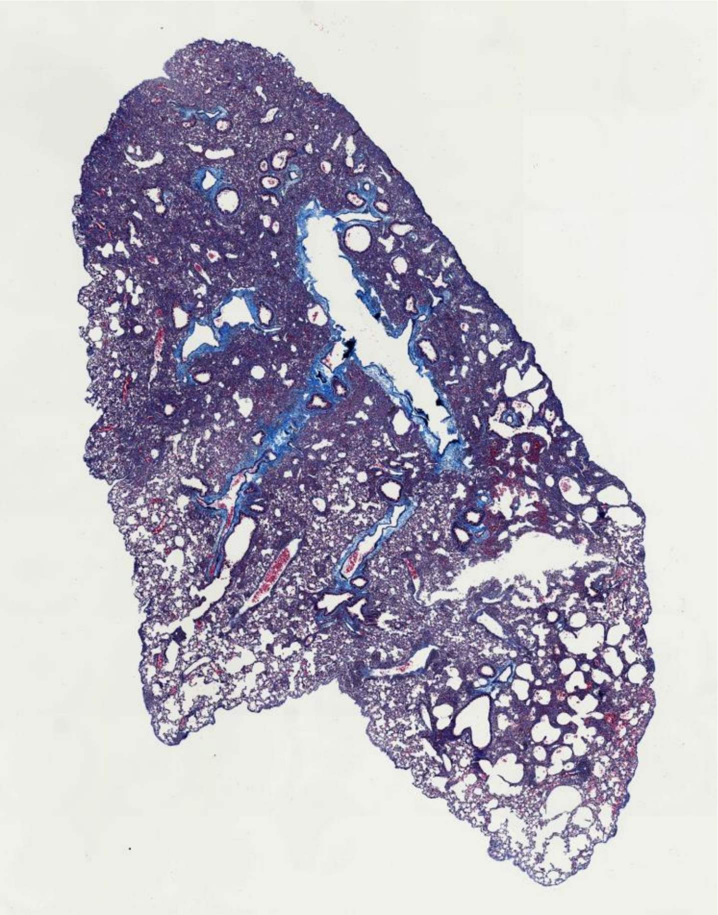

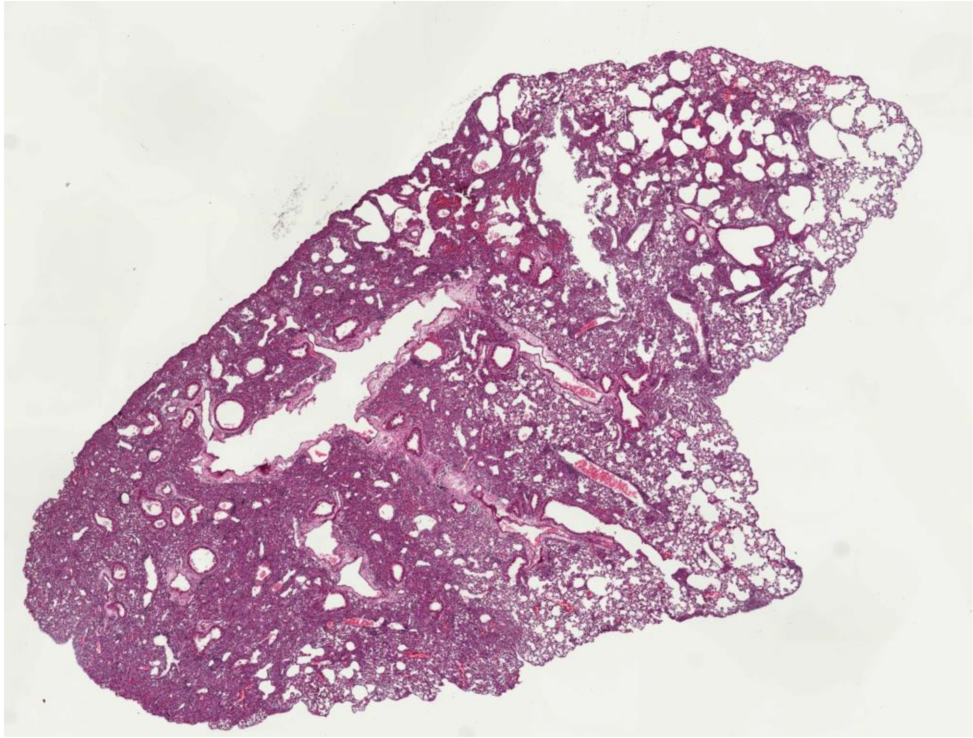

7day-BLM+TEL

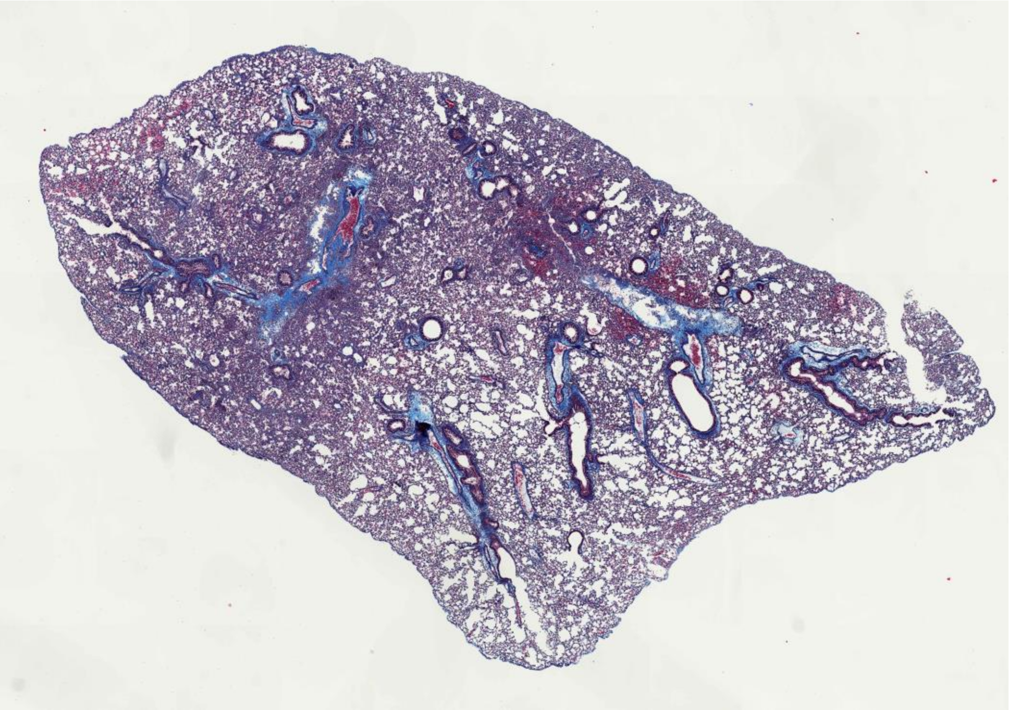

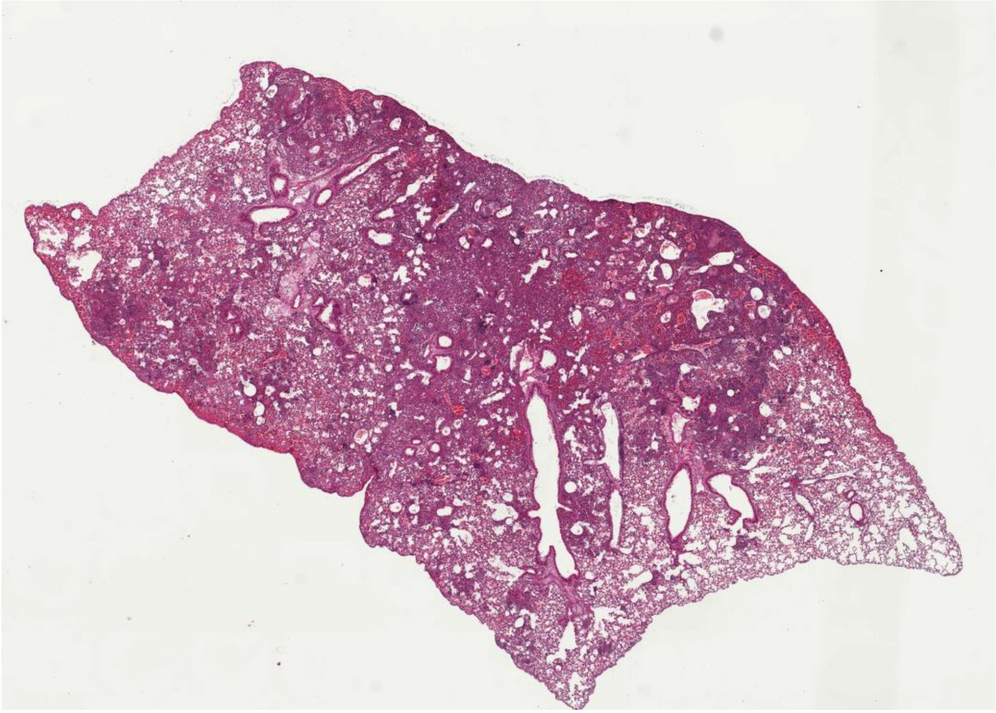

14day-Control

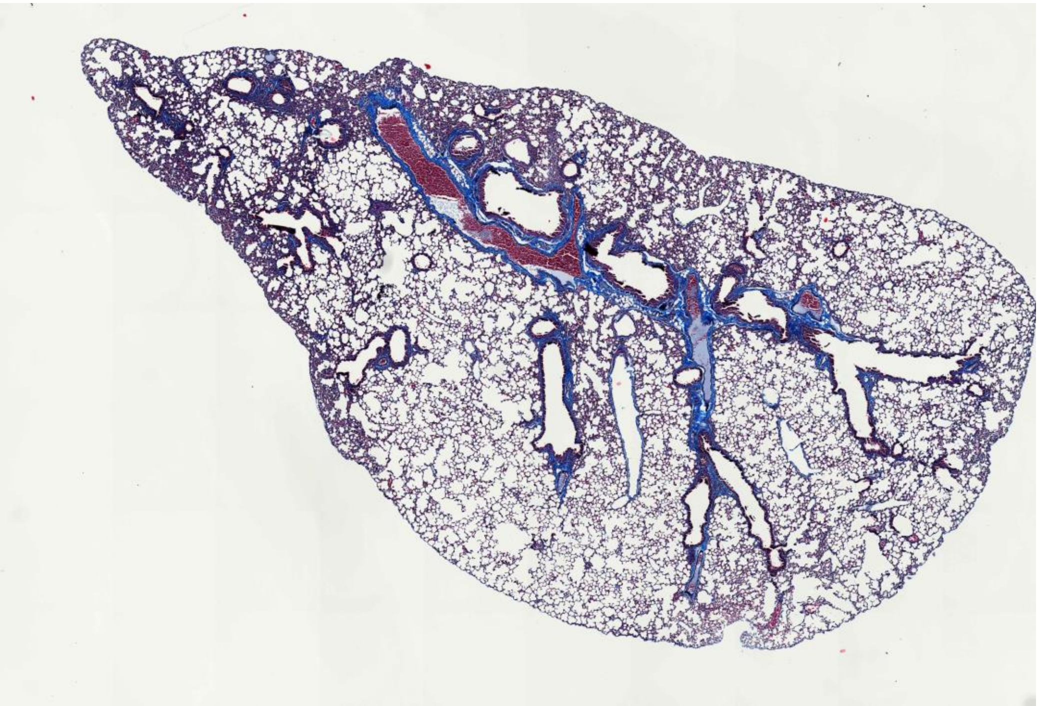

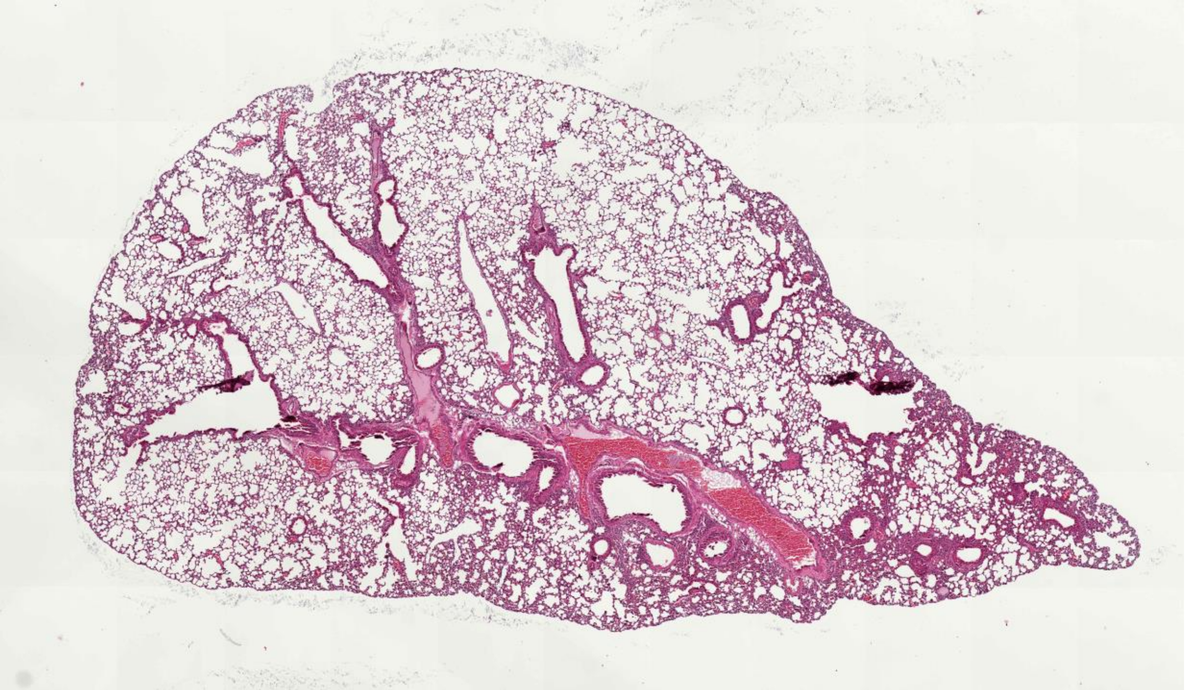

14day-BLM+H CAT

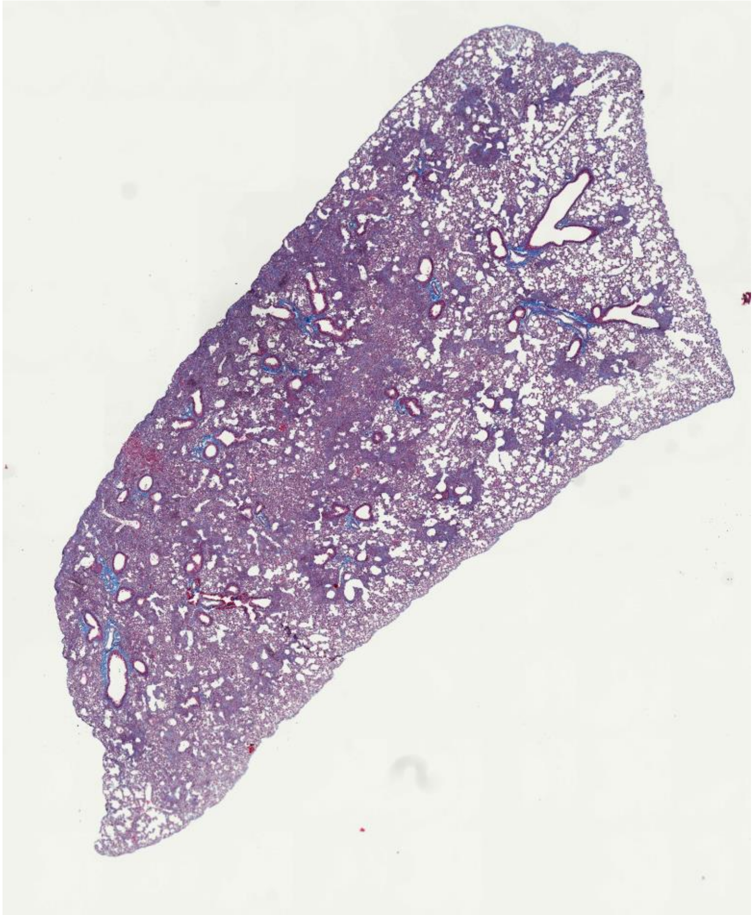

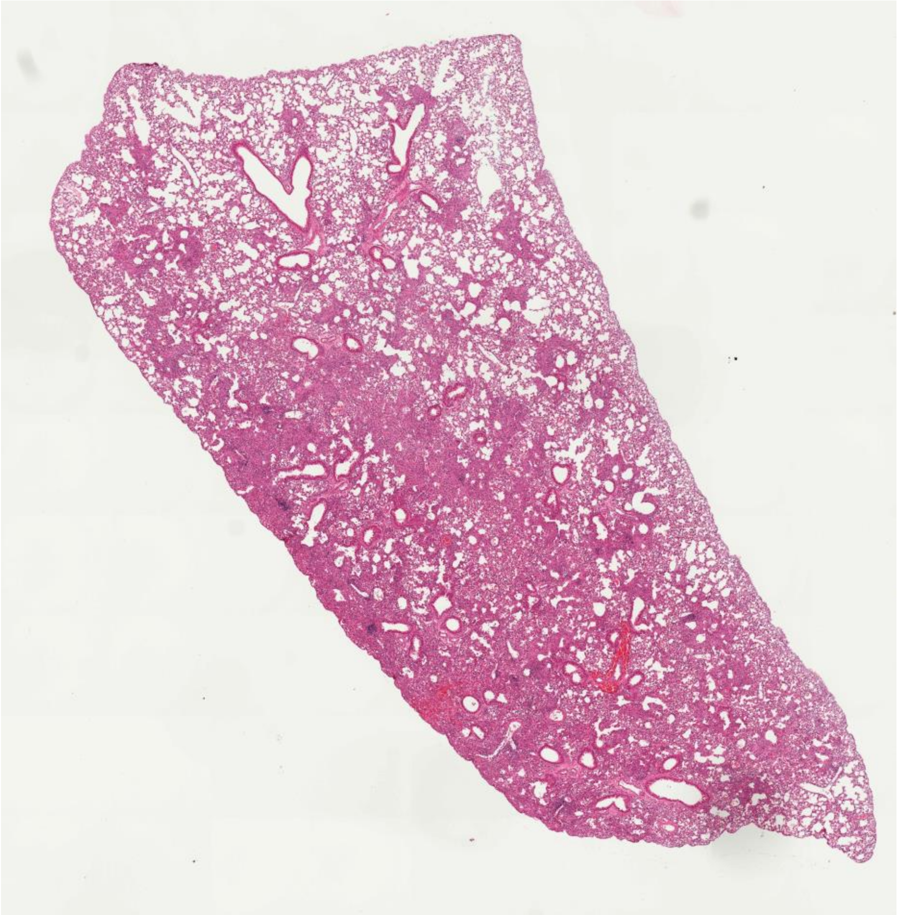

14day-BLM+L CAT

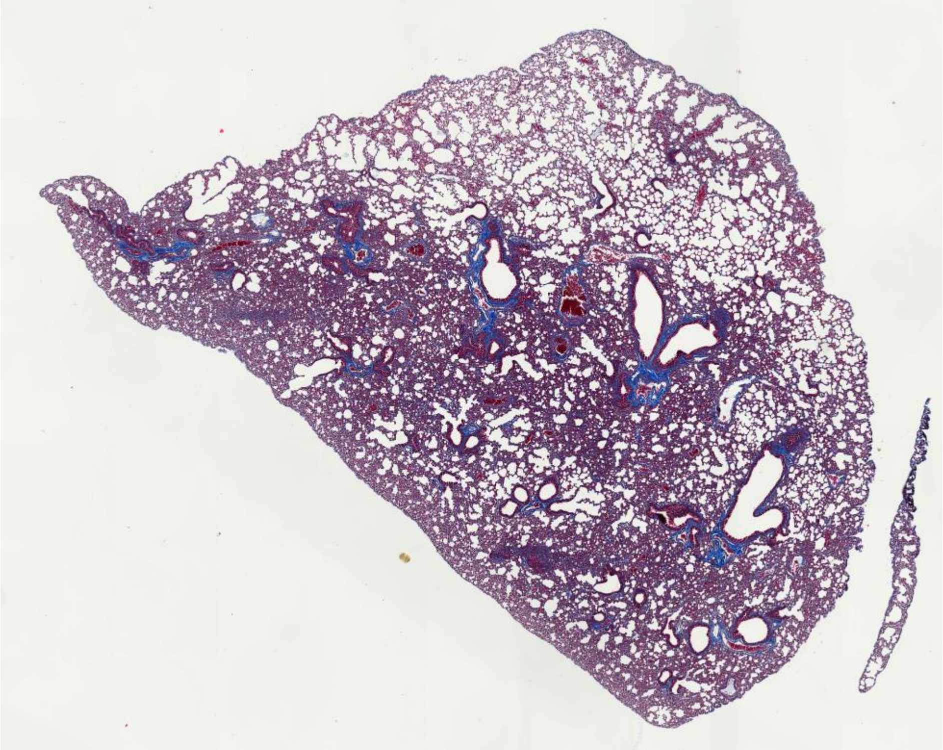

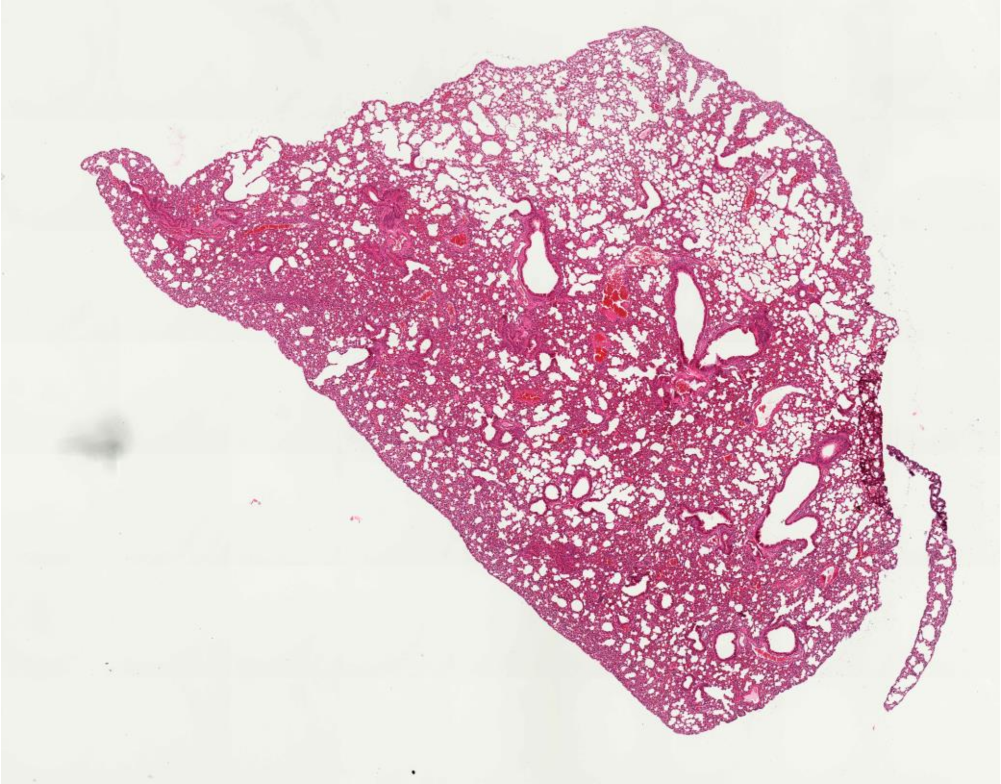

14day-BLM

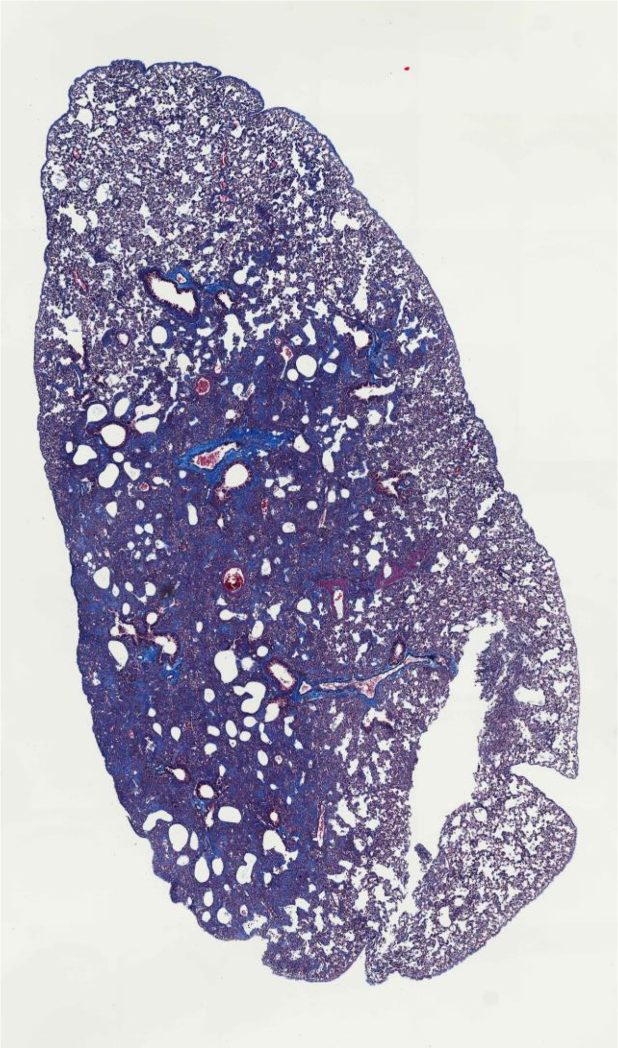

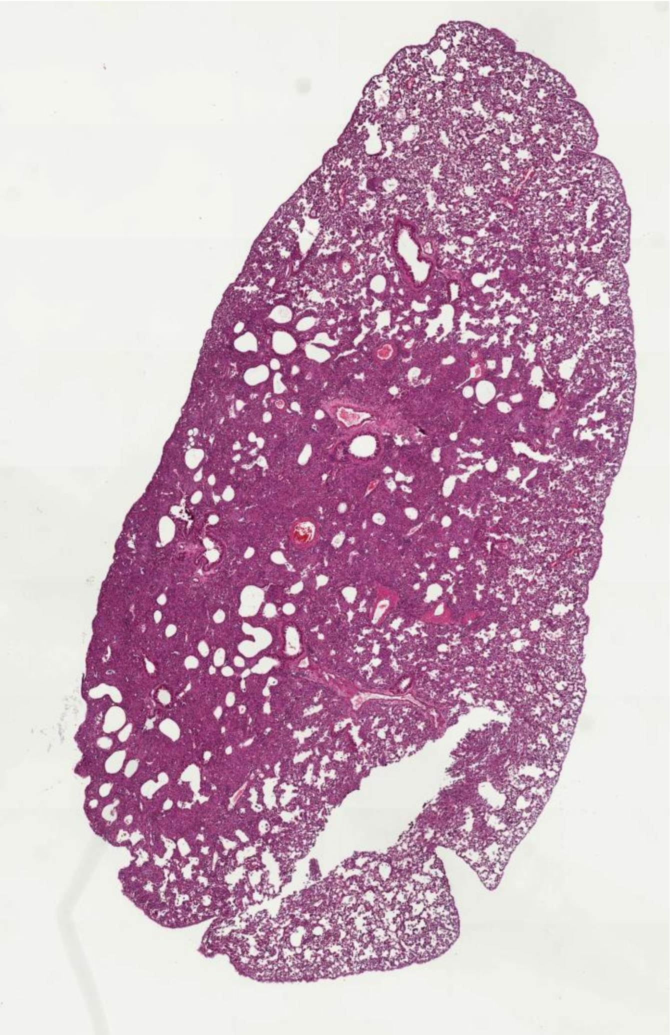

14day-BLM+PFD

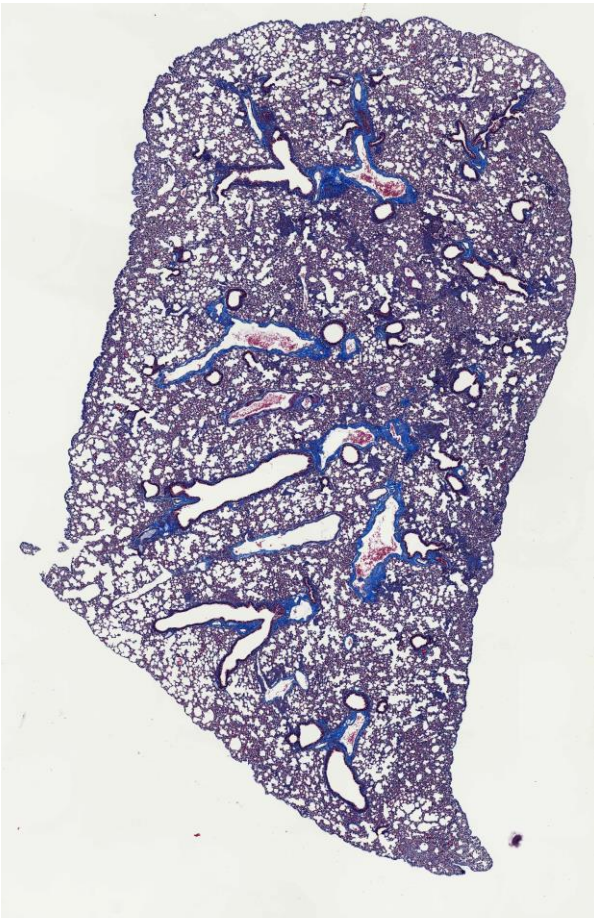

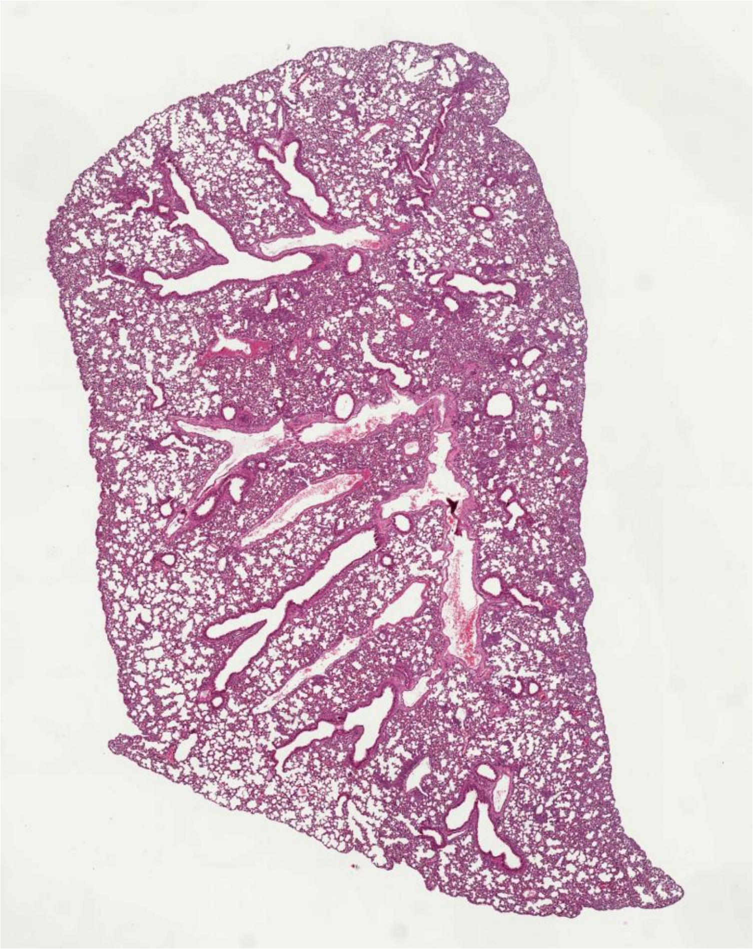

14day-BLM+TEL

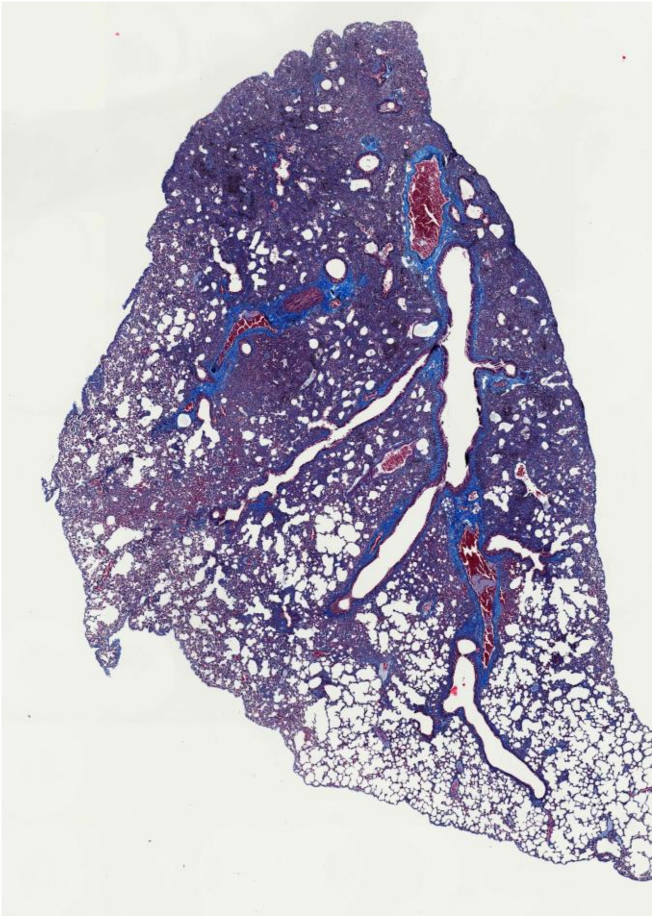

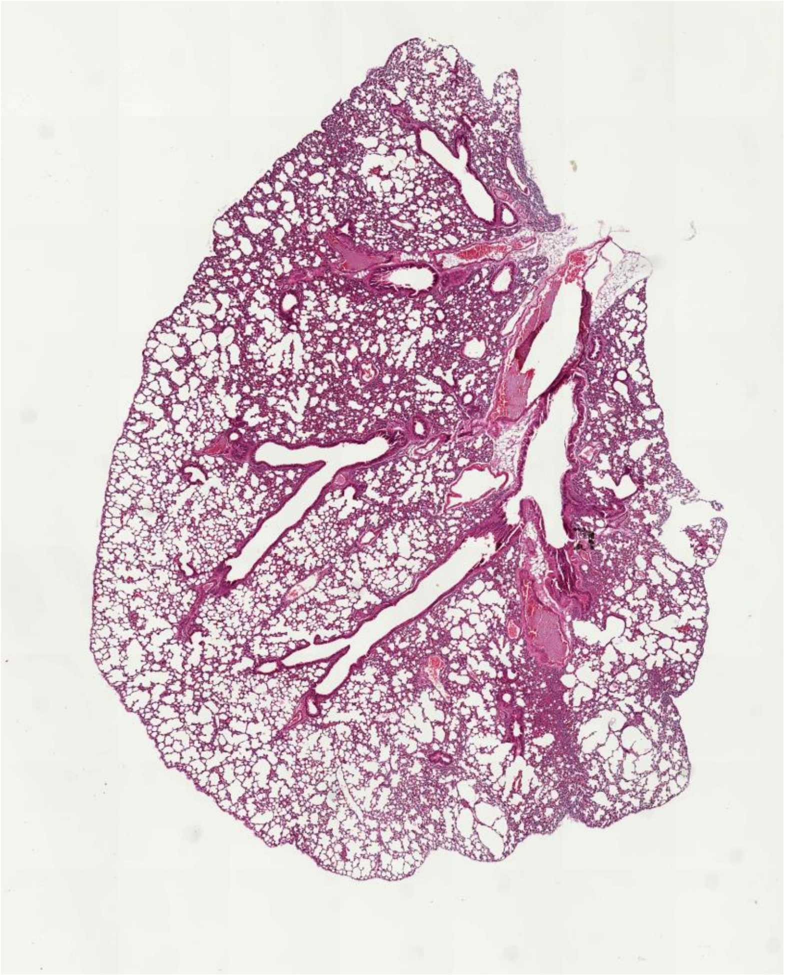

28day-Control

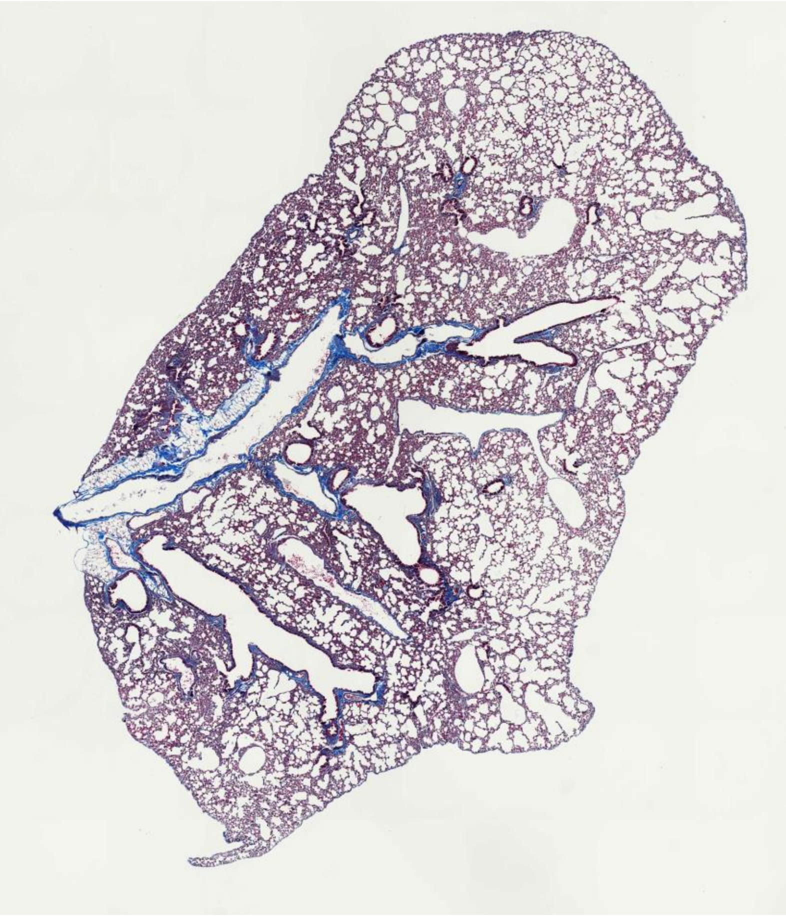

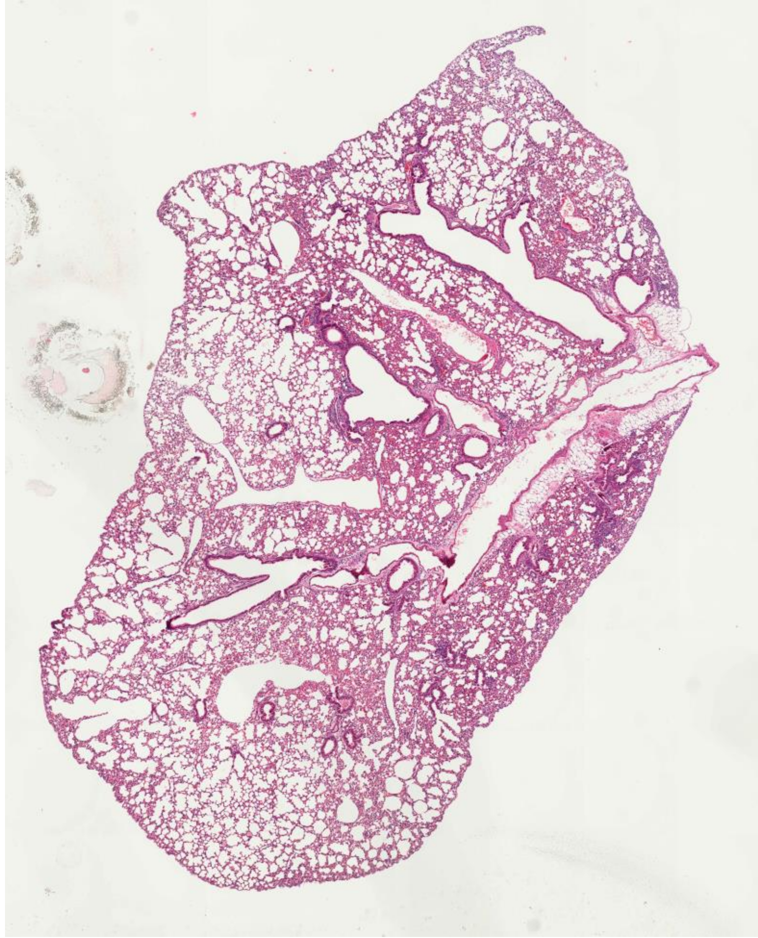

28day-BLM+H CAT

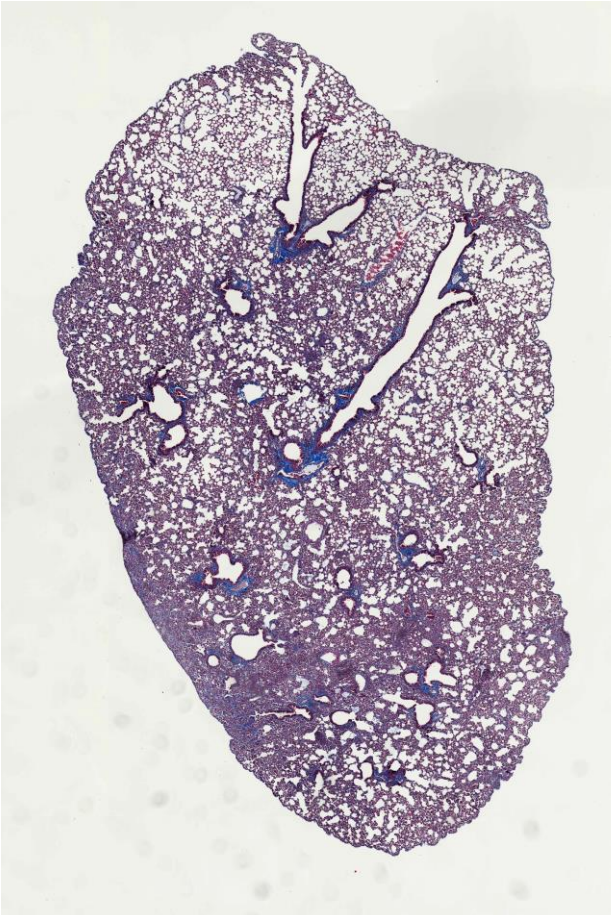

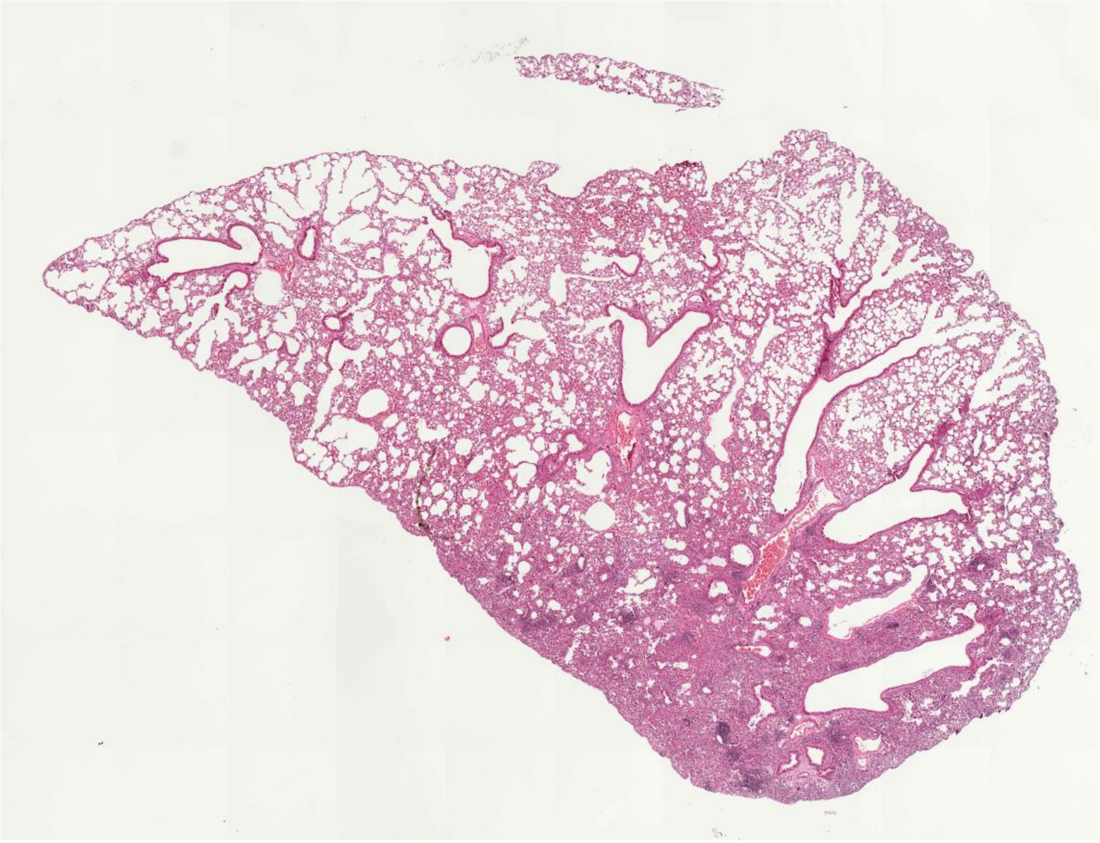

28day-BLM+L CAT

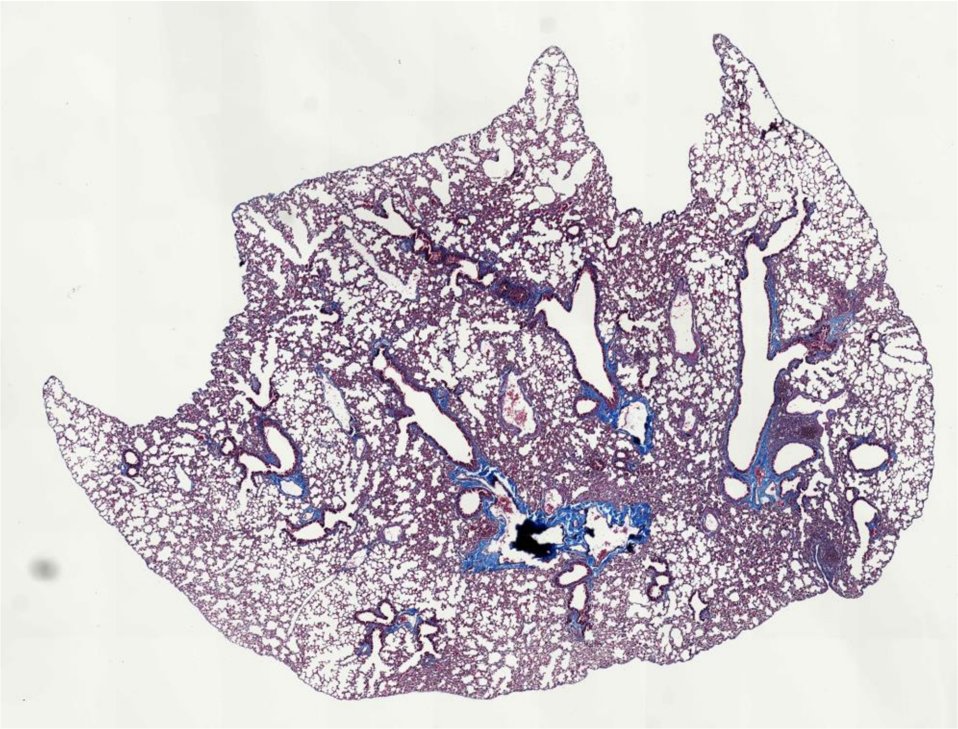

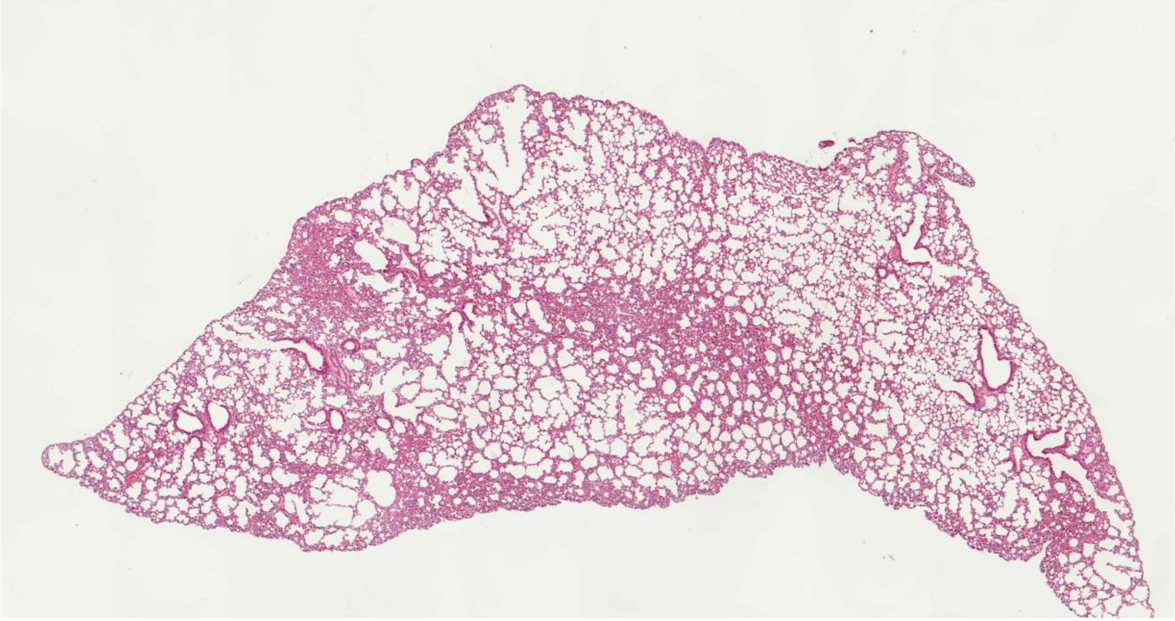

28day-BLM

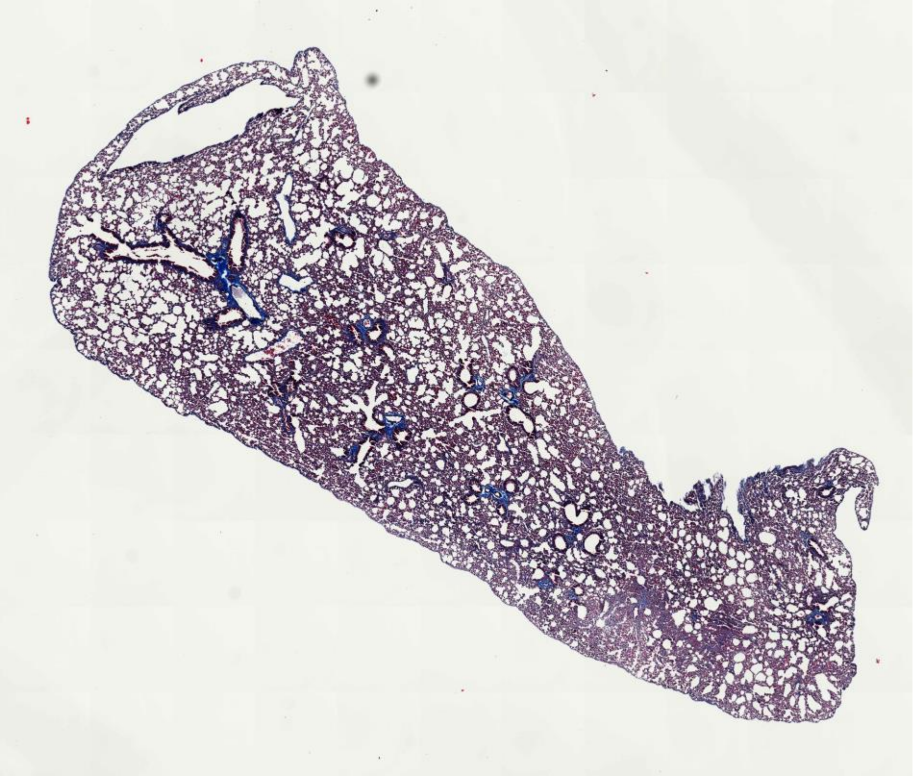

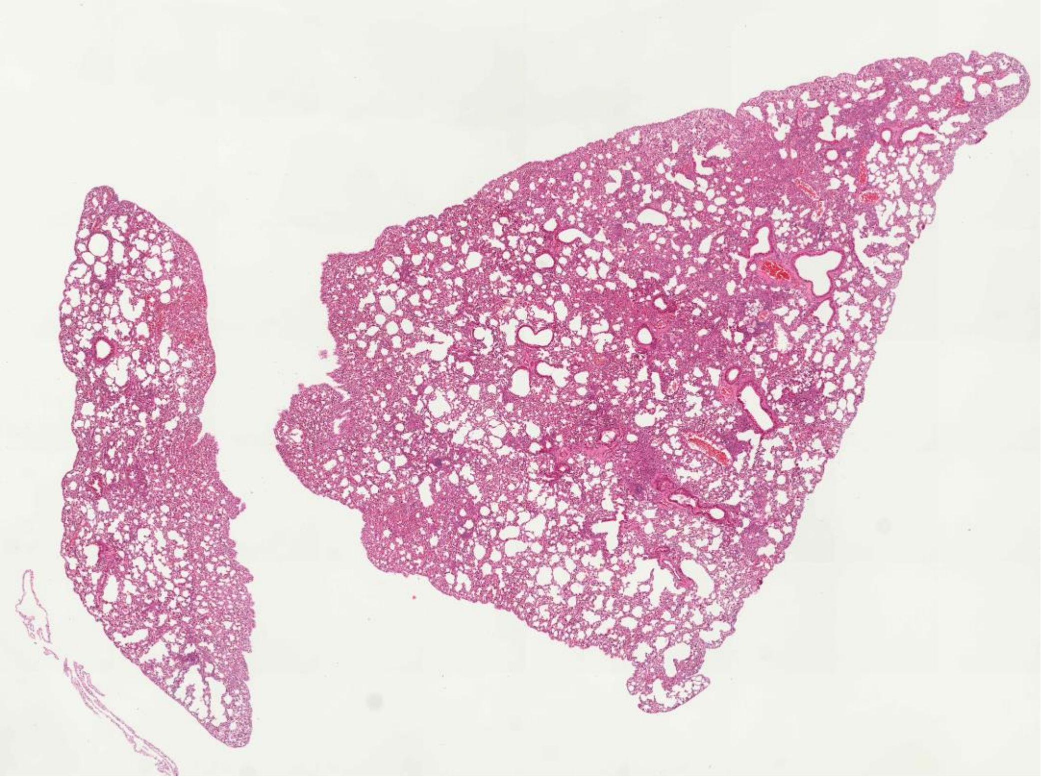

28day-BLM+PFD

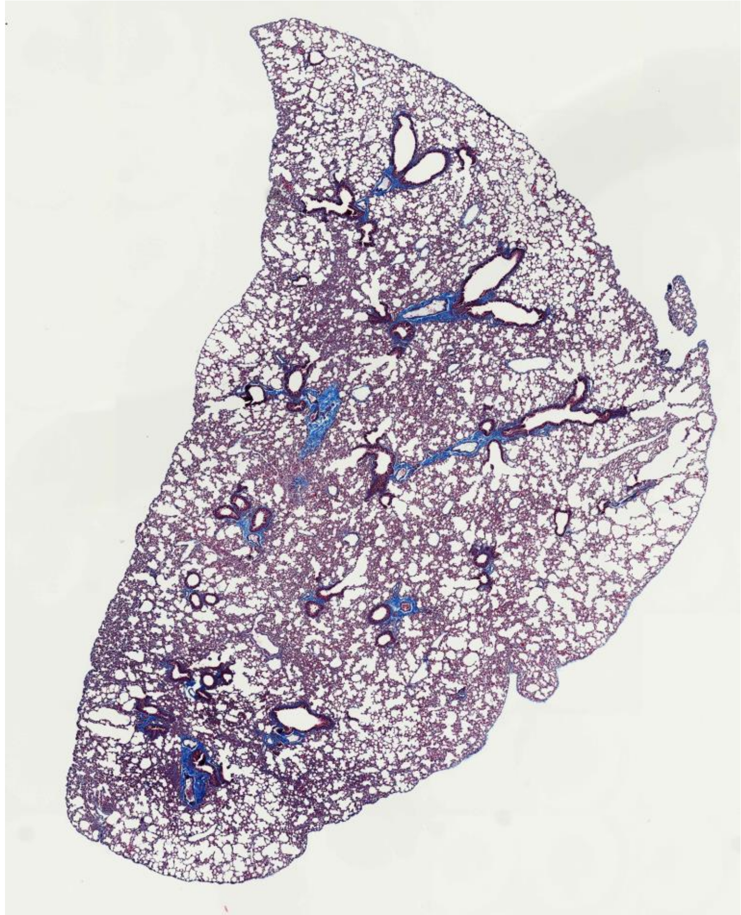

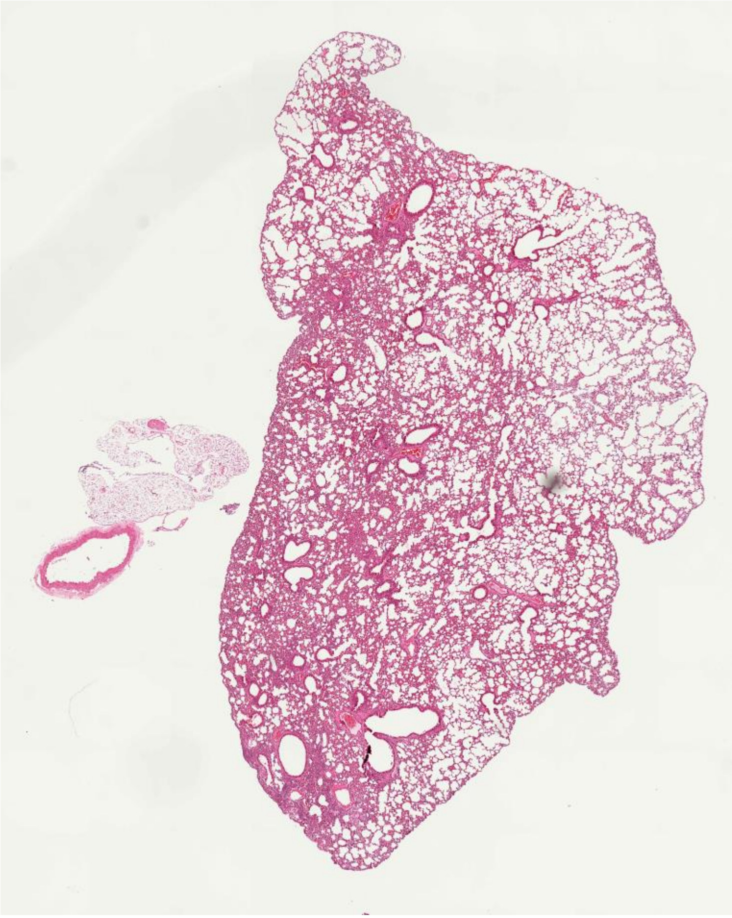

28day-BLM+TEL

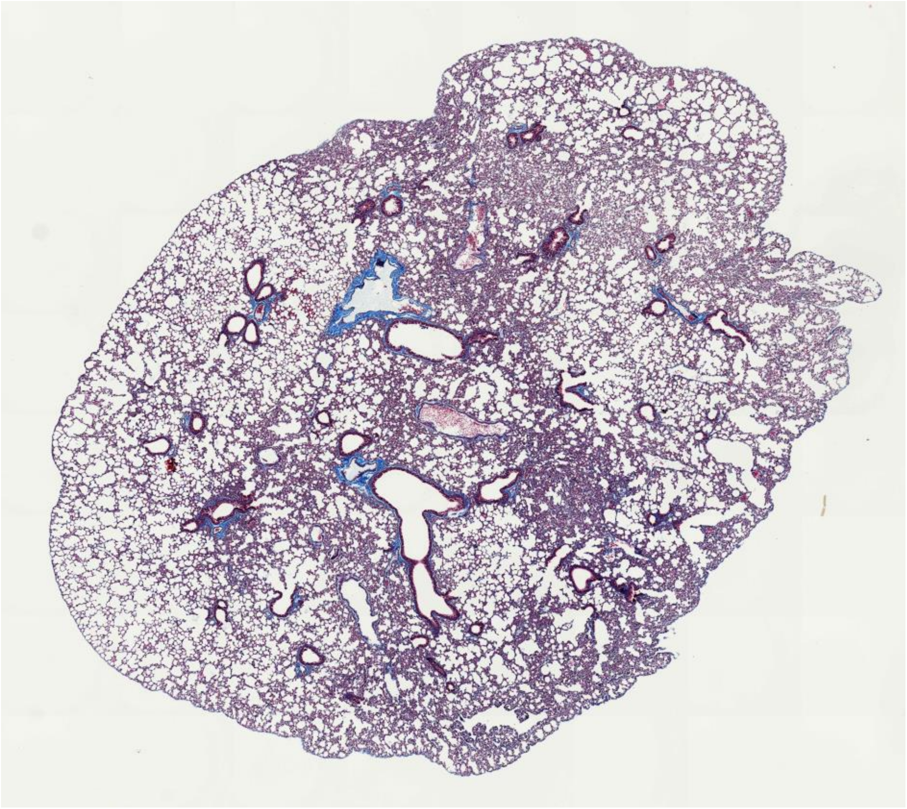

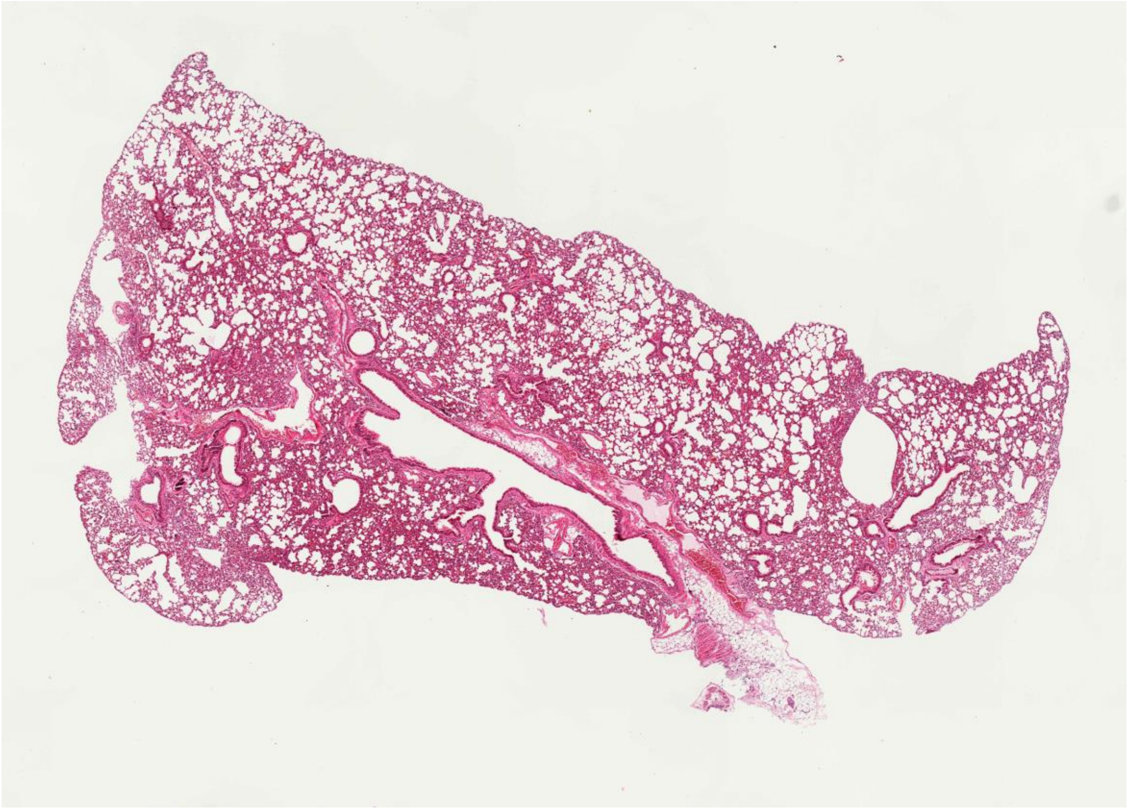

Supplement: Supplementary file 3 [file Data_Sheet_1.PDF]
