# Supplementary material for: Catalpol Attenuates Pulmonary Fibrosis by Inhibiting Ang II/AT1 and TGF-β/Smad-Mediated Epithelial Mesenchymal Transition
Source: Front Med (Lausanne). 2022 May 24;9:878601. doi: 10.3389/fmed.2022.878601 (PMC9171363; doi:10.3389/fmed.2022.878601)

α-SMA

7day-Control

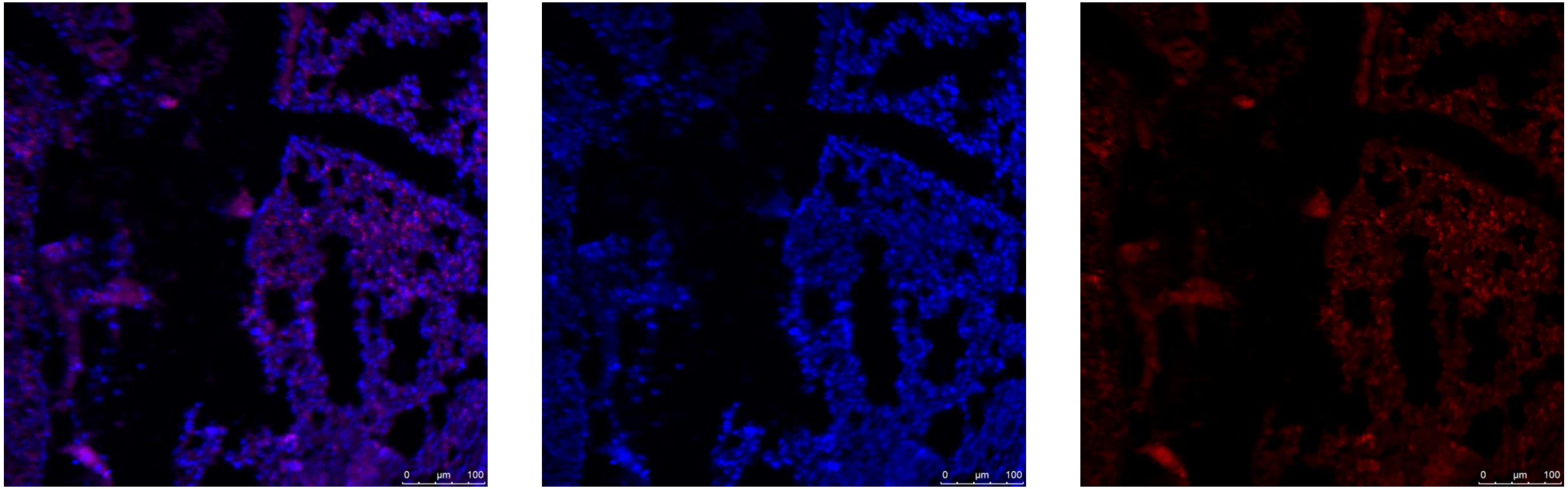

7day-BLM+H CAT

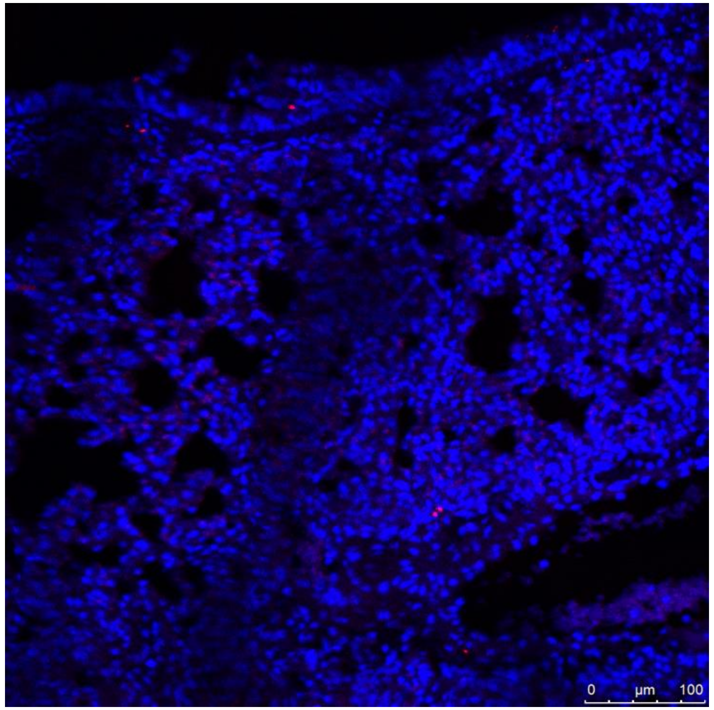

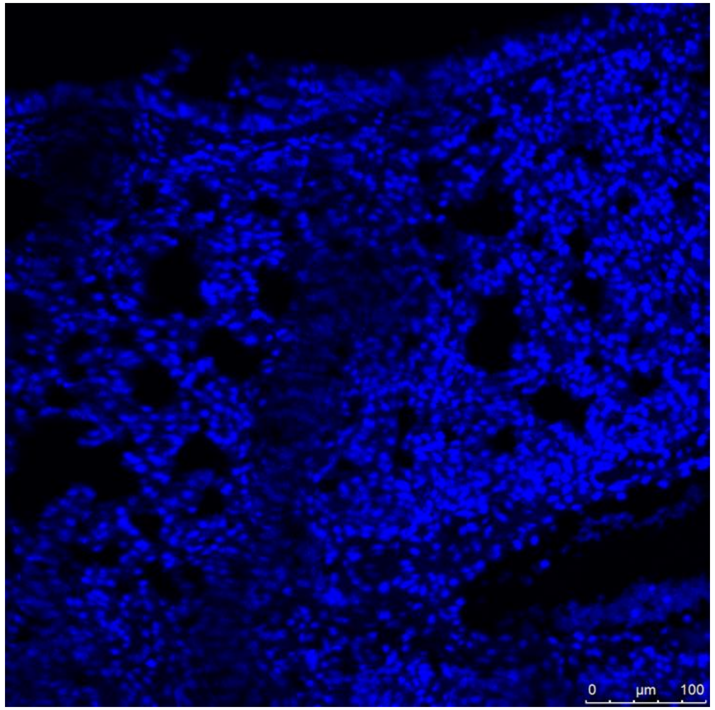

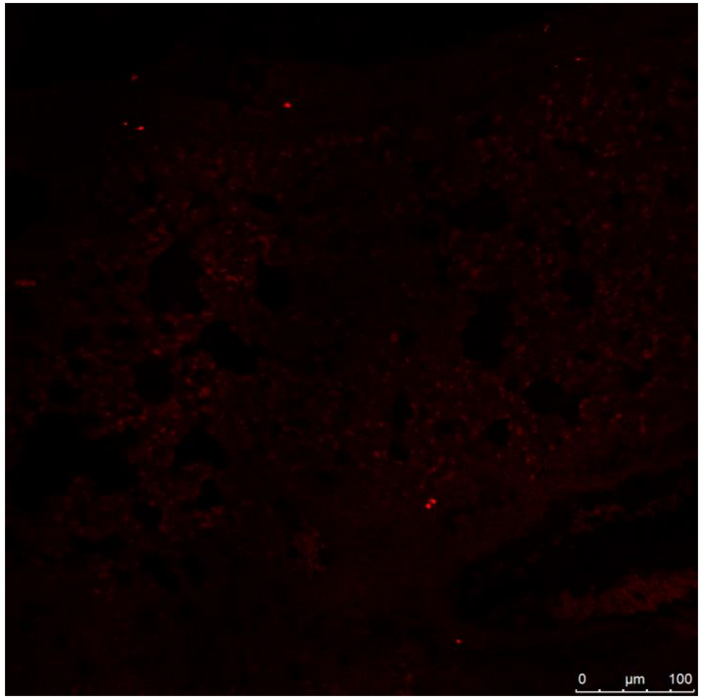

7day-BLM+L CAT

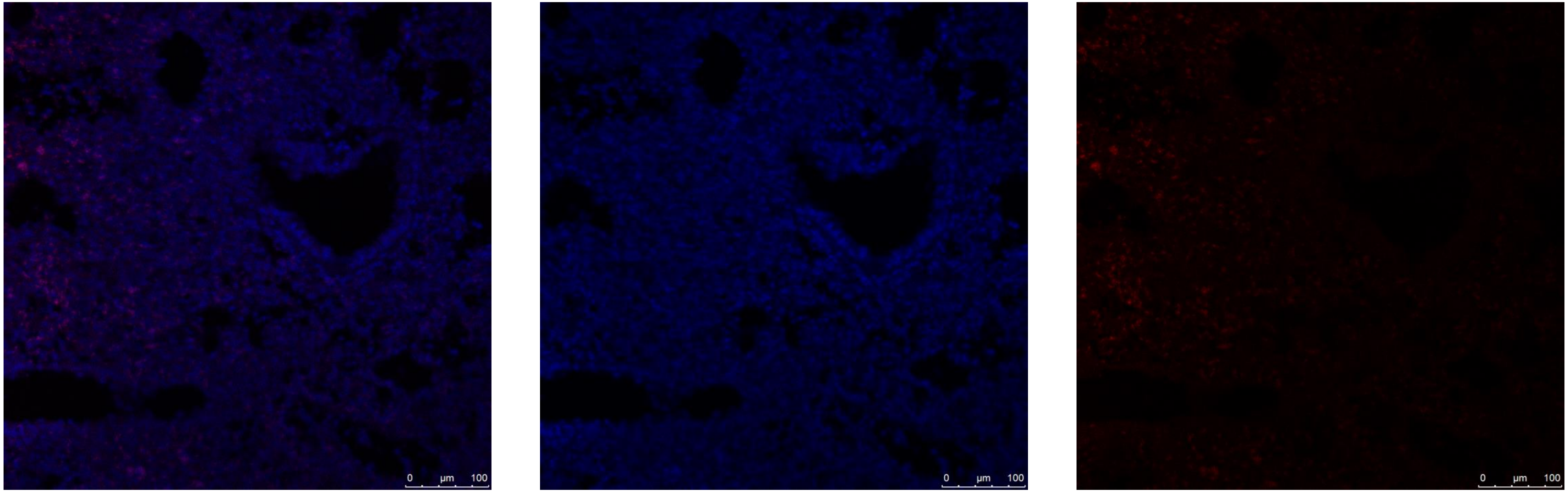

7day-BLM

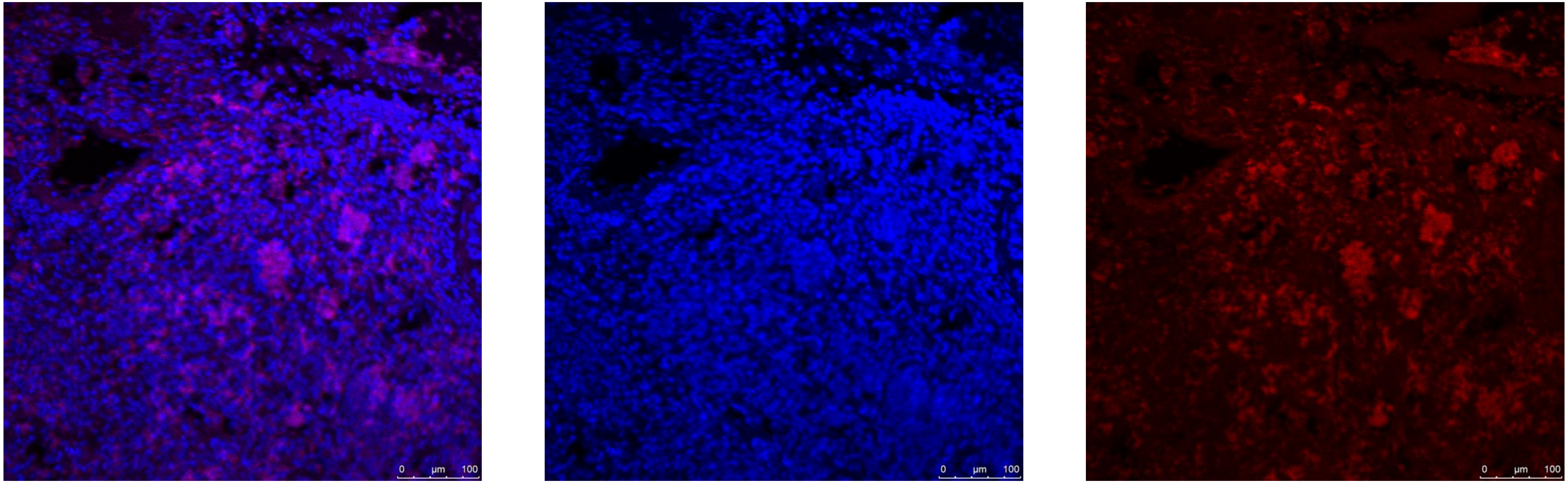

7day-BLM+PFD

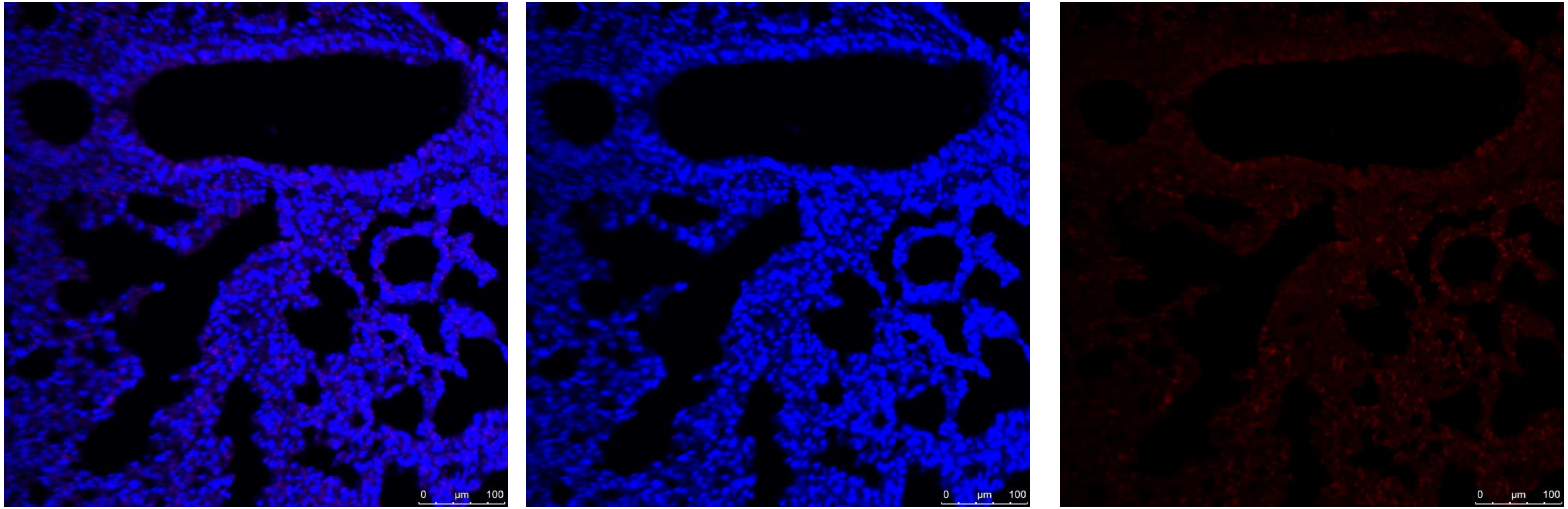

7day-BLM+TEL

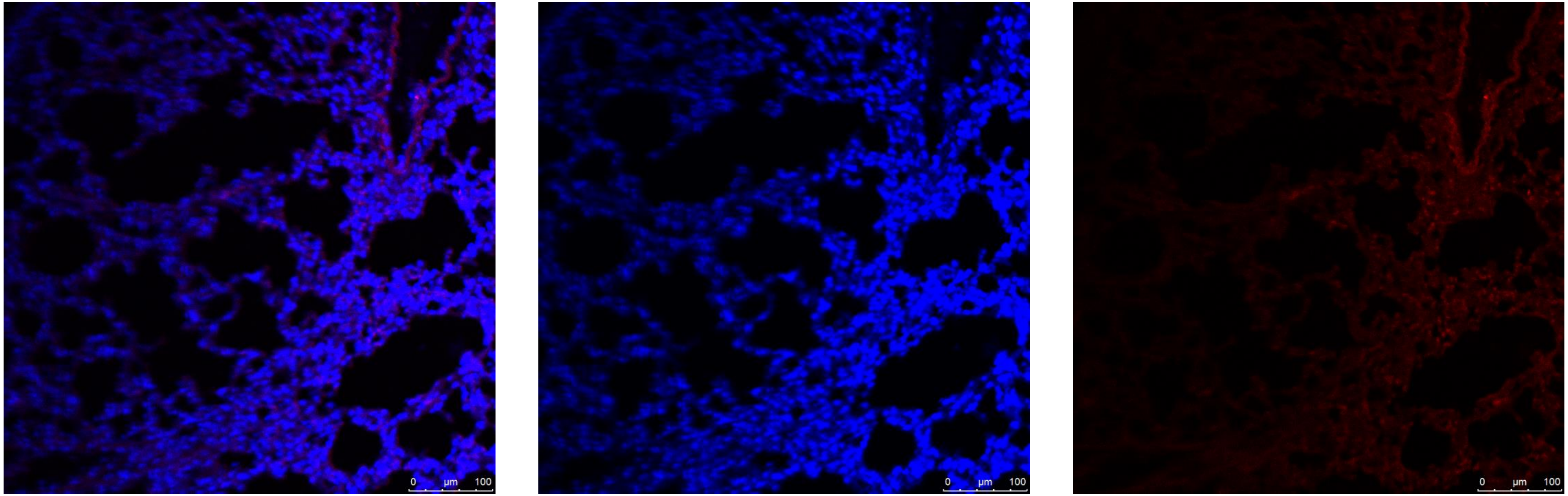

14day-Control

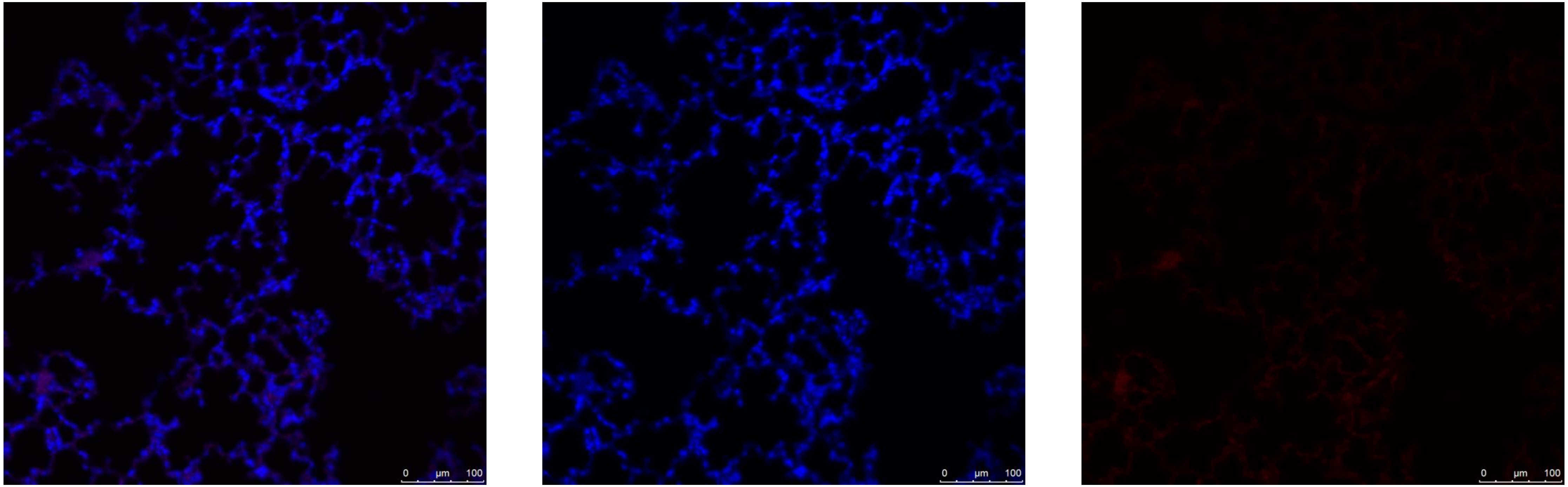

14day-BLM+H CAT

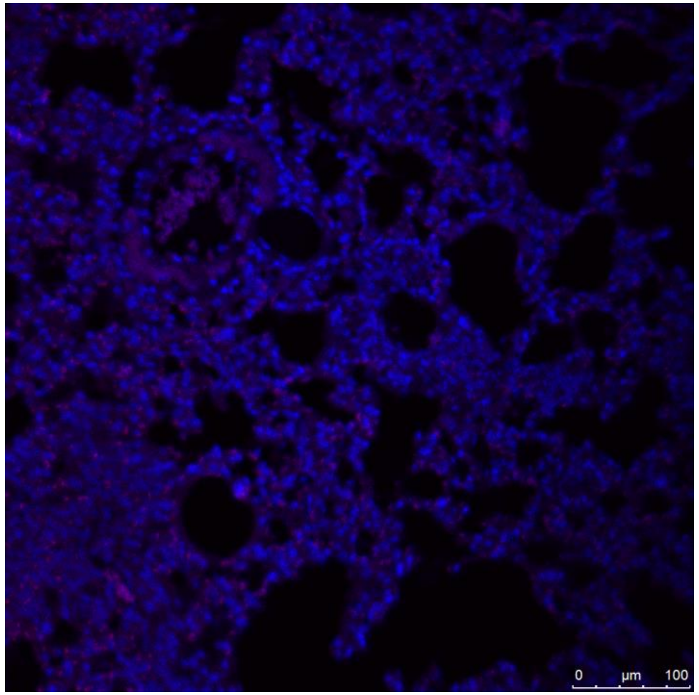

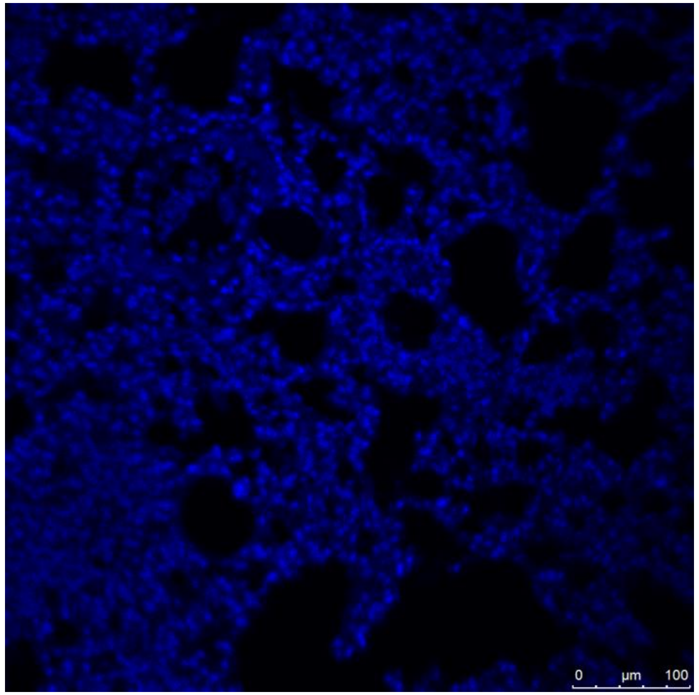

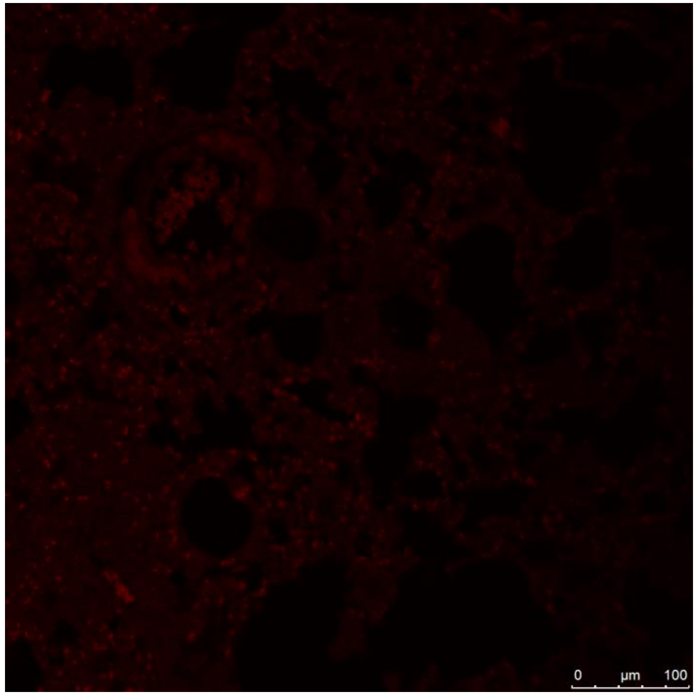

14day-BLM+L CAT

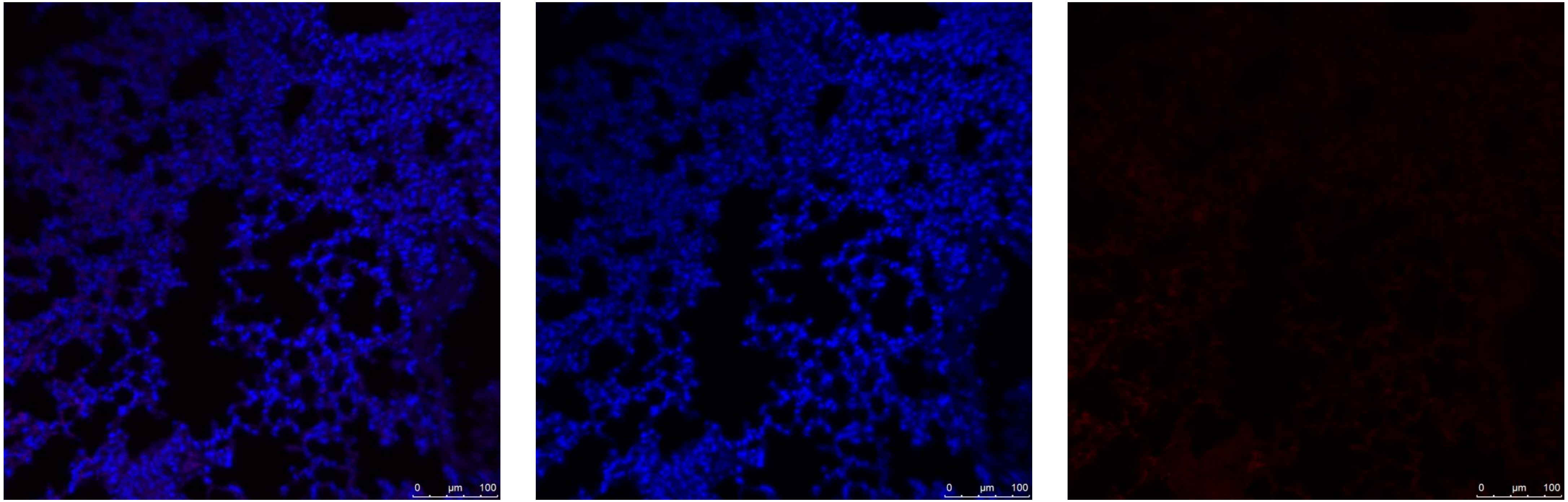

14day-BLM

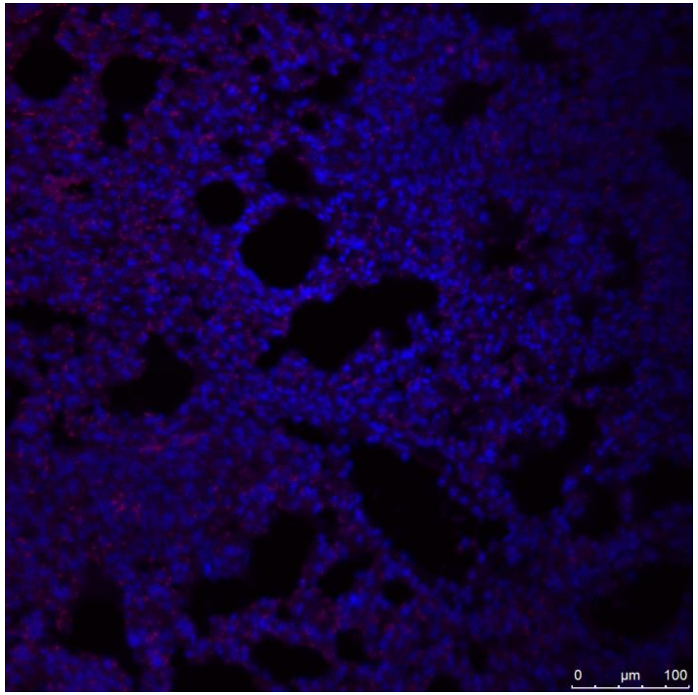

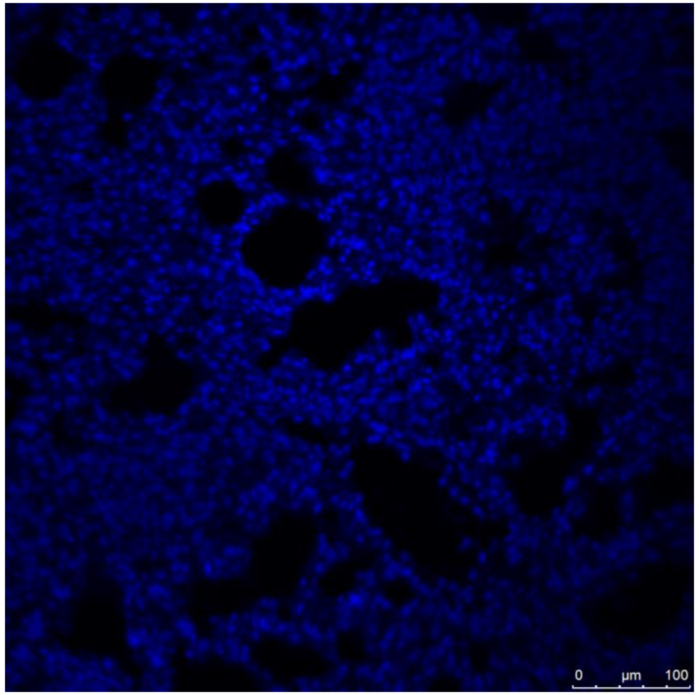

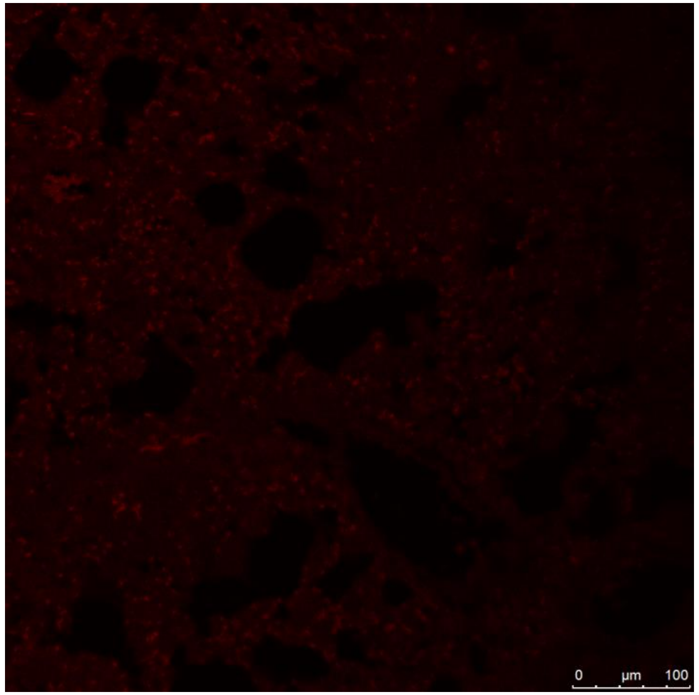

14day-BLM+PFD

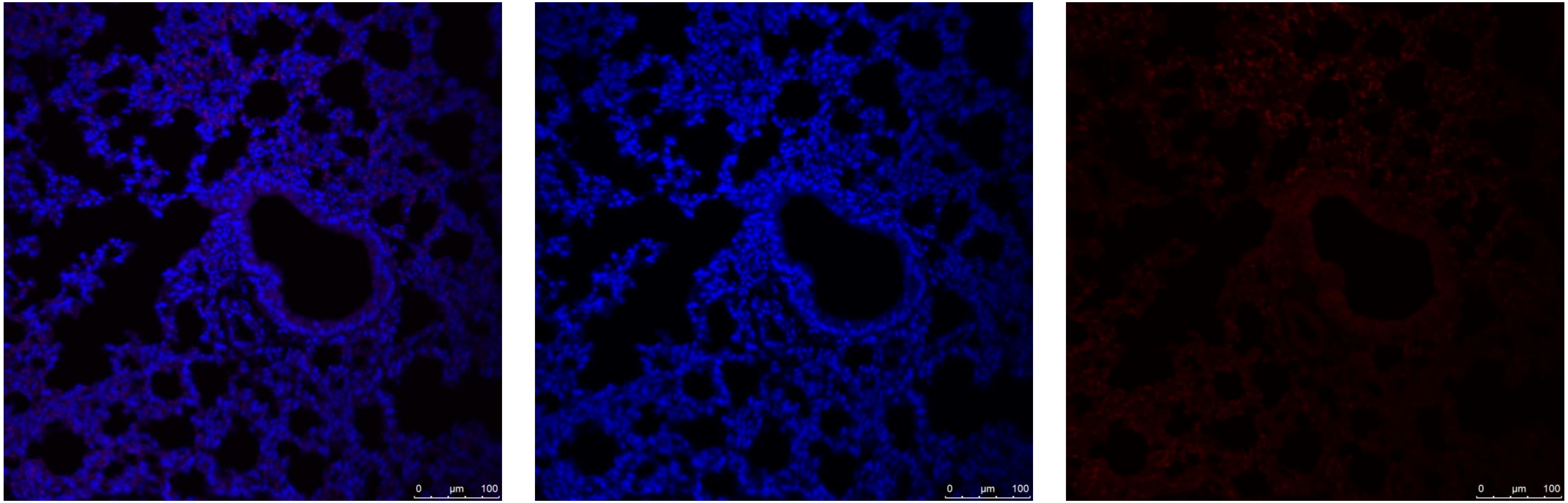

14day-BLM+TEL

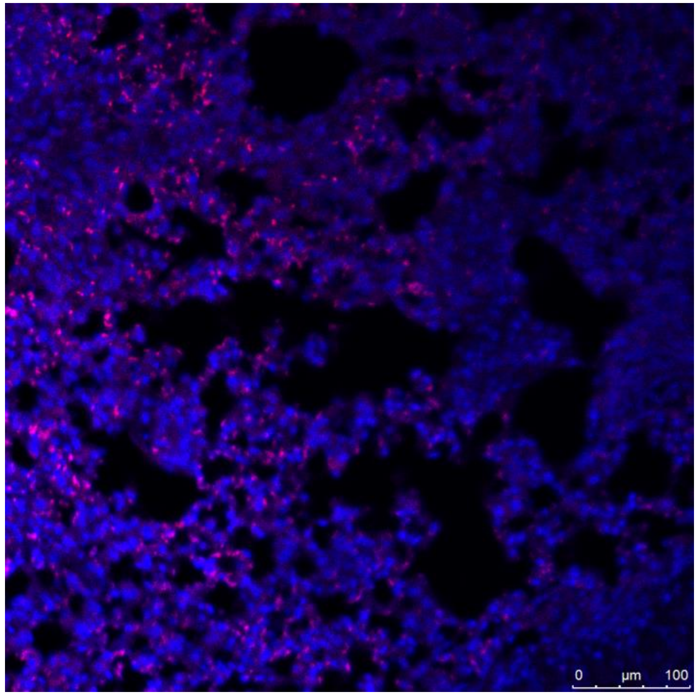

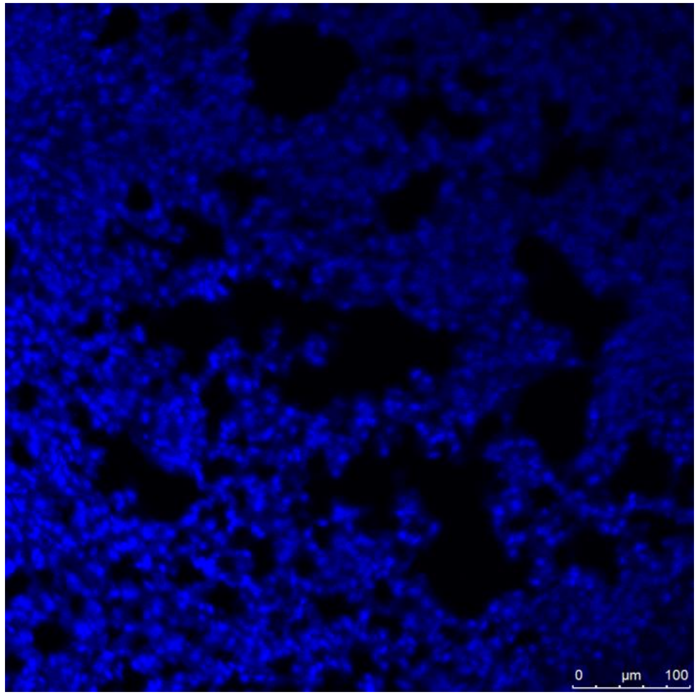

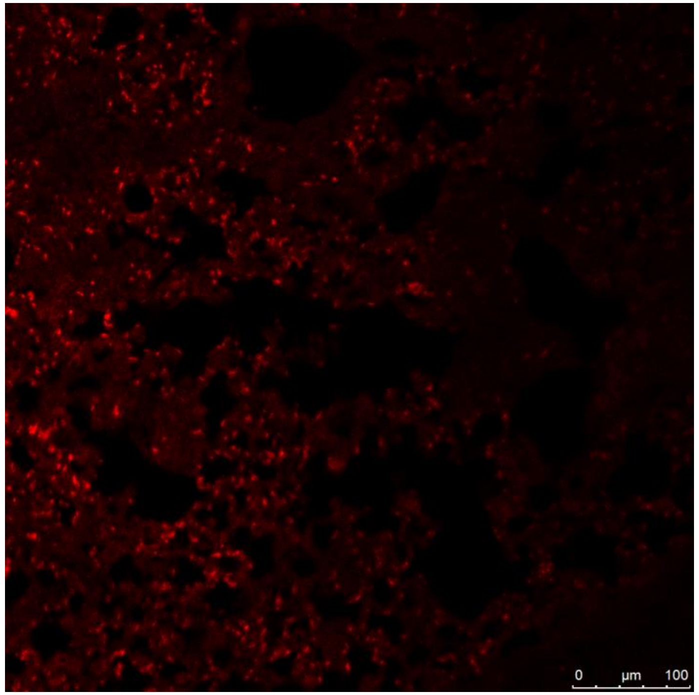

28day-Control

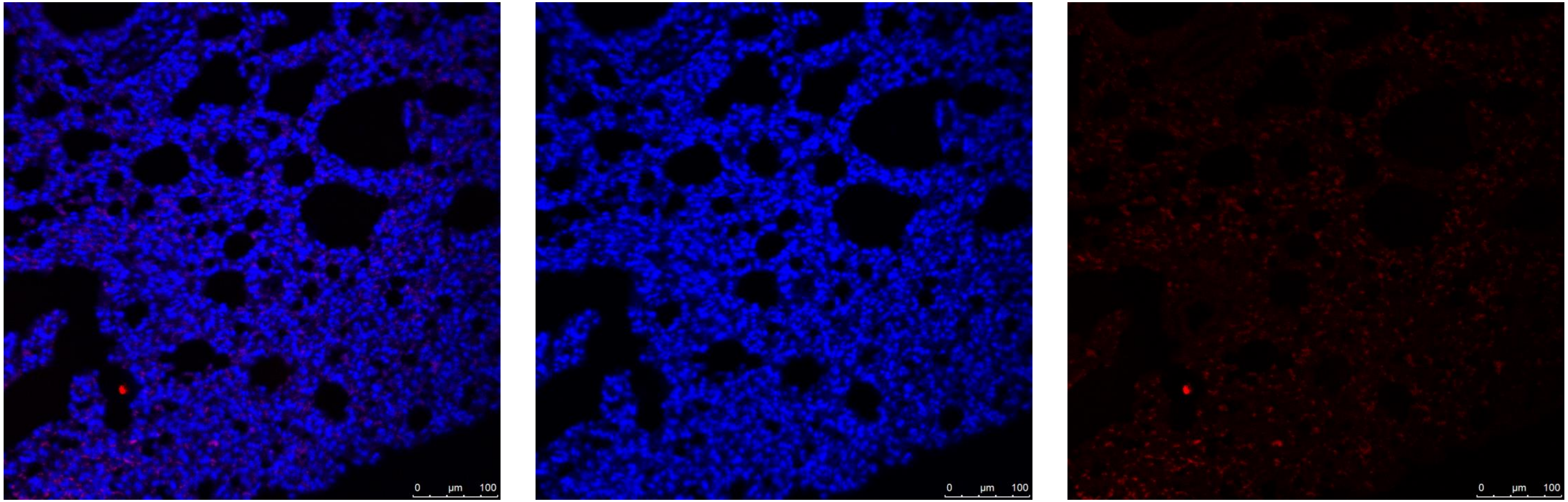

28day-BLM+H CAT

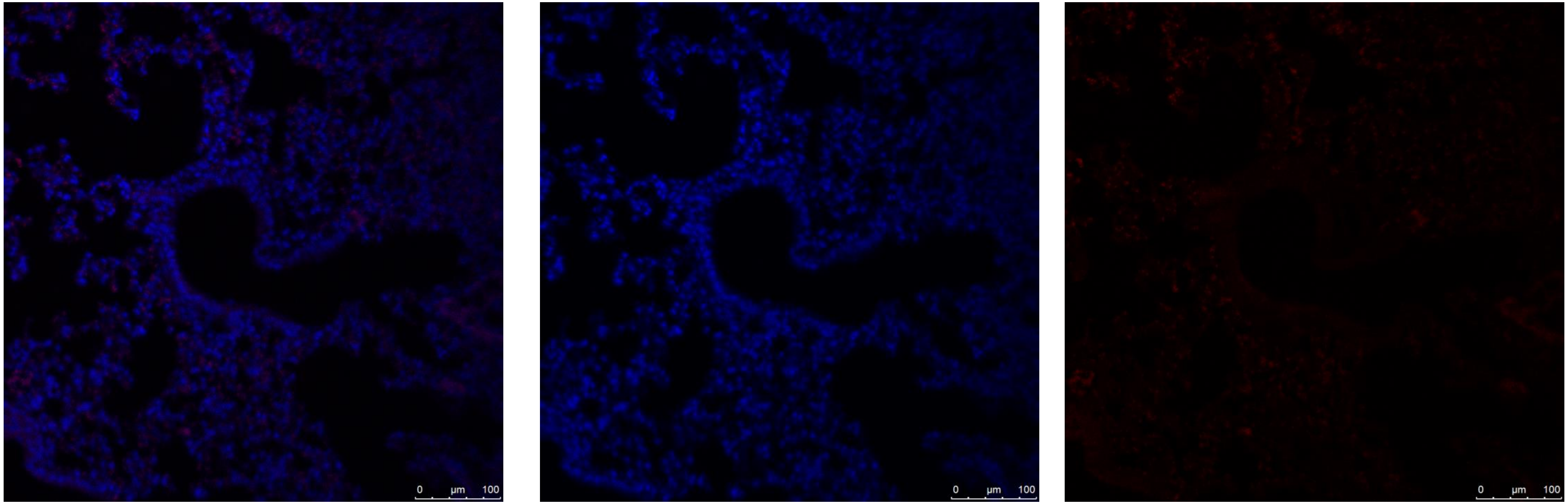

28day-BLM+L CAT

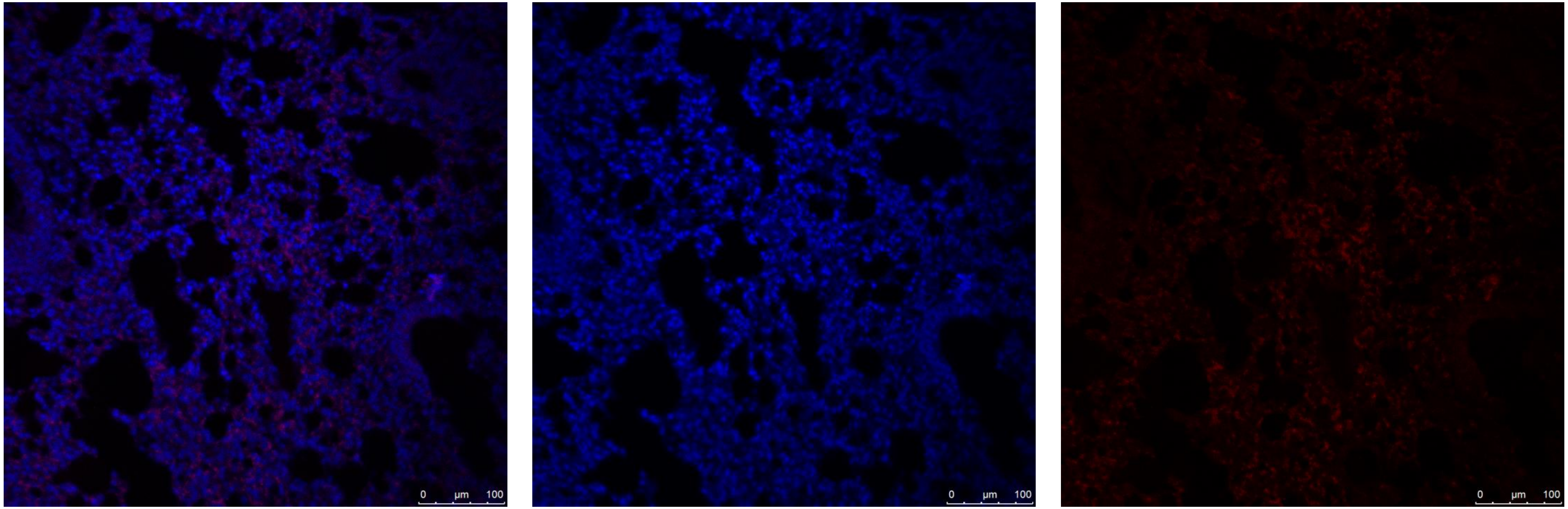

28day-BLM

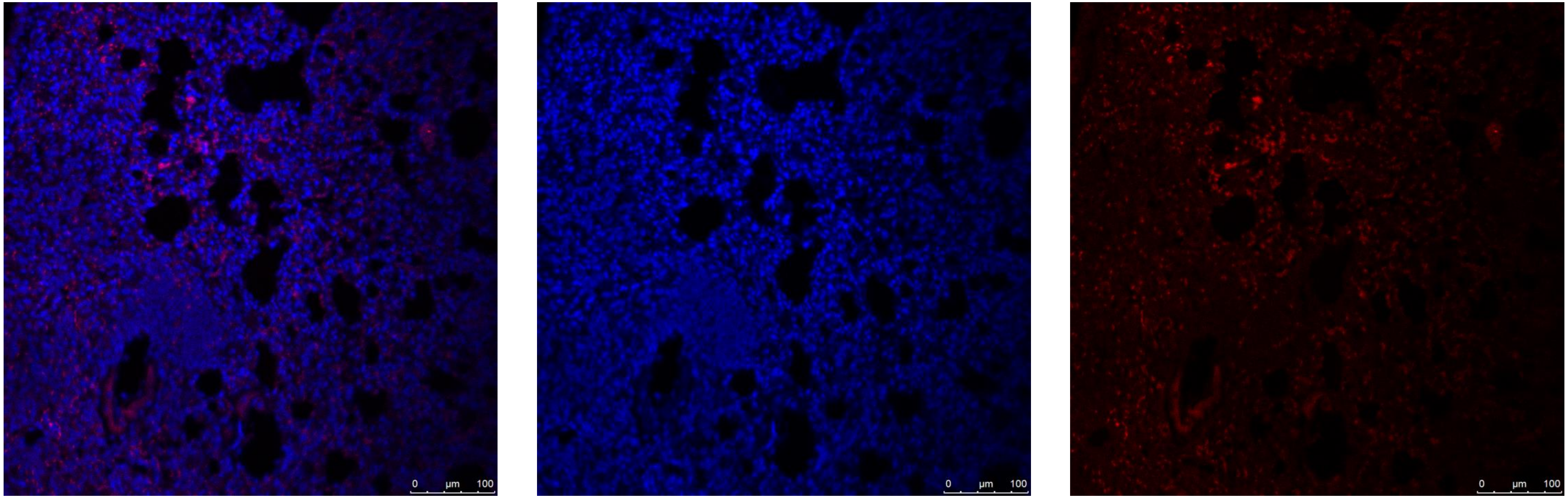

28day-BLM+PFD

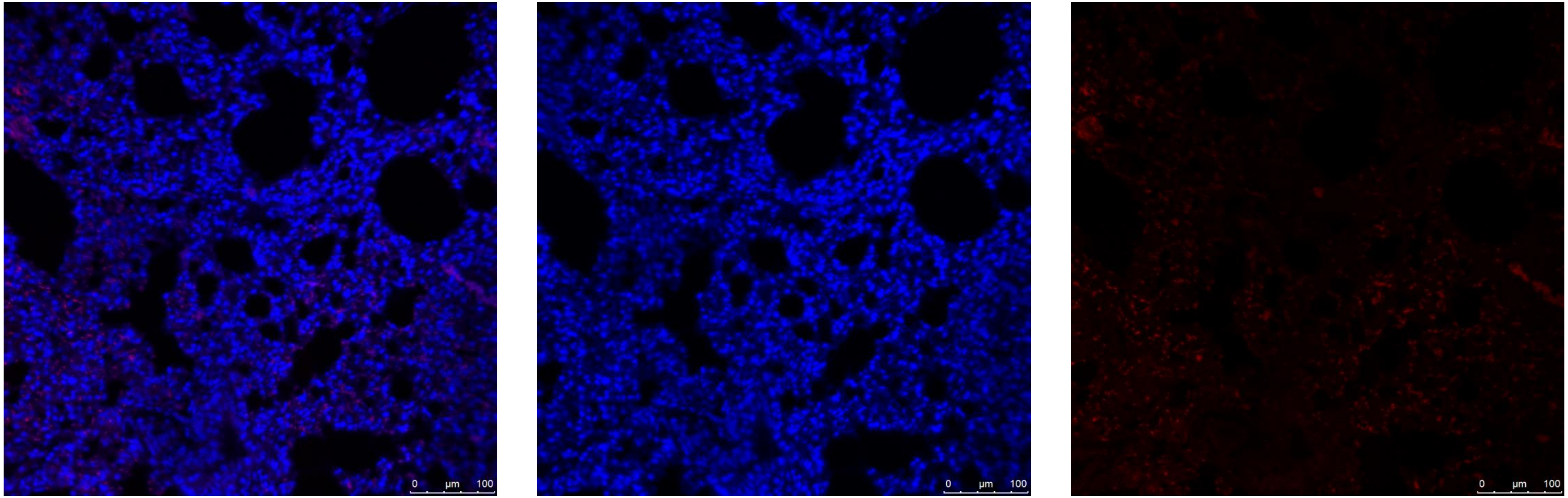

28day-BLM+TEL

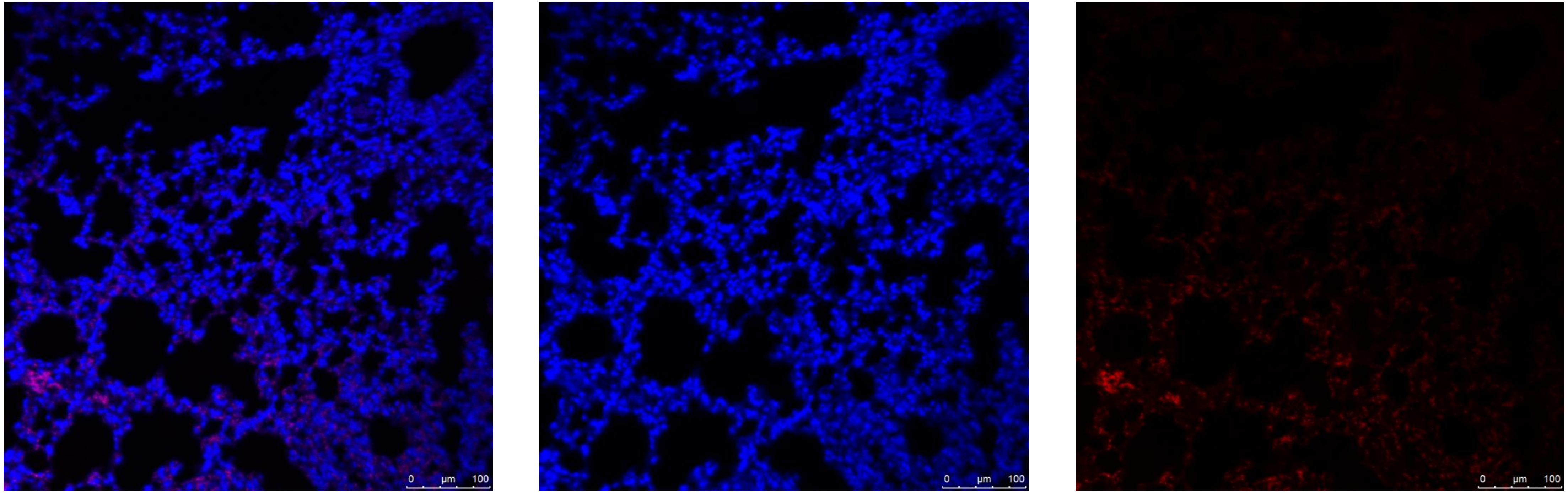

N-cadherin

7day-Control

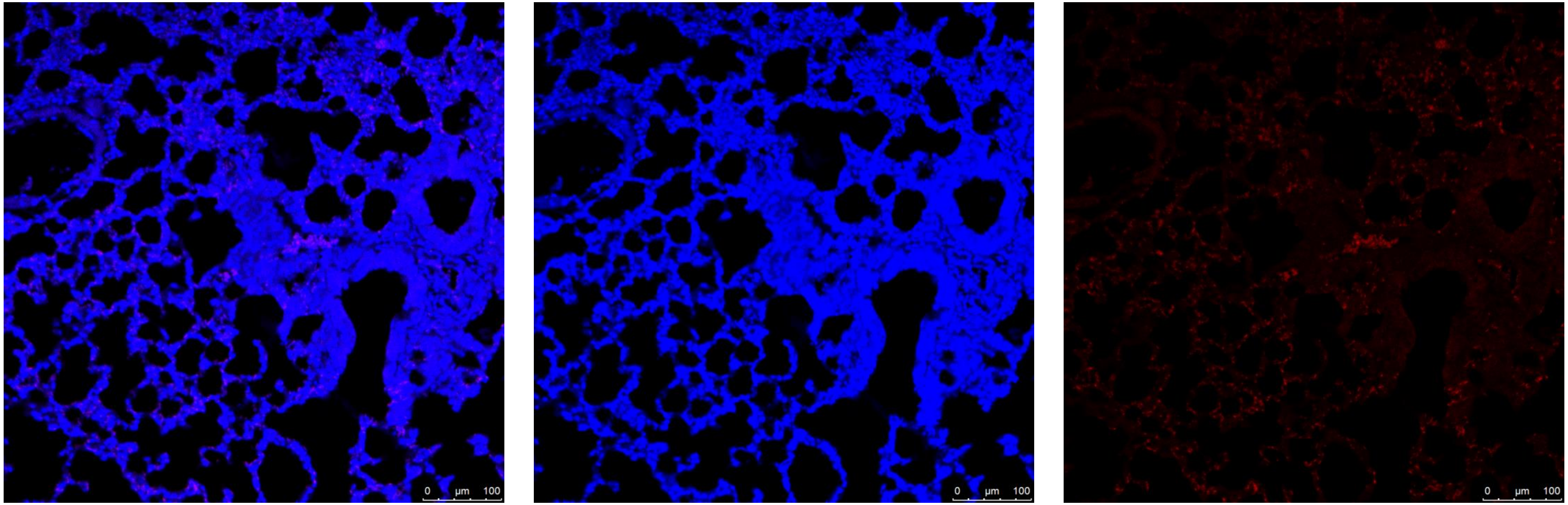

7day-BLM+H CAT

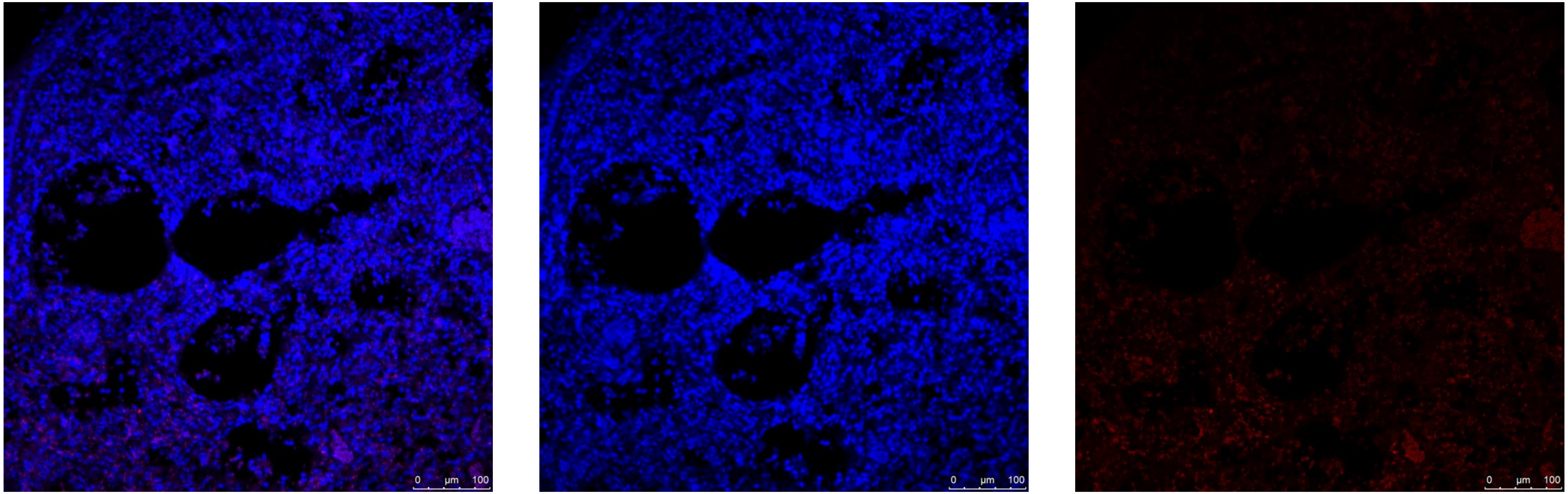

7day-BLM+L CAT

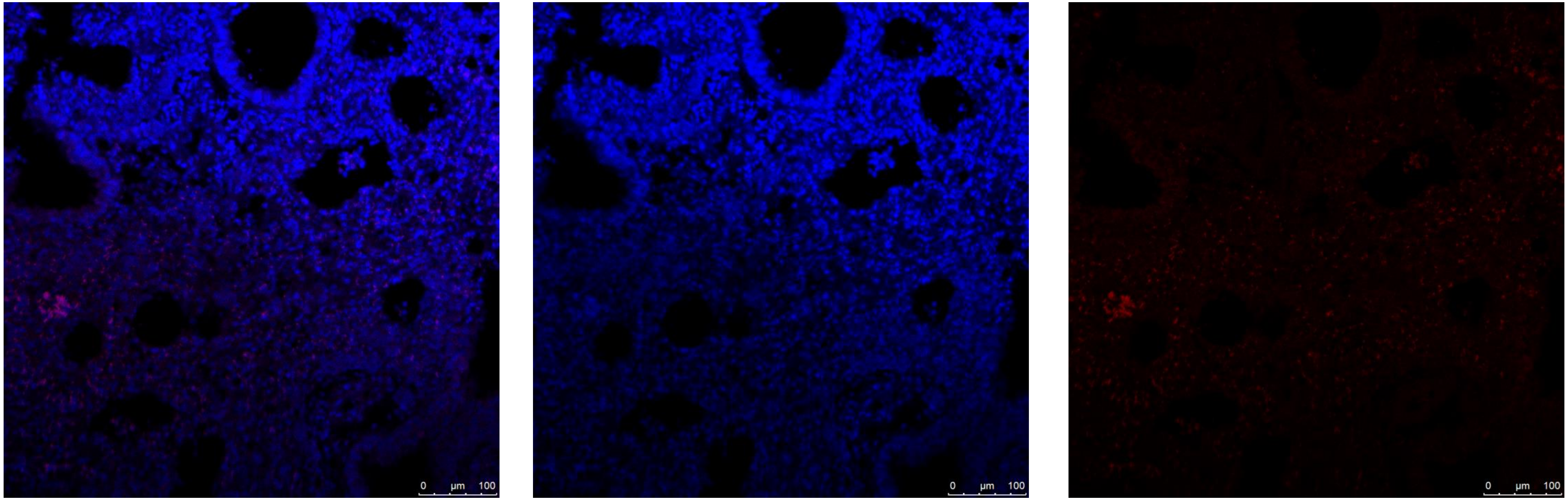

7day-BLM

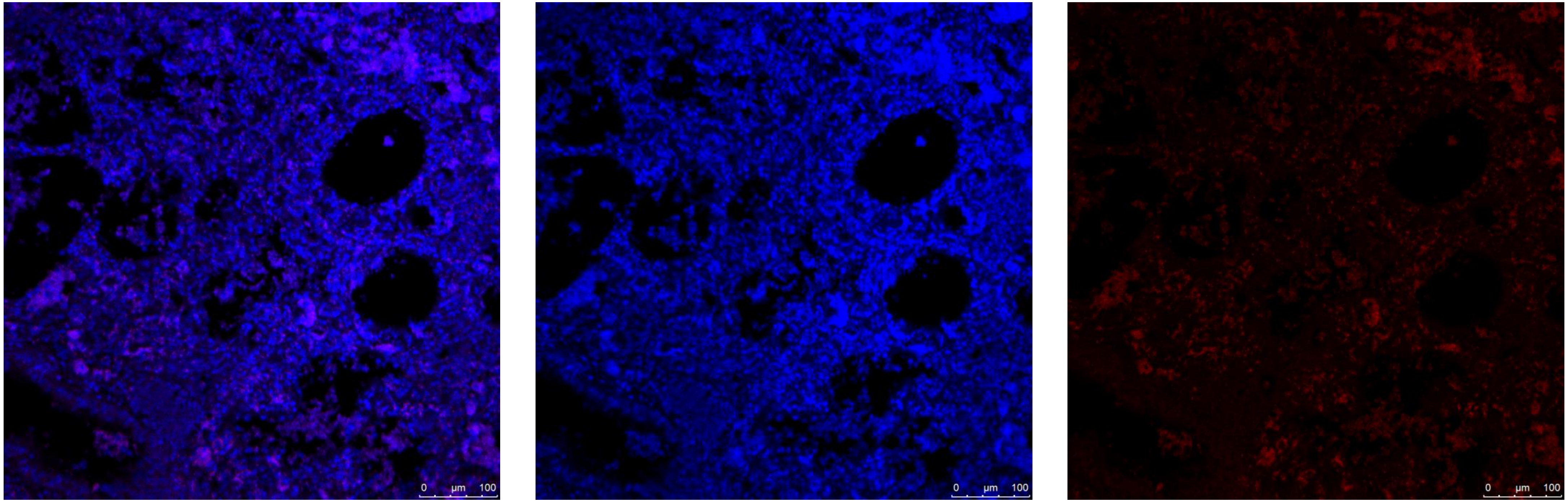

7day-BLM+PFD

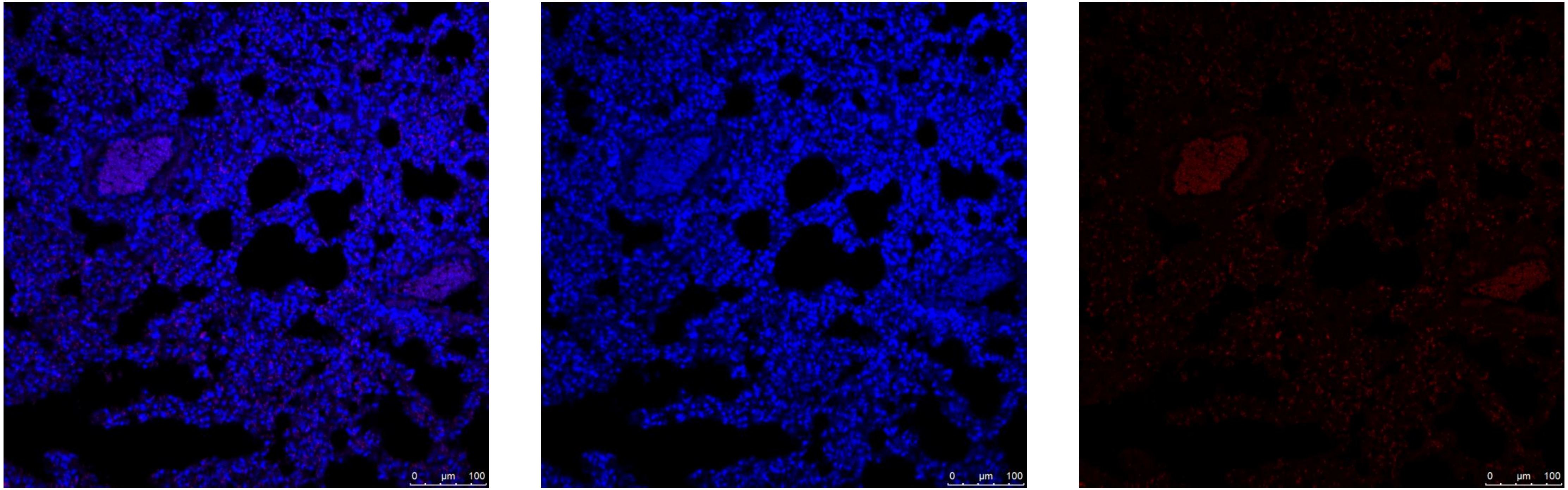

7day-BLM+TEL

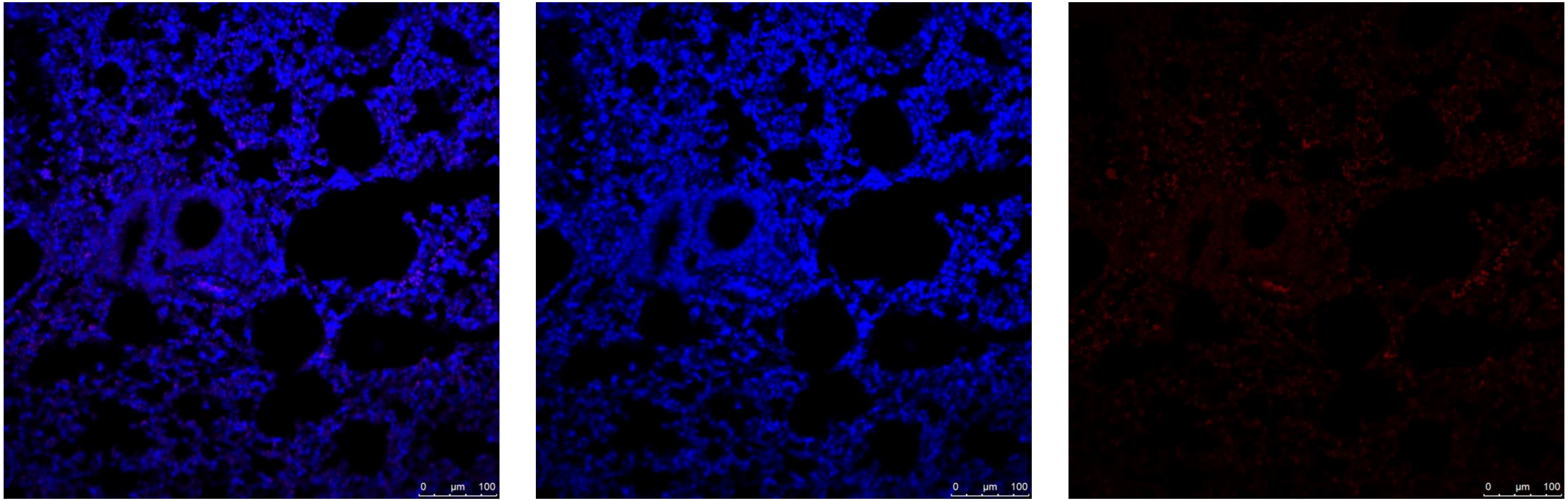

14day-Control

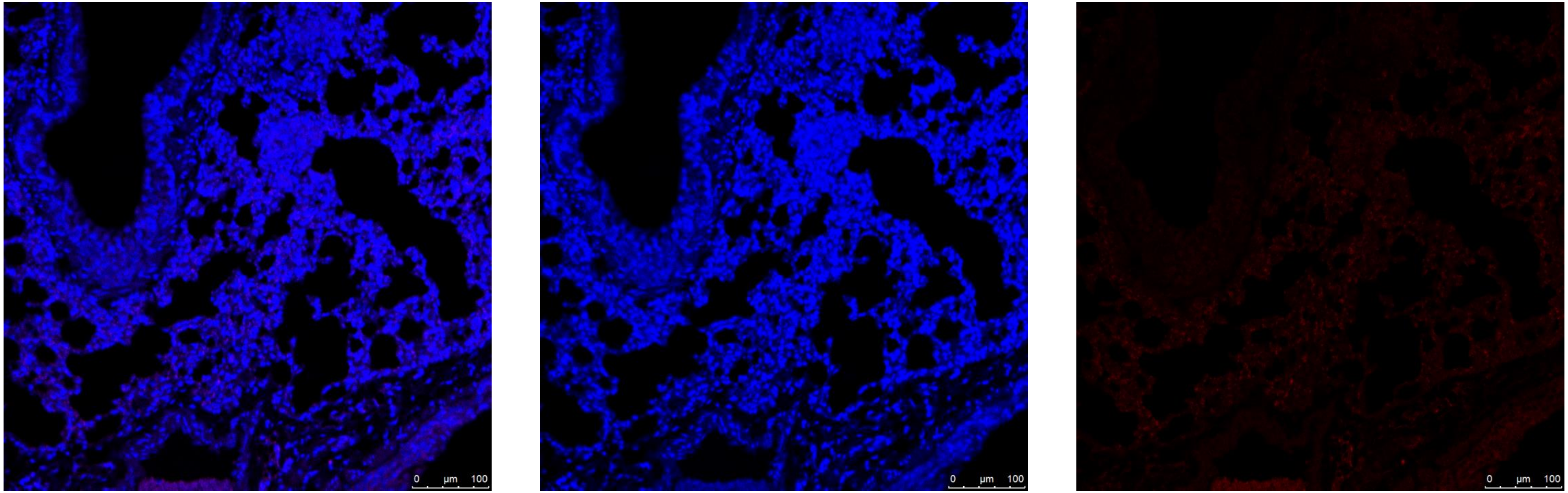

14day-BLM+H CAT

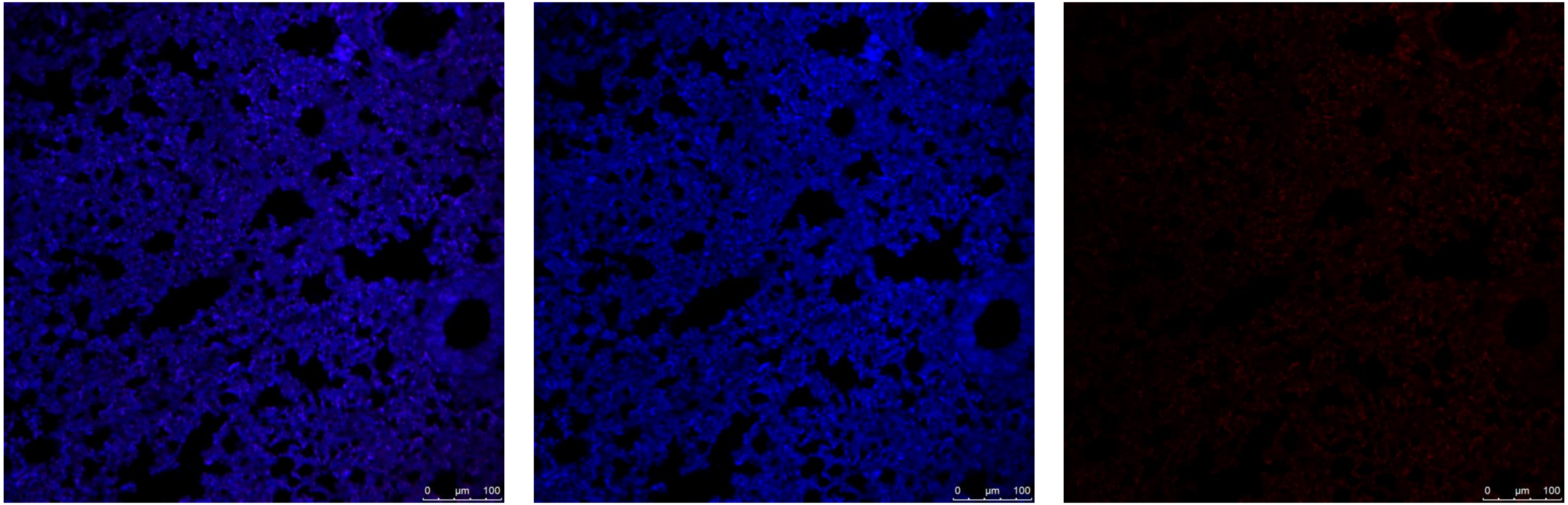

14day-BLM+L CAT

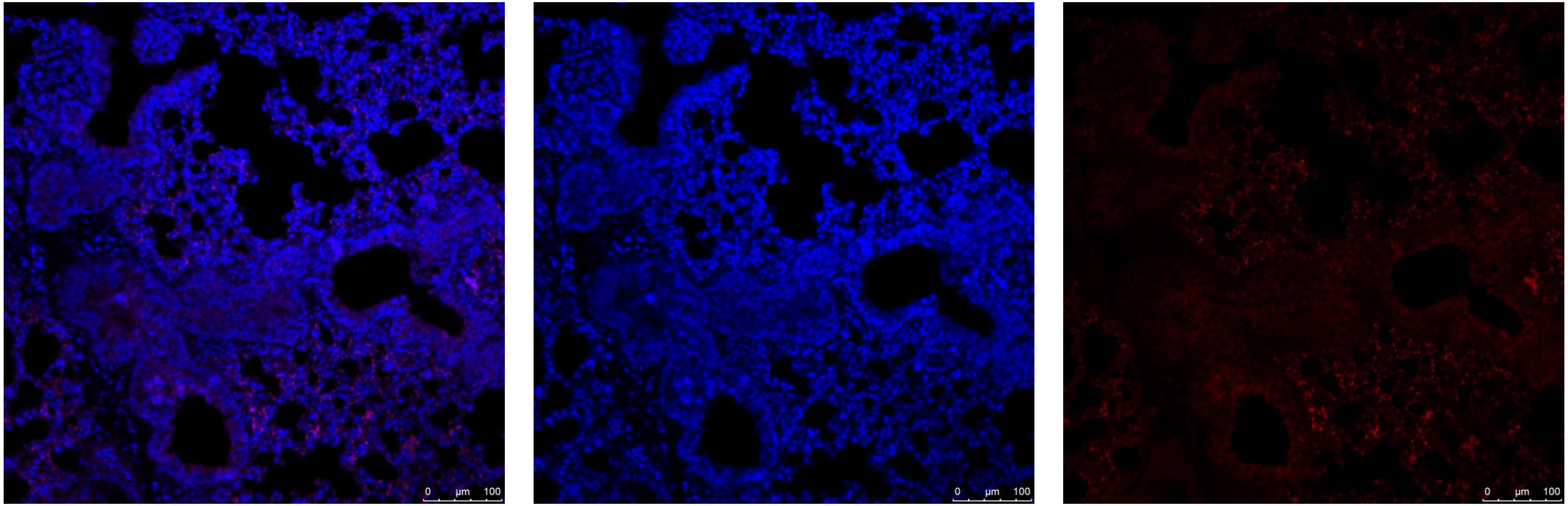

14day-BLM

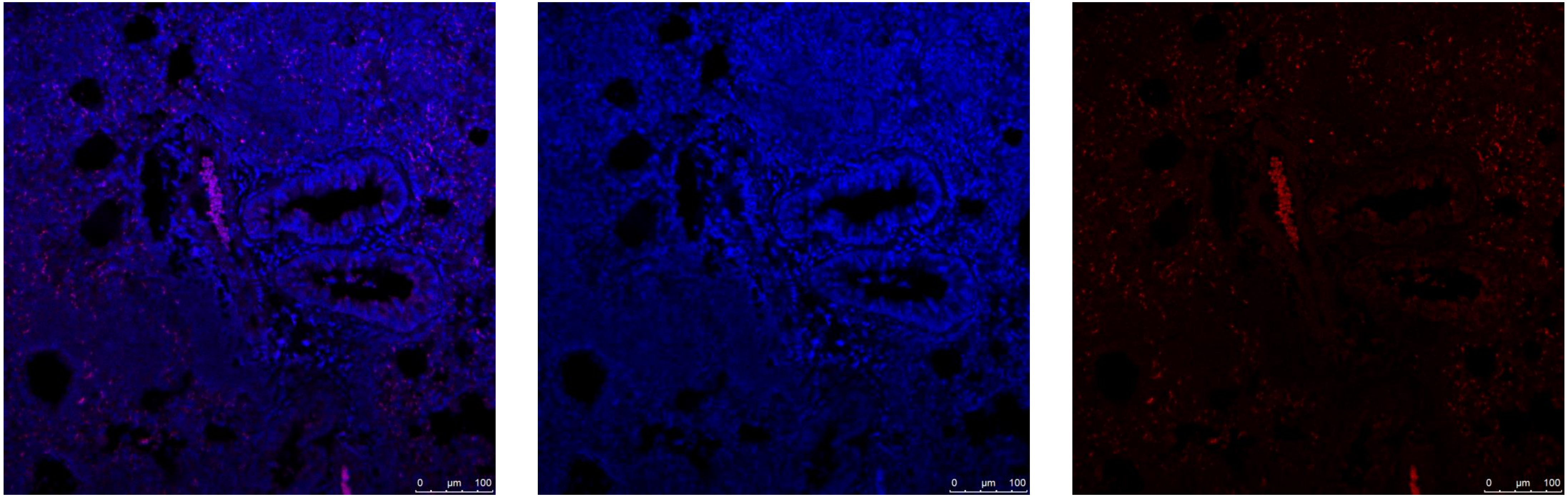

14day-BLM+PFD

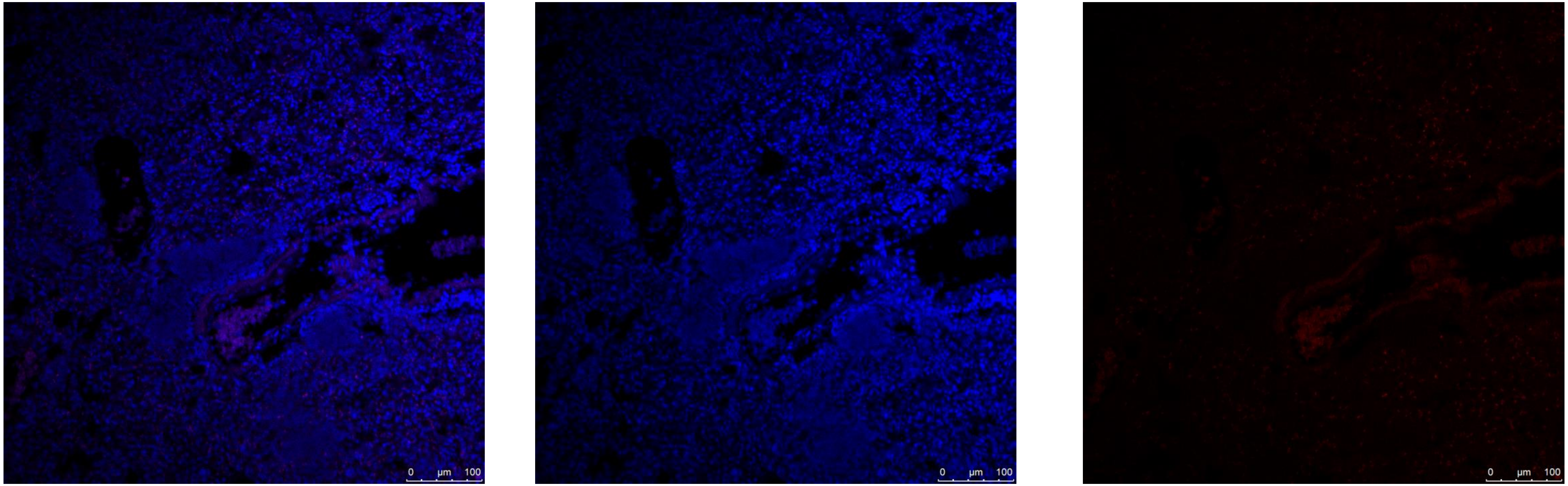

14day-BLM+TEL

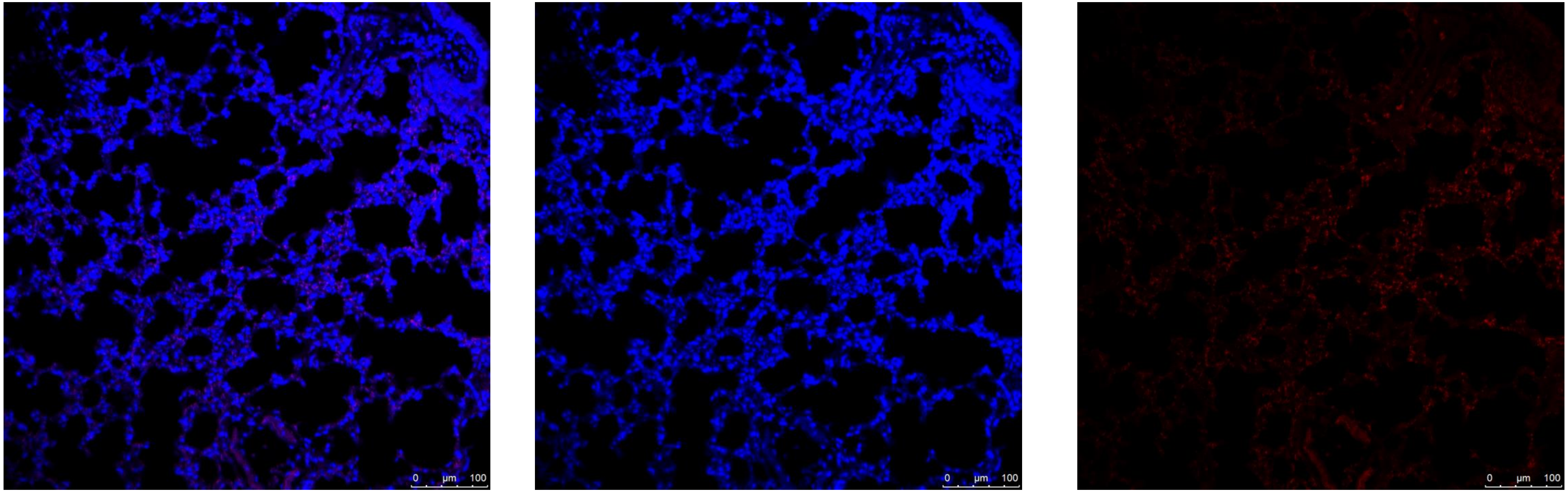

28day-Control

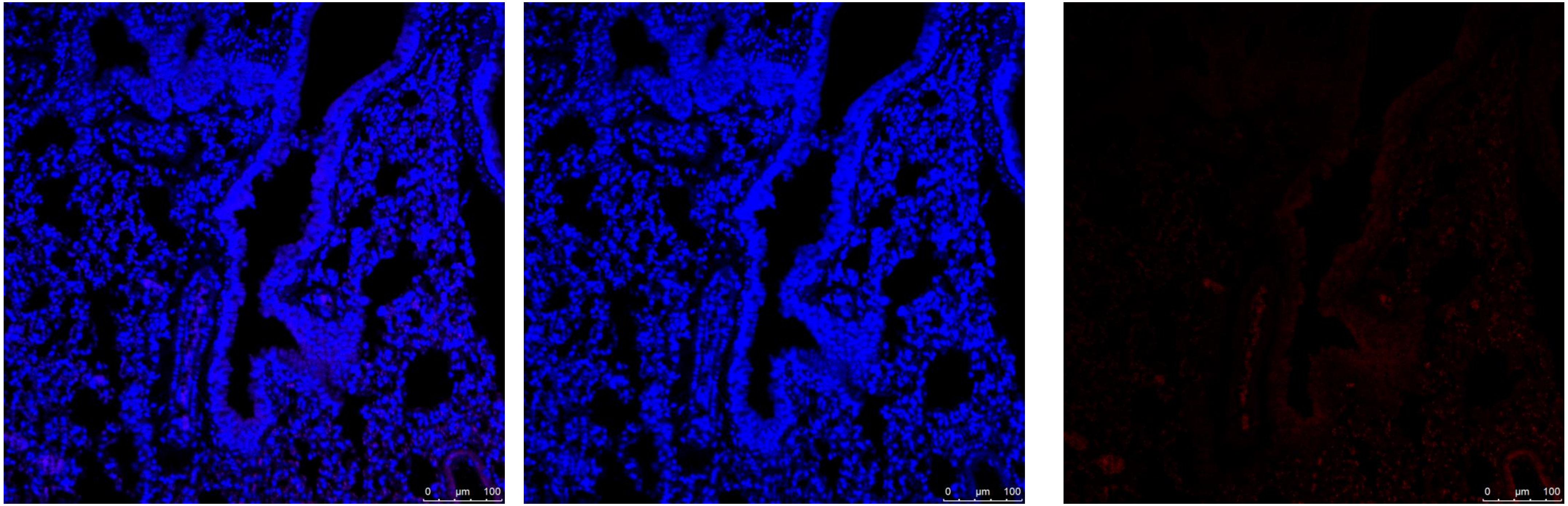

28day-BLM+H CAT

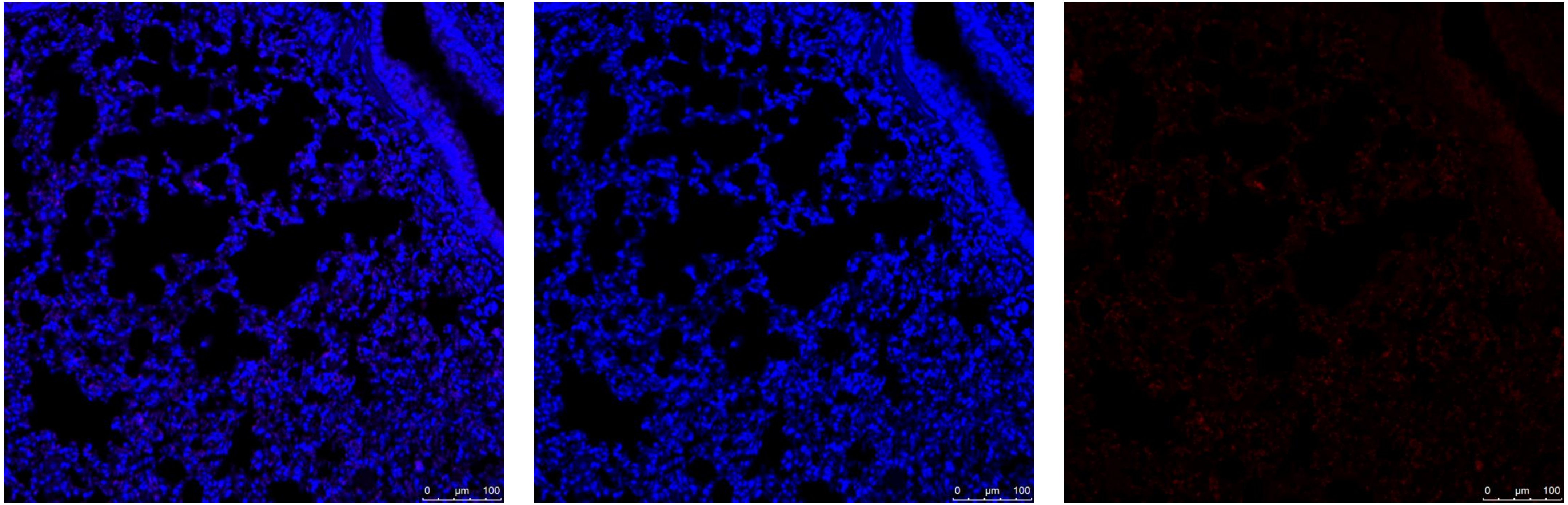

28day-BLM+L CAT

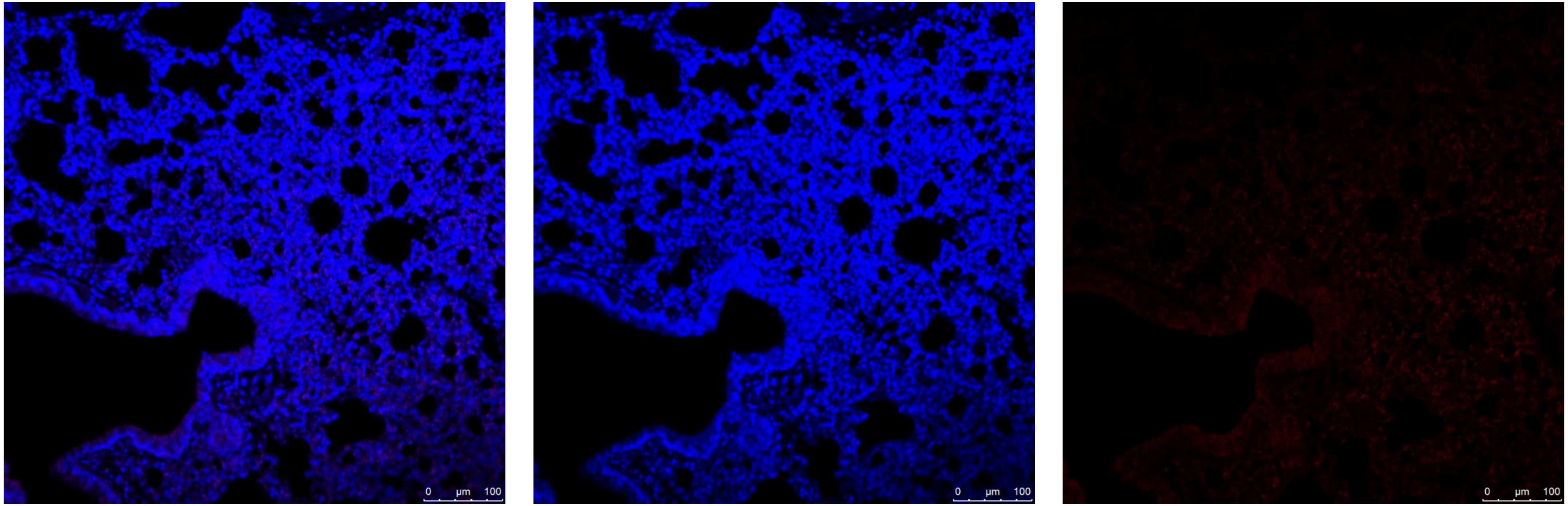

28day-BLM

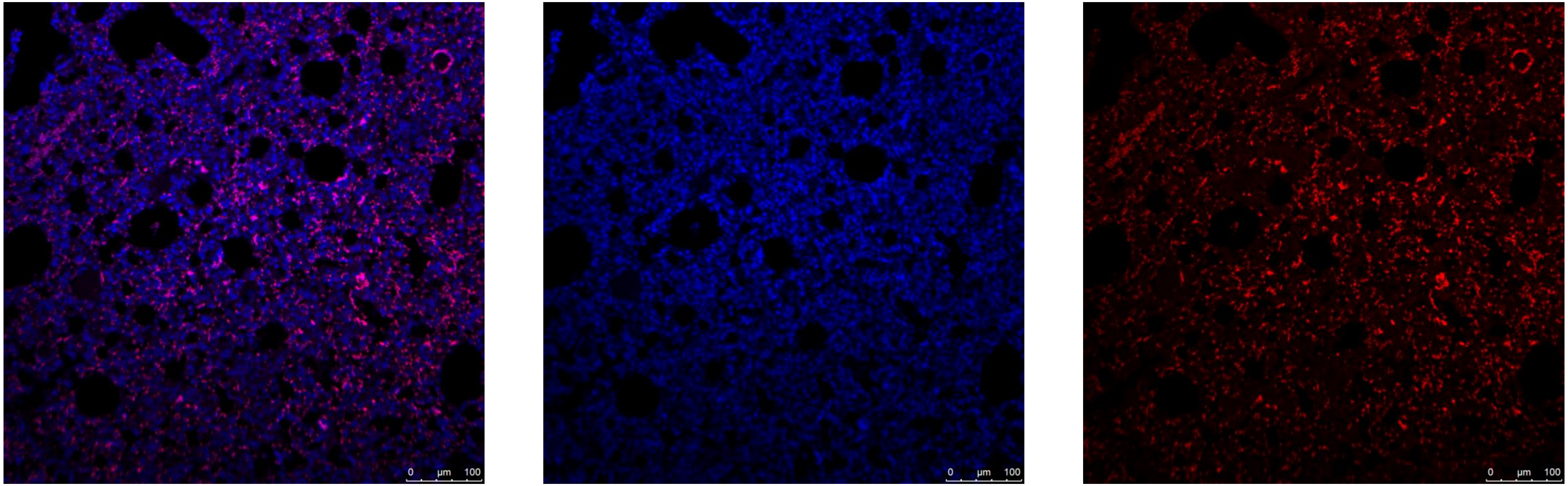

28day-BLM+PFD

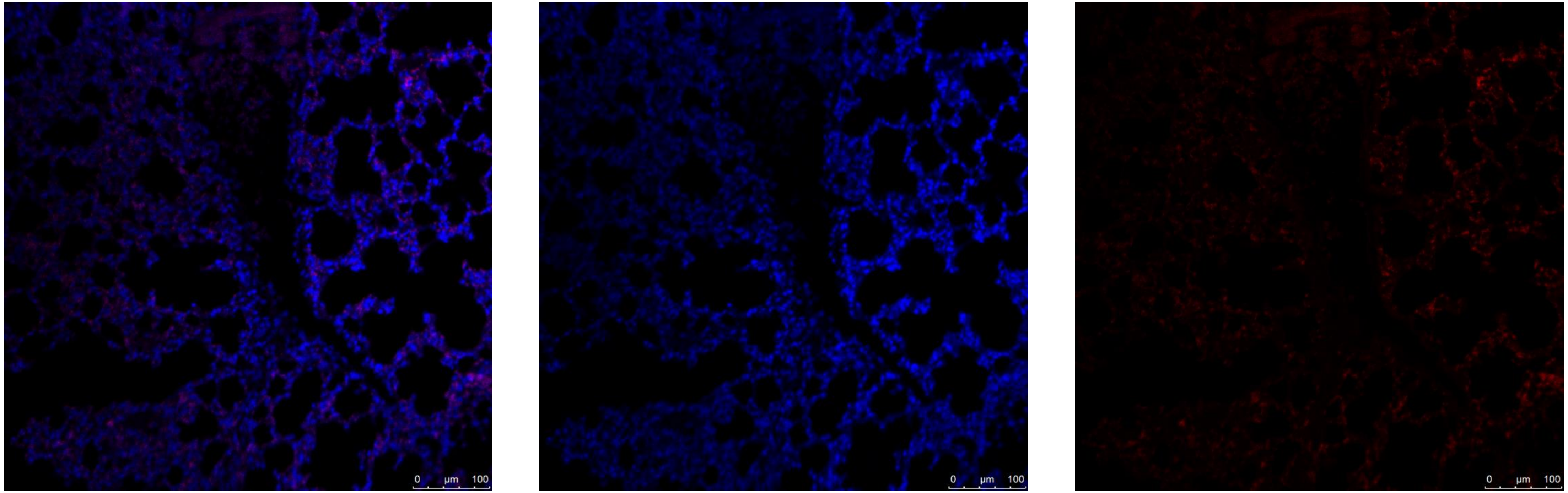

28day-BLM+TEL

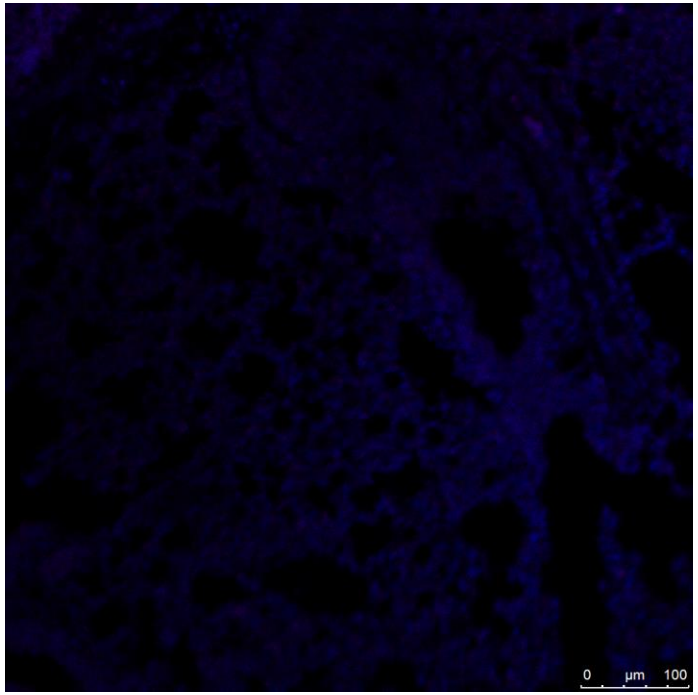

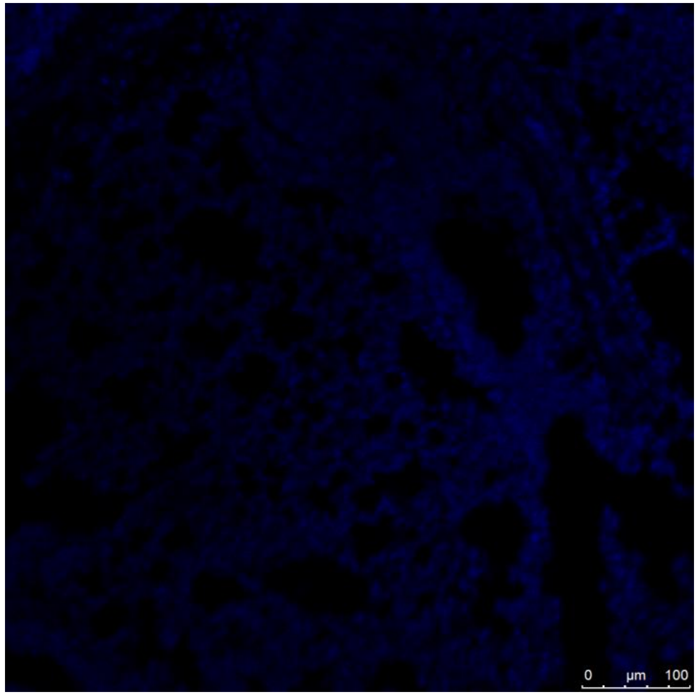

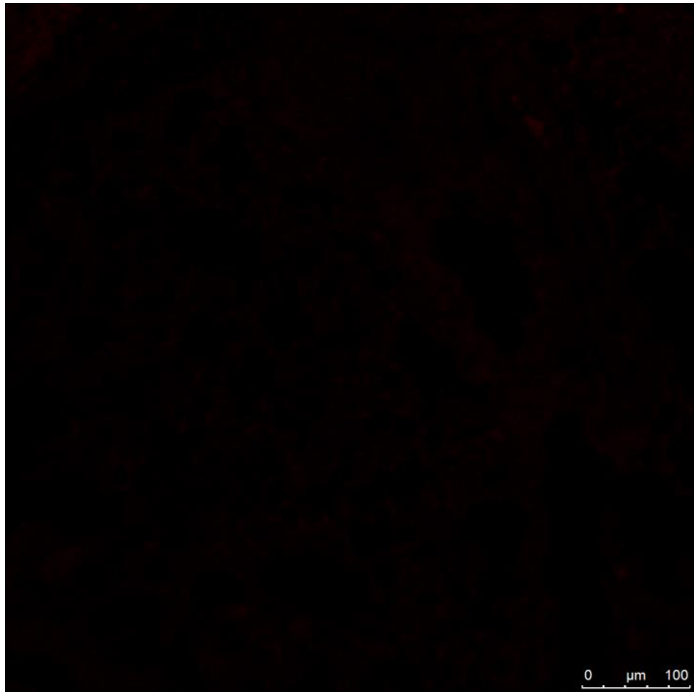

E-cadherin

7day-Control

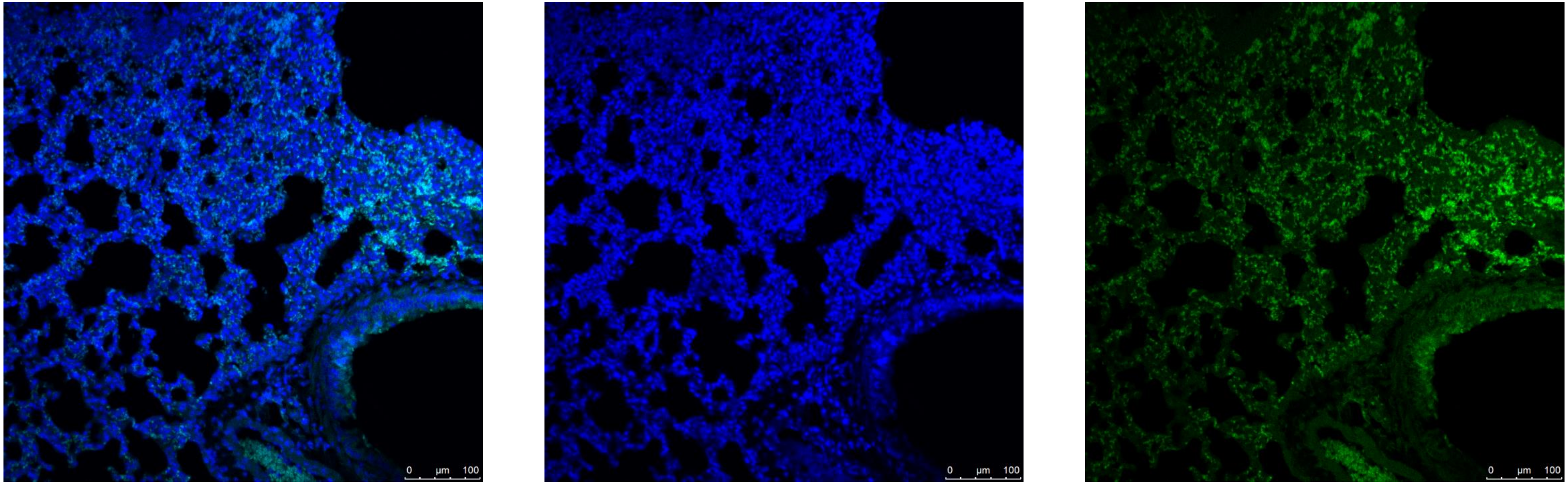

7day-BLM+H CAT

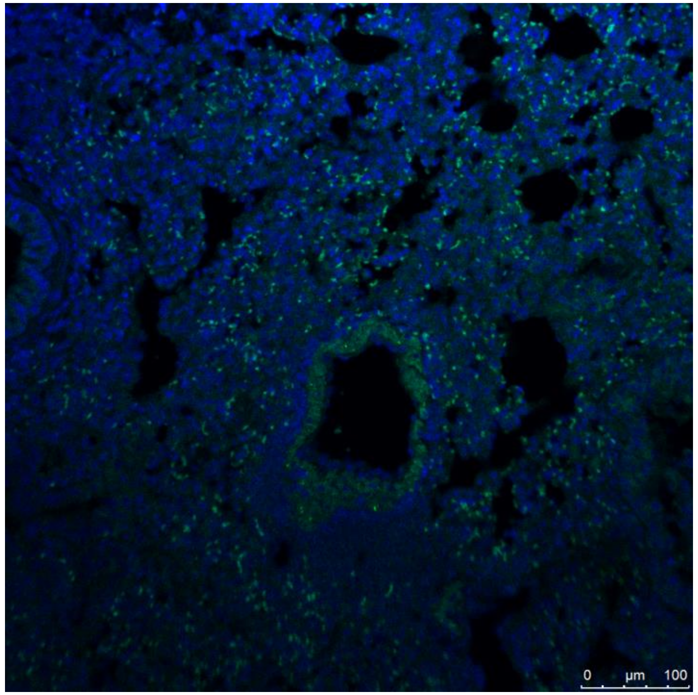

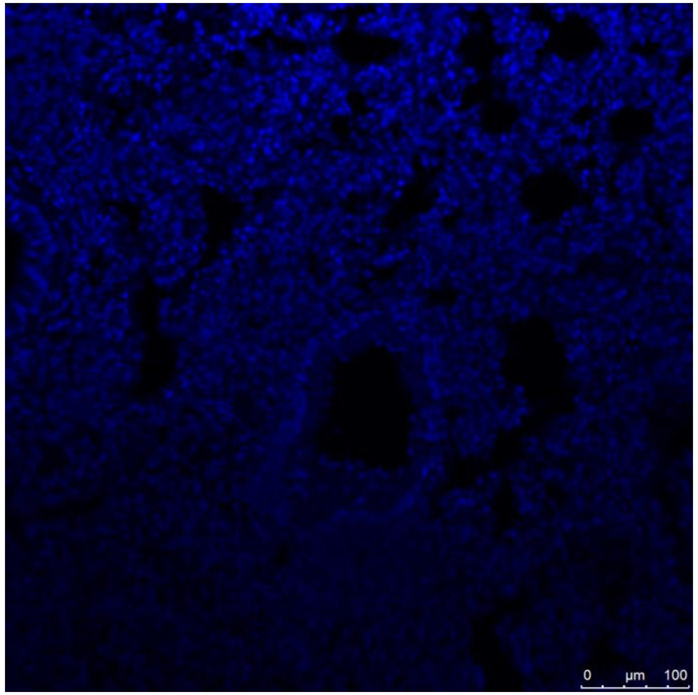

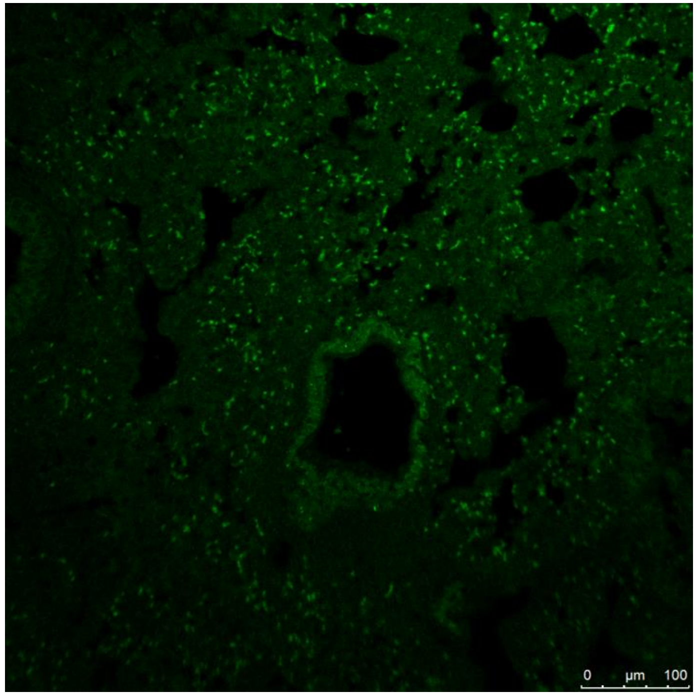

7day-BLM+L CAT

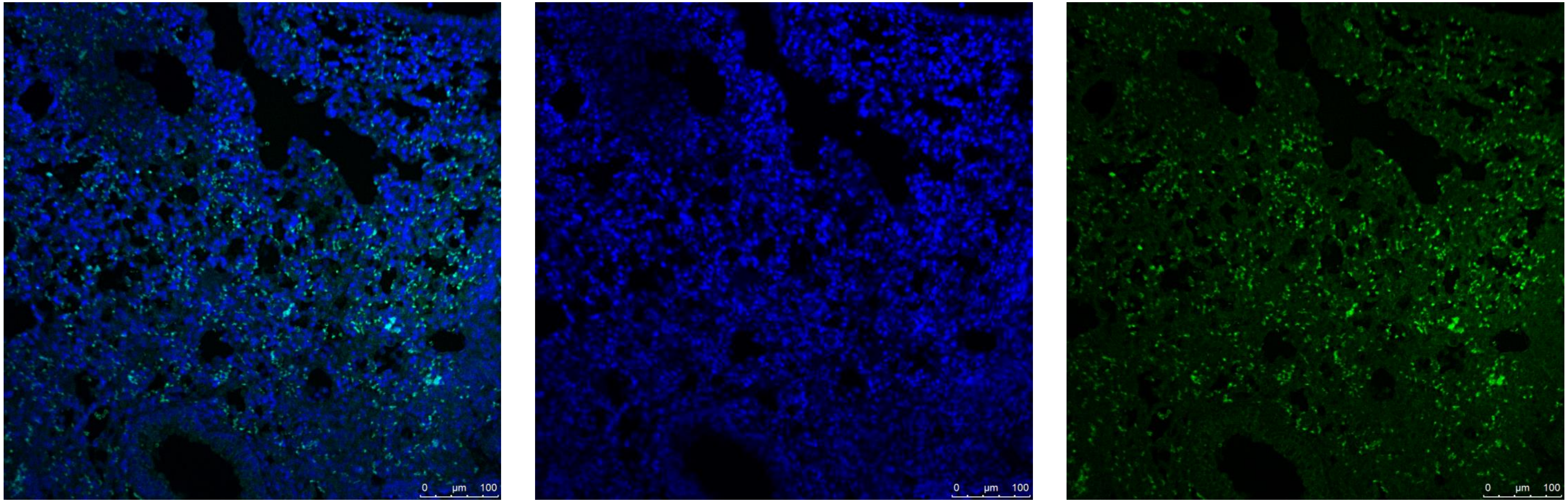

7day-BLM

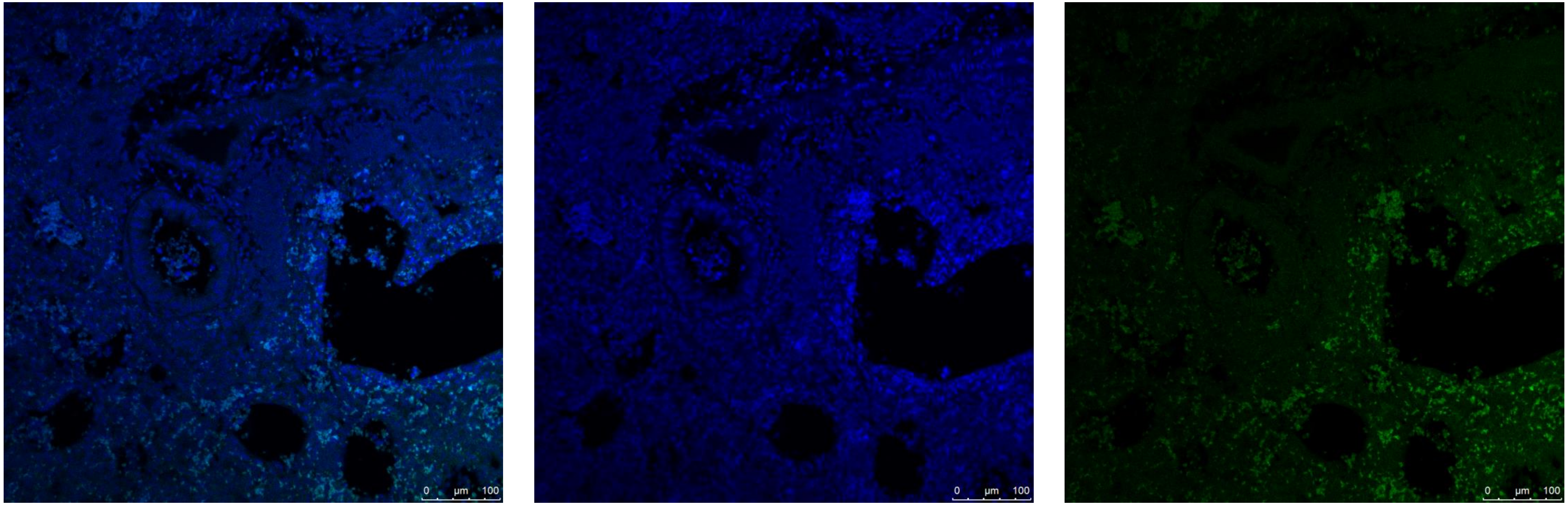

7day-BLM+PFD

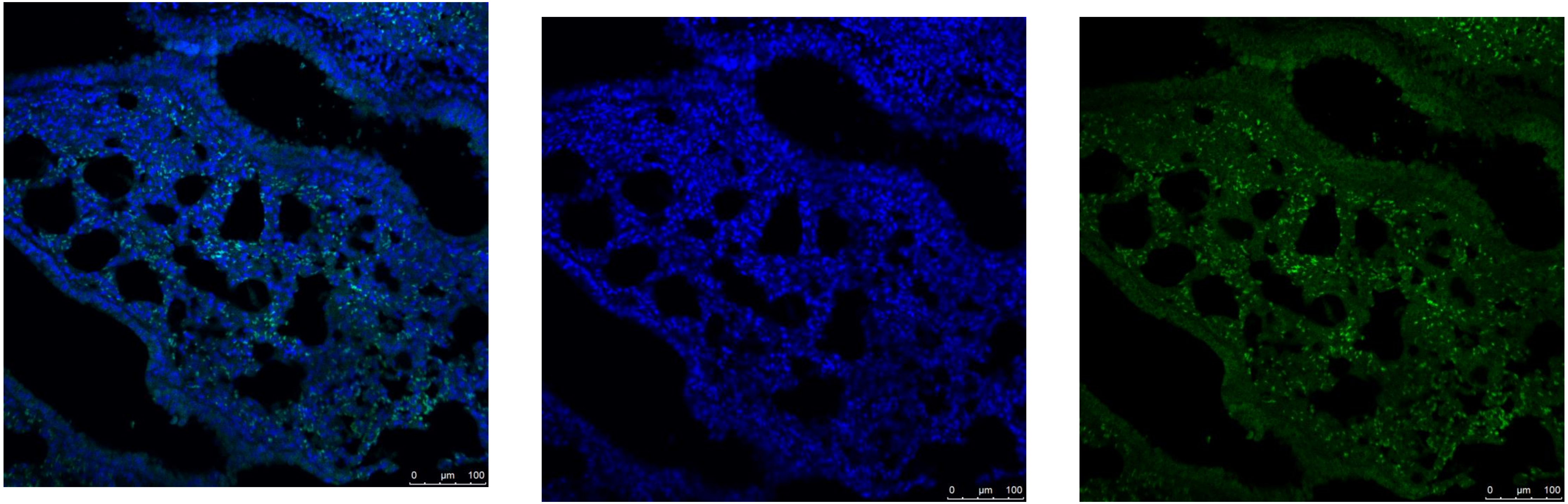

7day-BLM+TEL

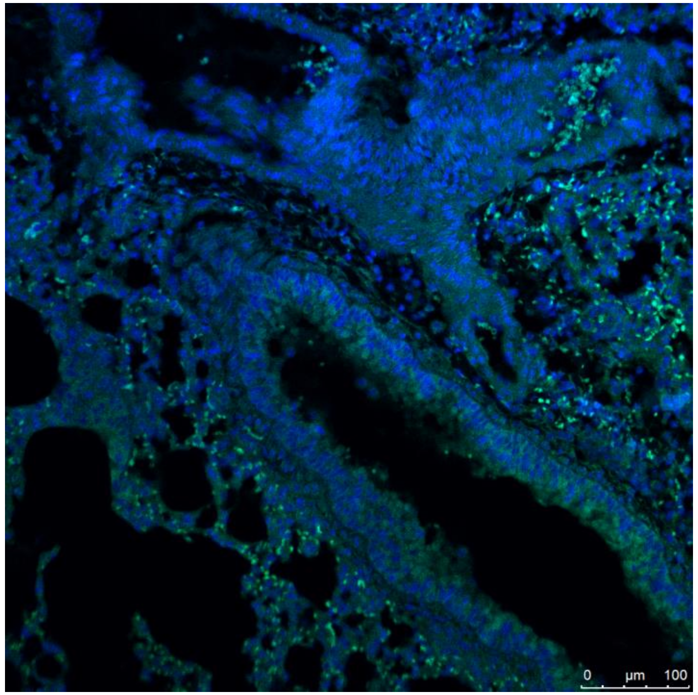

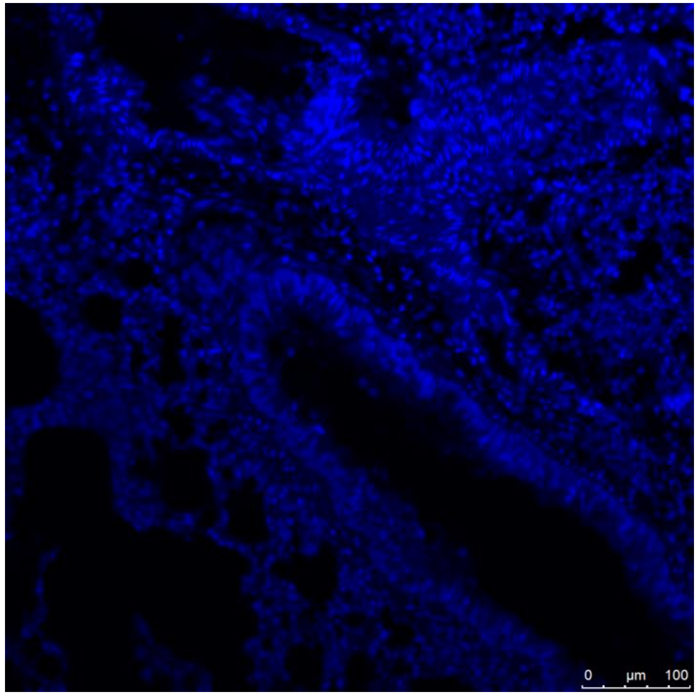

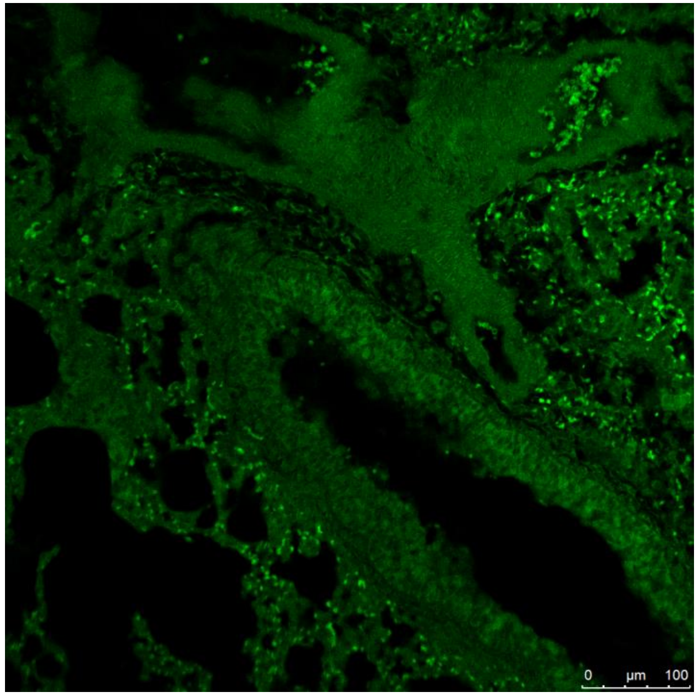

14day-Control

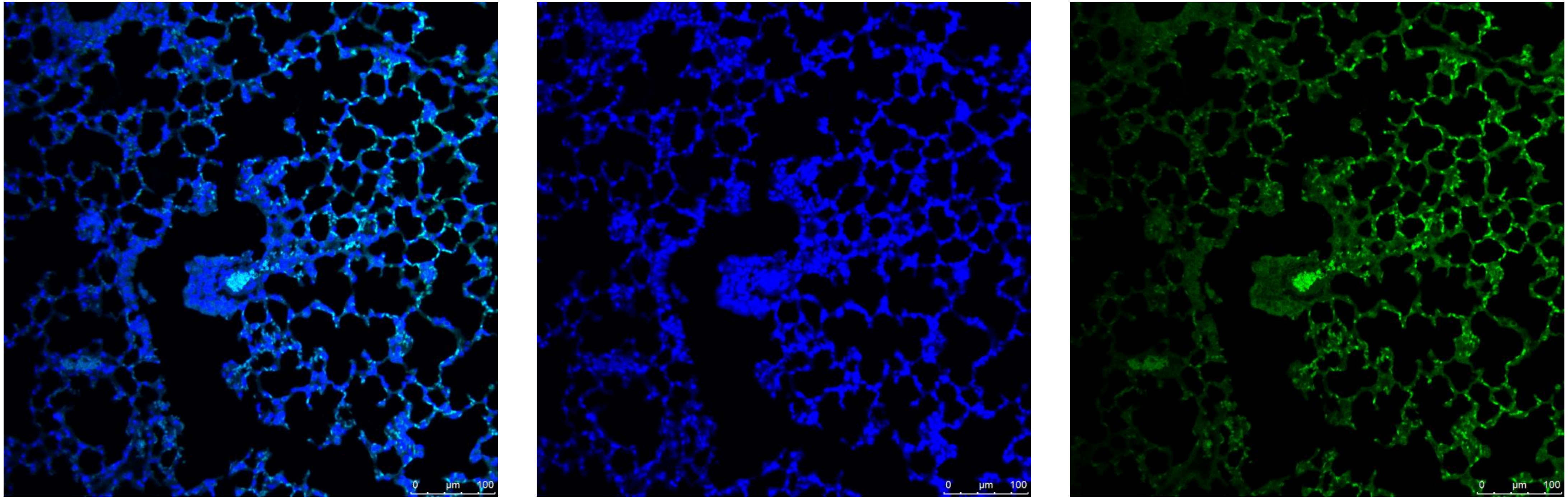

14day-BLM+H CAT

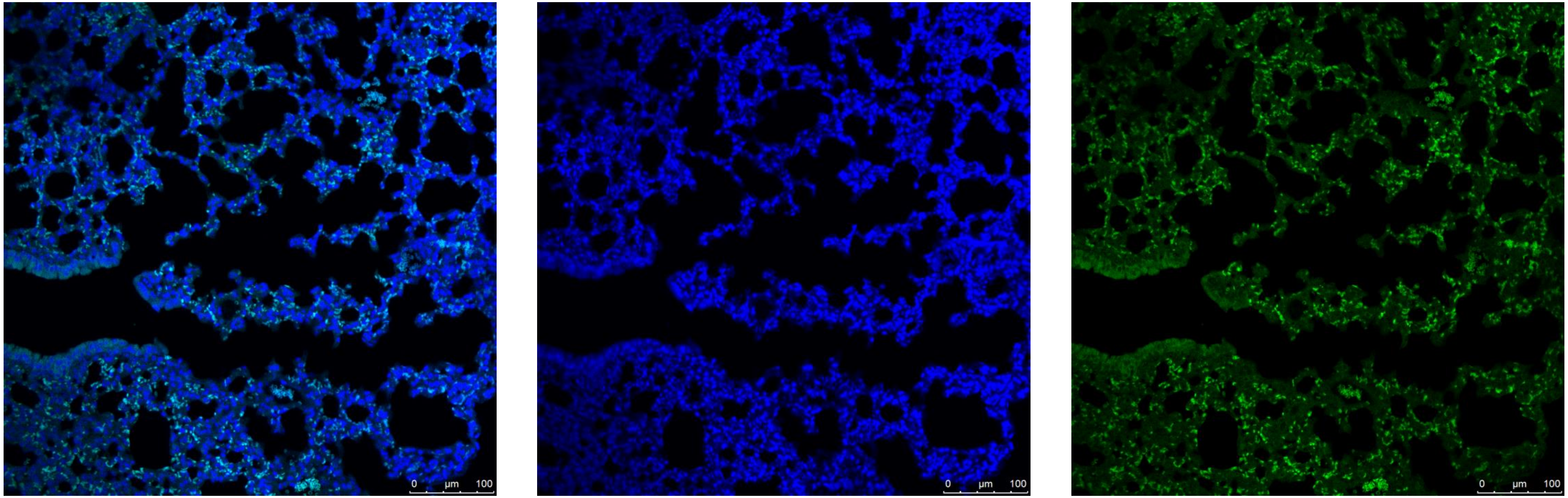

14day-BLM+L CAT

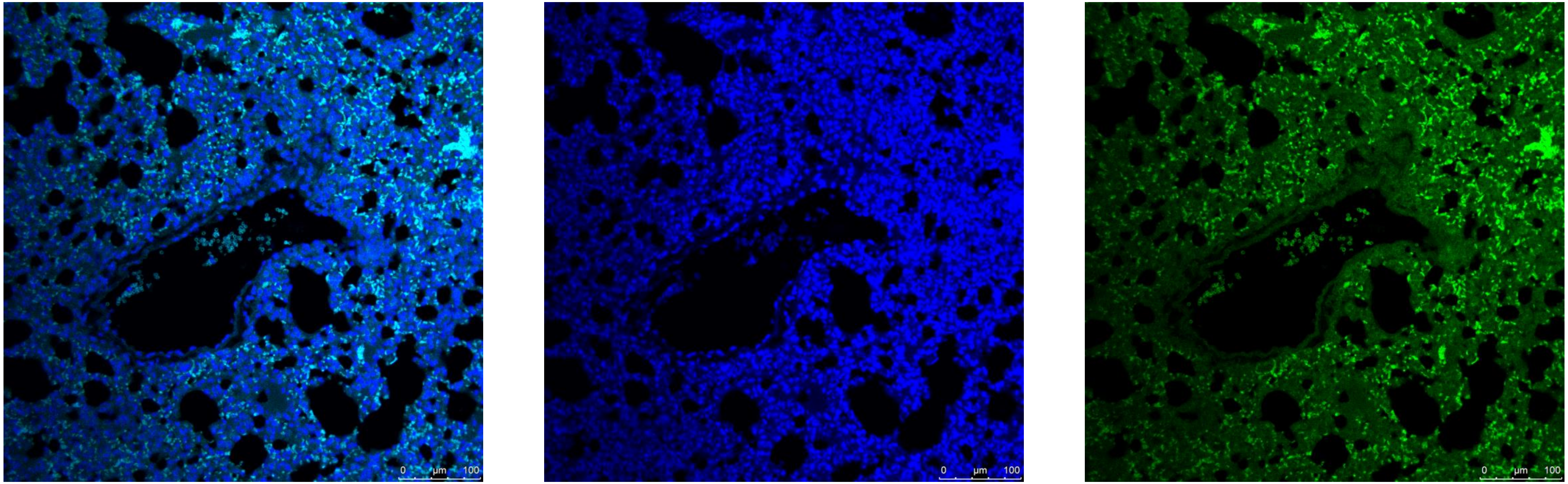

14day-BLM

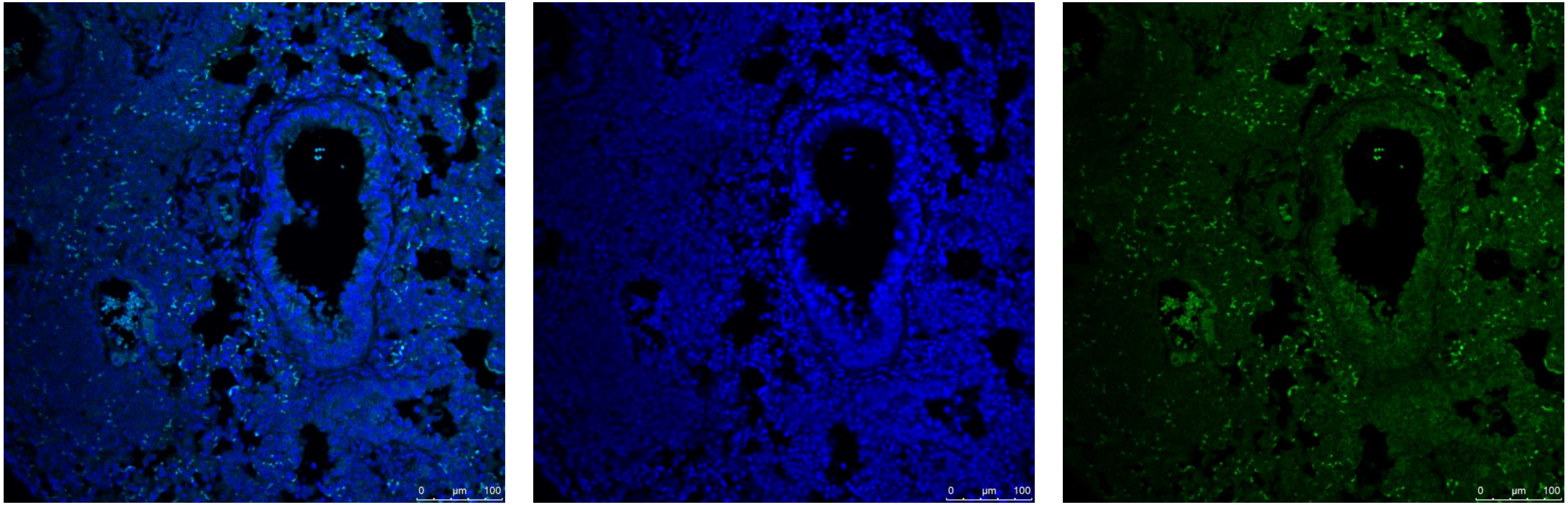

14day-BLM+PFD

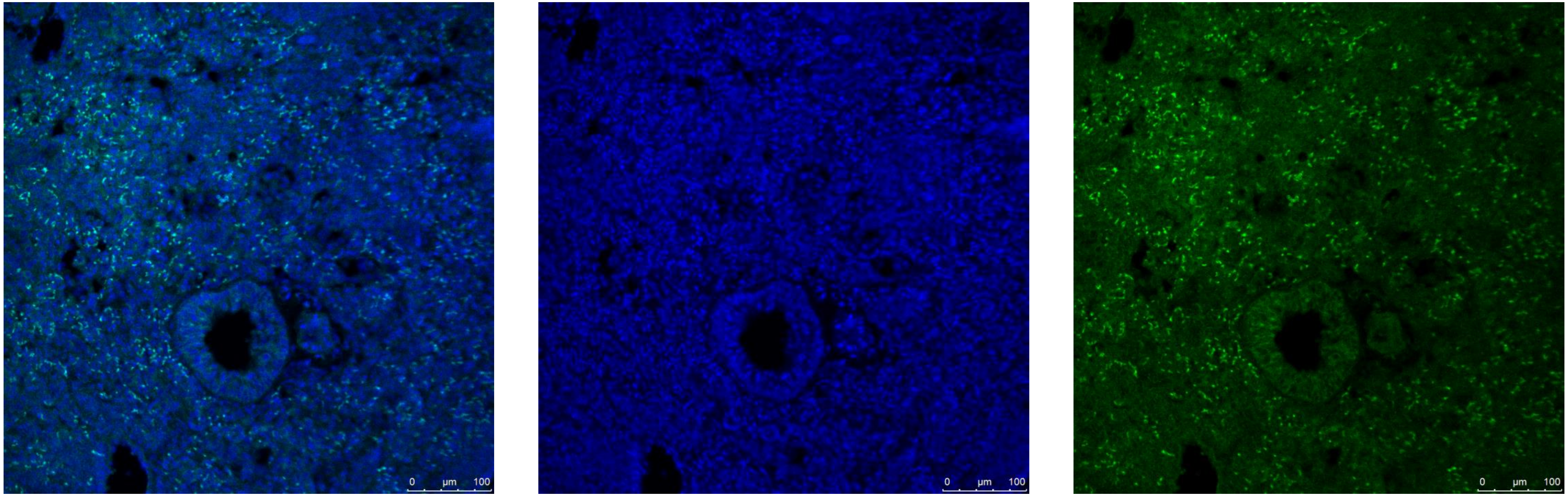

14day-BLM+TEL

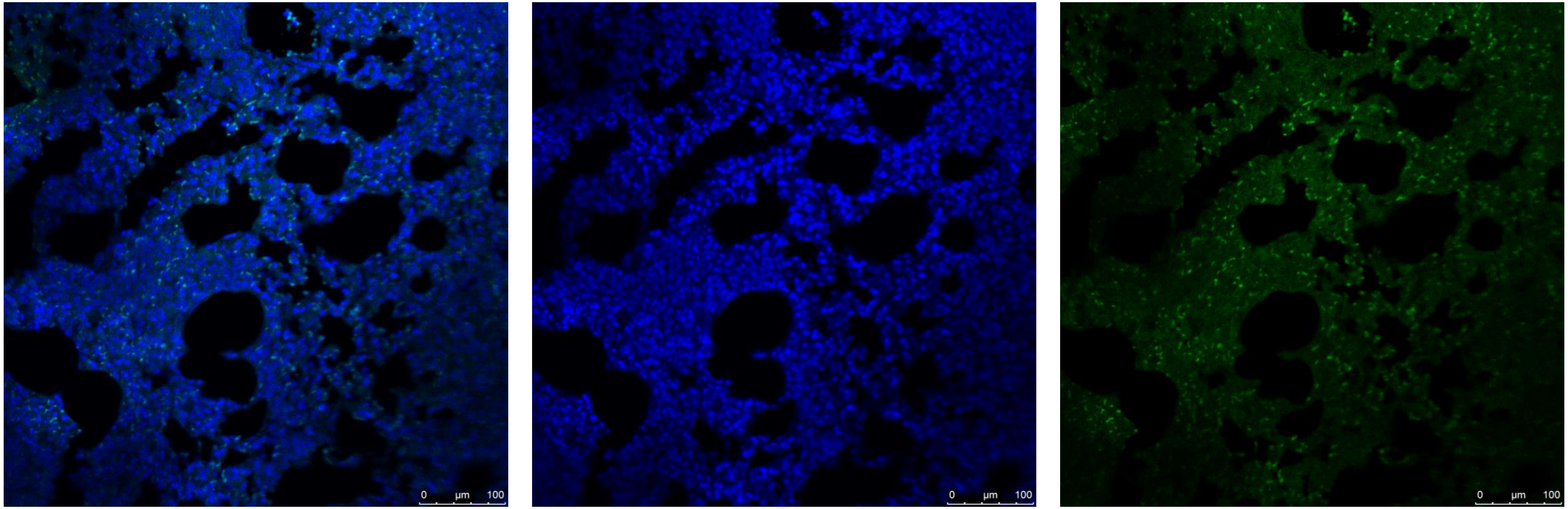

28day-Control

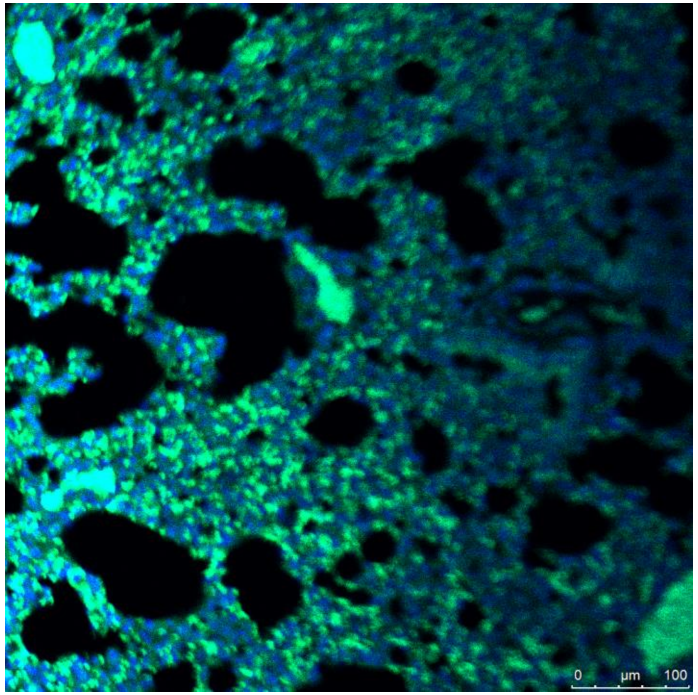

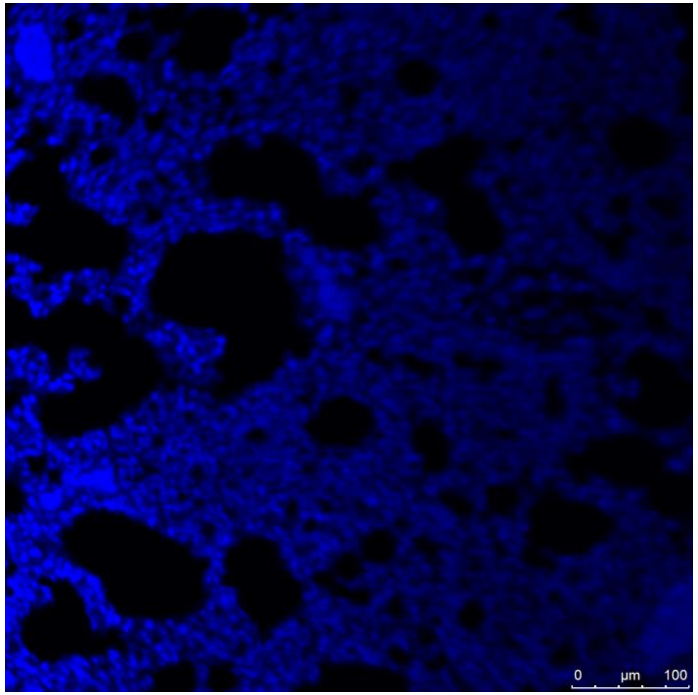

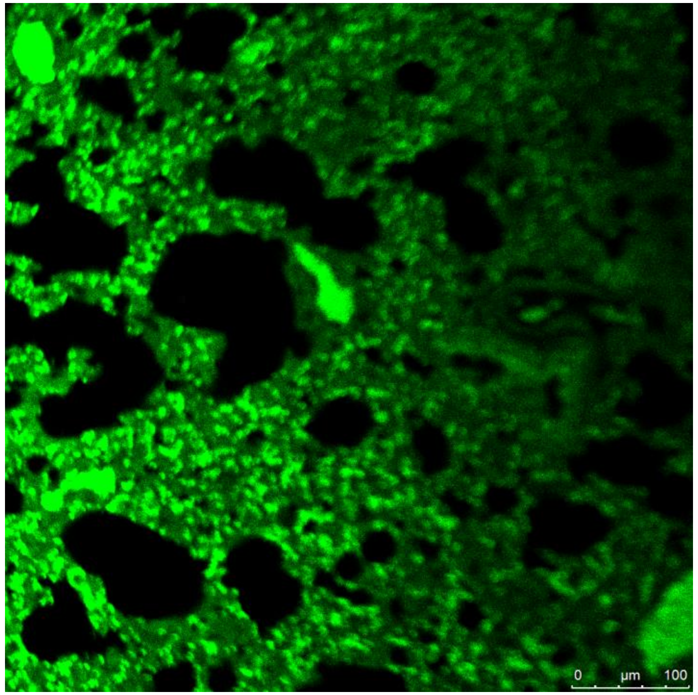

28day-BLM+H CAT

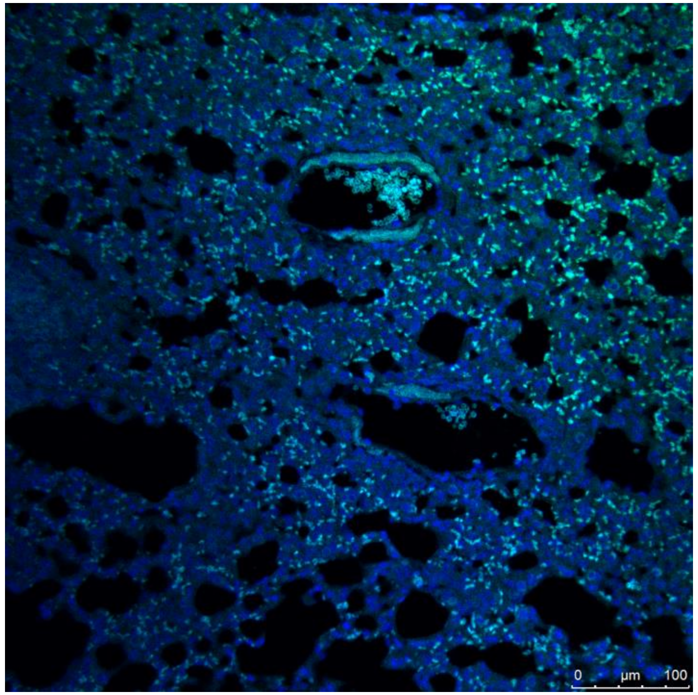

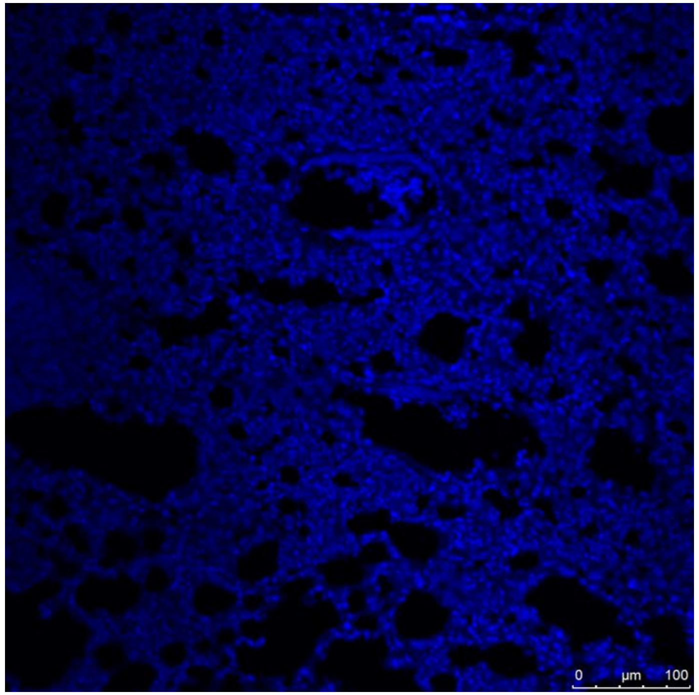

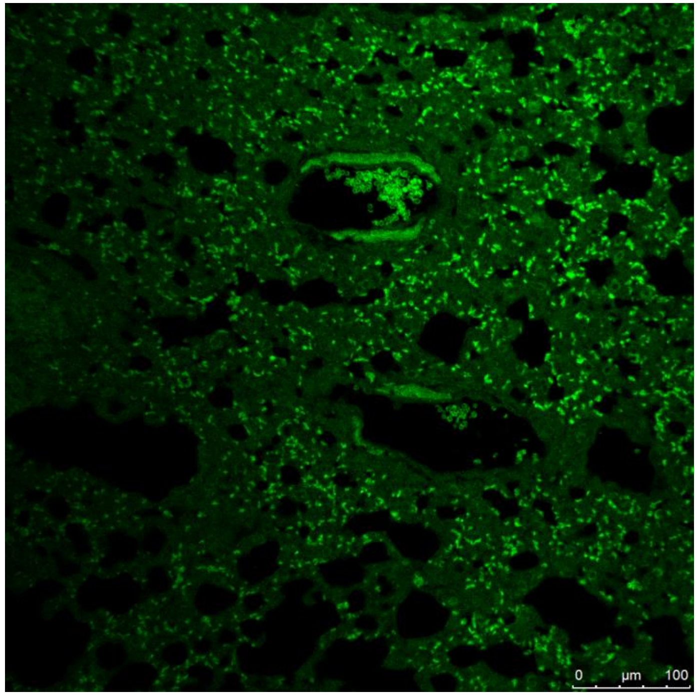

28day-BLM+L CAT

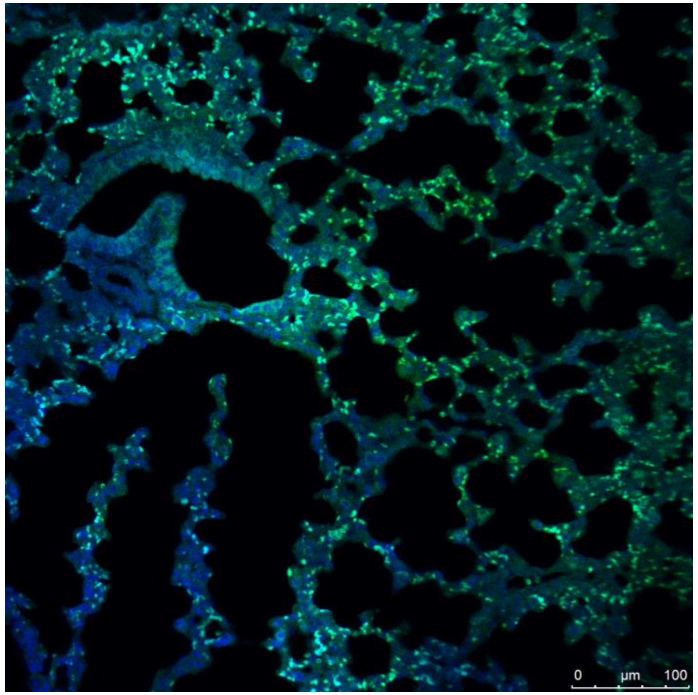

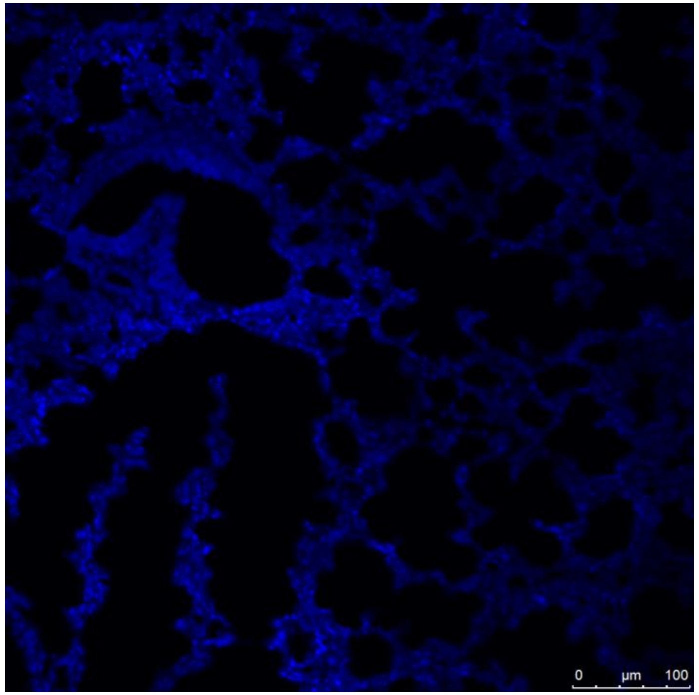

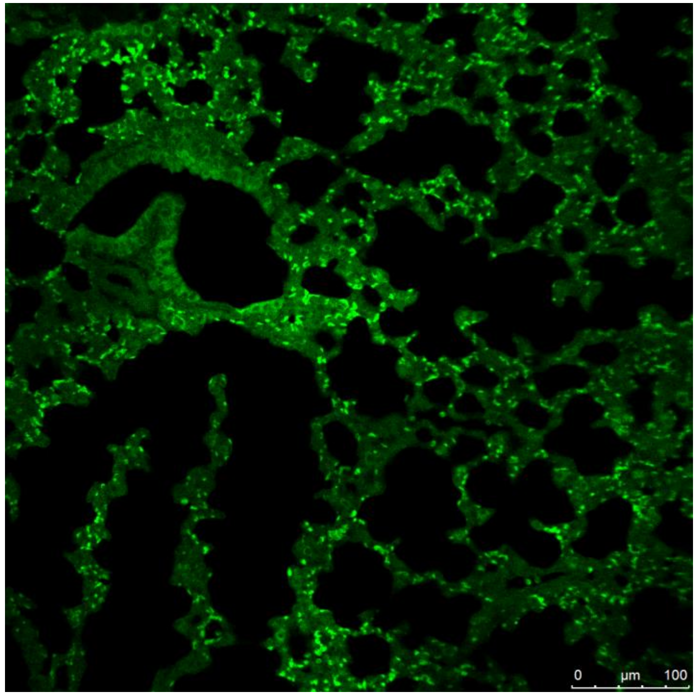

28day-BLM

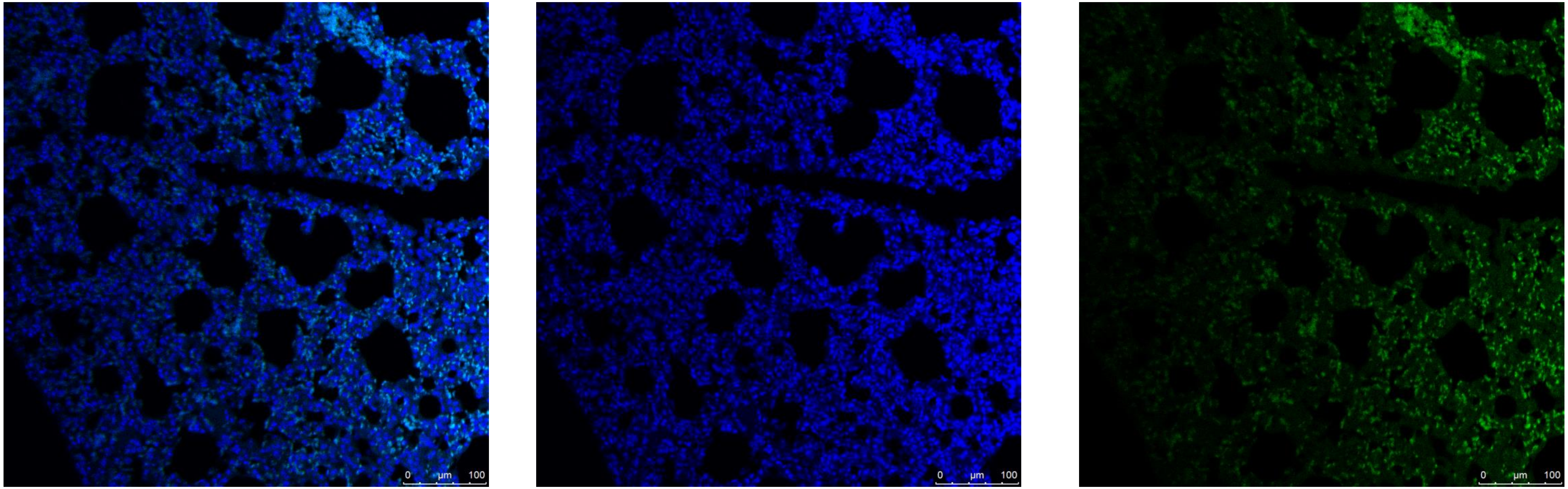

28day-BLM+PFD

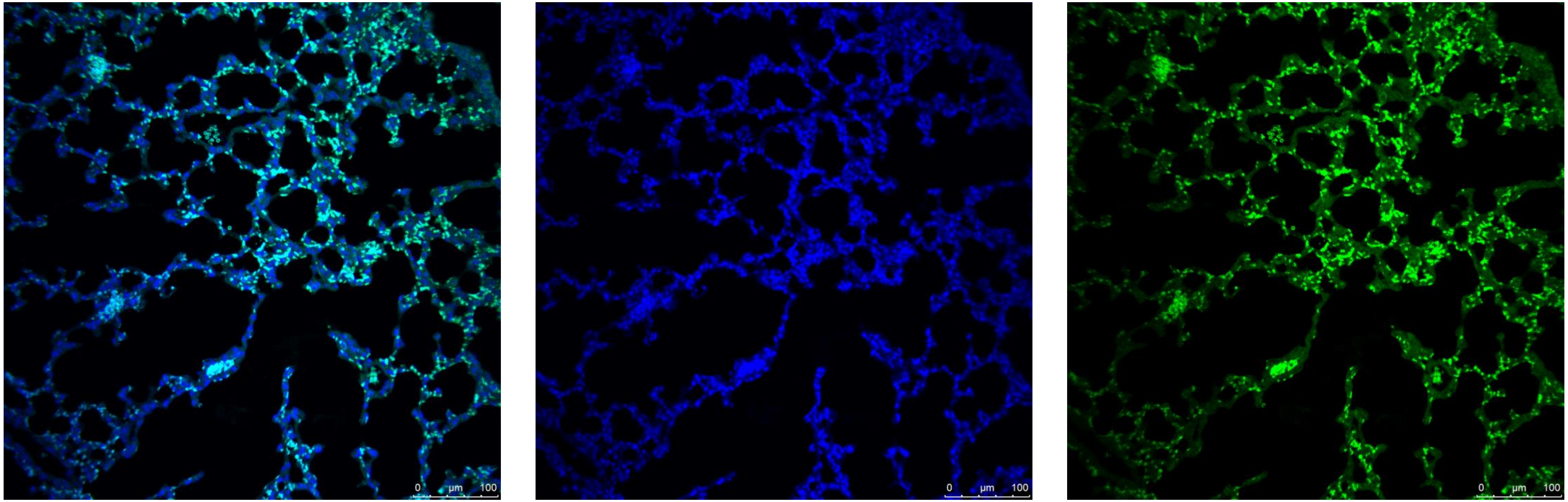

28day-BLM+TEL

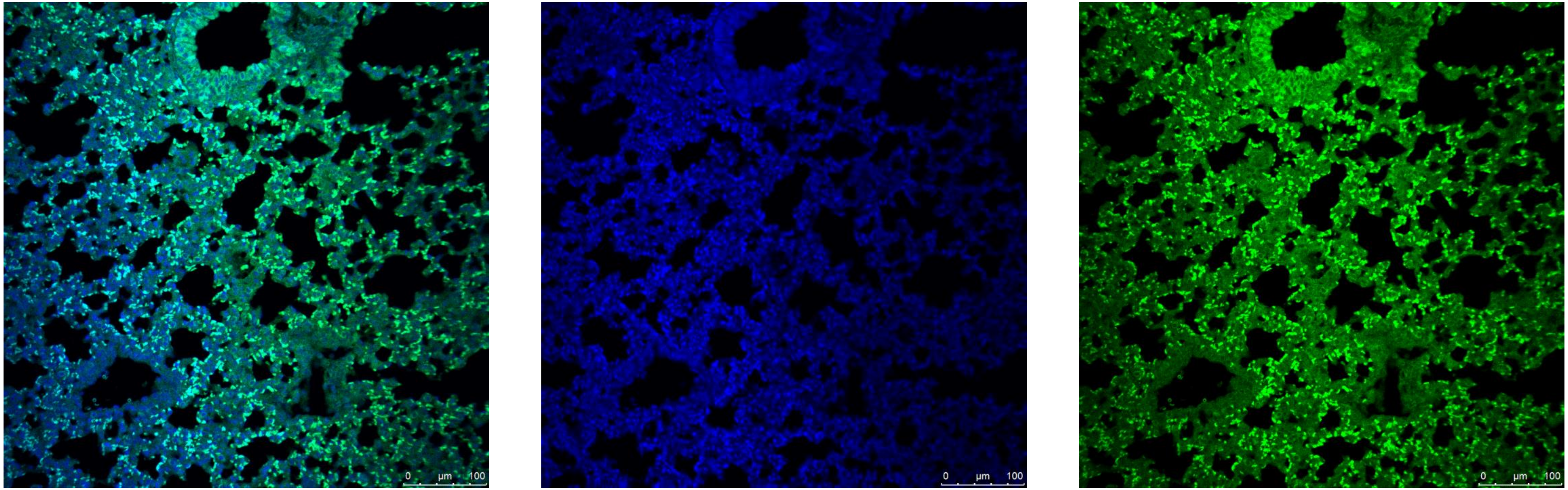

Supplement: Supplementary file 4 [file Data_Sheet_2.PDF]
