# Supplementary material for: Catalpol Attenuates Pulmonary Fibrosis by Inhibiting Ang II/AT1 and TGF-β/Smad-Mediated Epithelial Mesenchymal Transition
Source: Front Med (Lausanne). 2022 May 24;9:878601. doi: 10.3389/fmed.2022.878601 (PMC9171363; doi:10.3389/fmed.2022.878601)

7day act

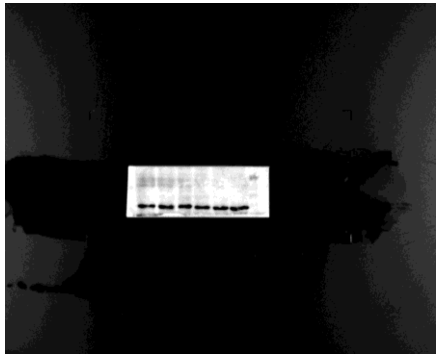

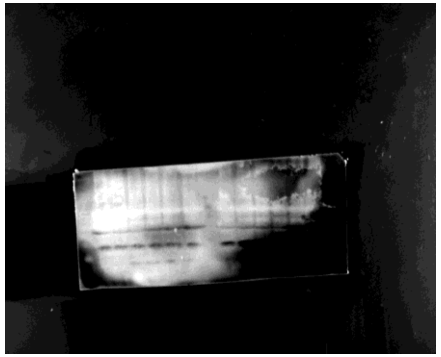

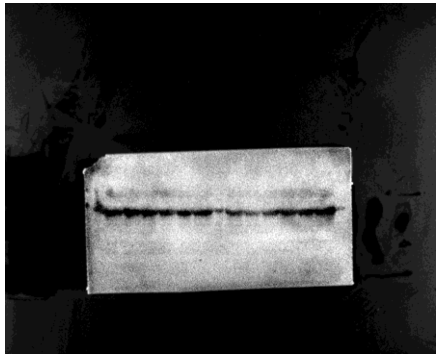

7day AT1

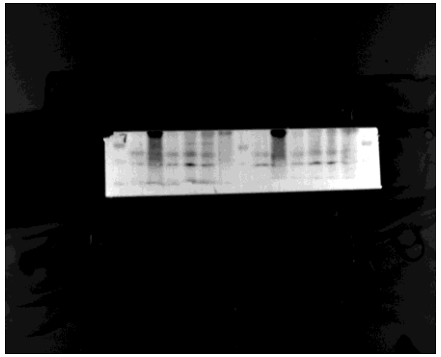

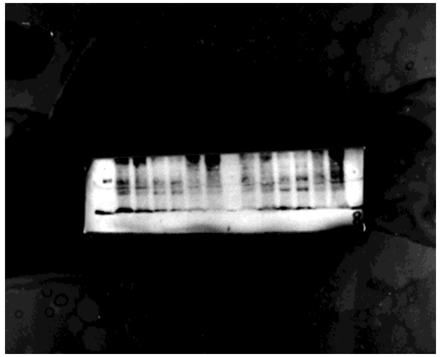

7day MMP2

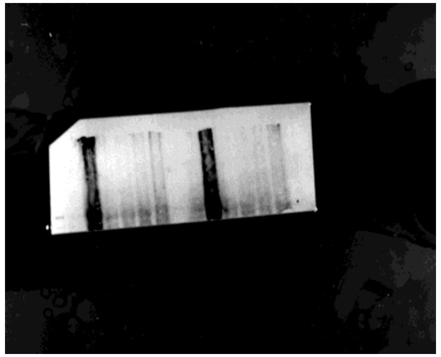

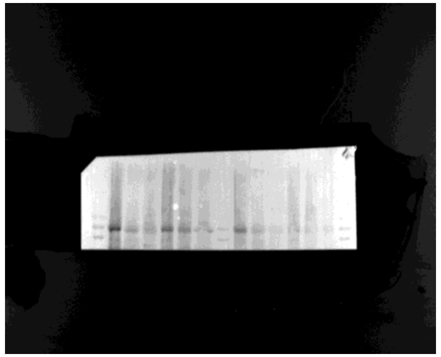

7day MMP9

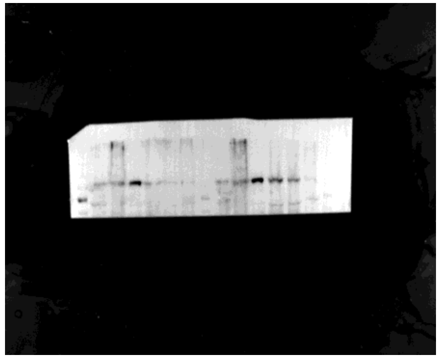

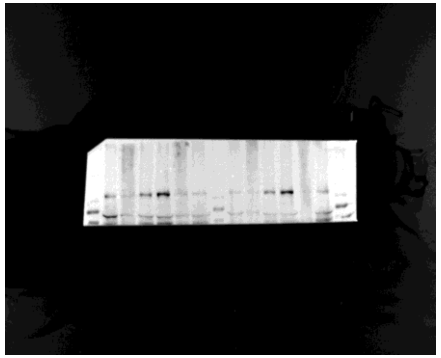

7day P-Smad2

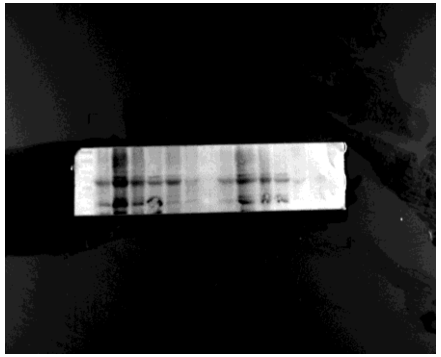

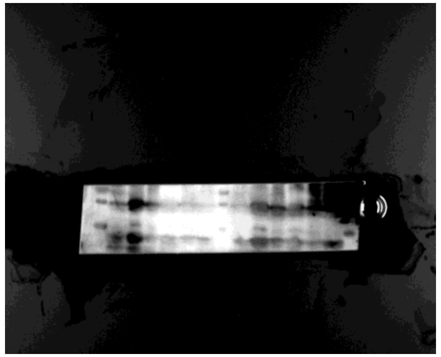

7day P-Smad3

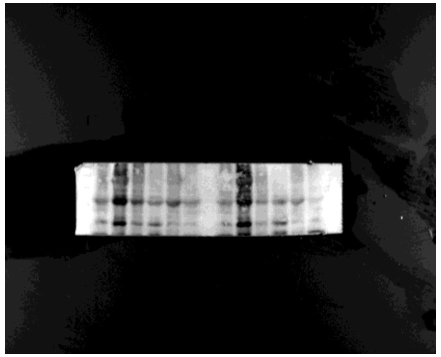

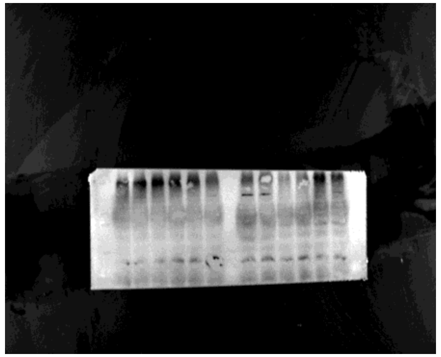

7day Smad2,3

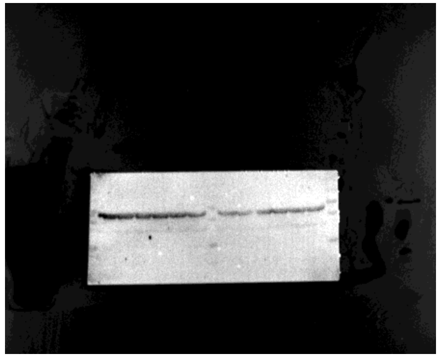

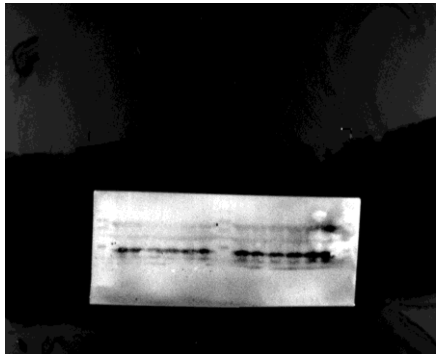

7day Snail

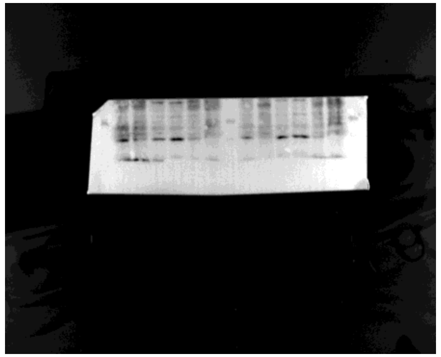

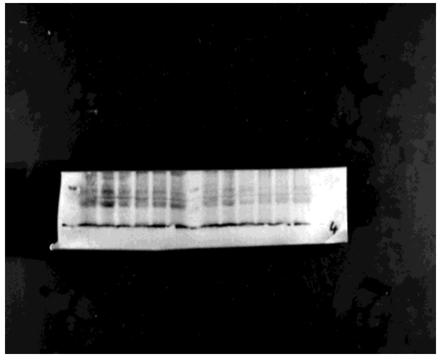

7day TGF-β1

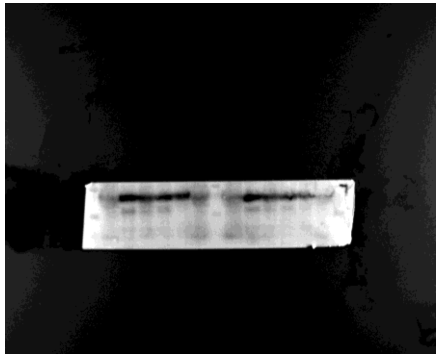

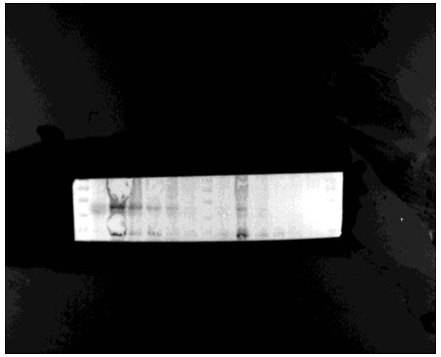

14day act

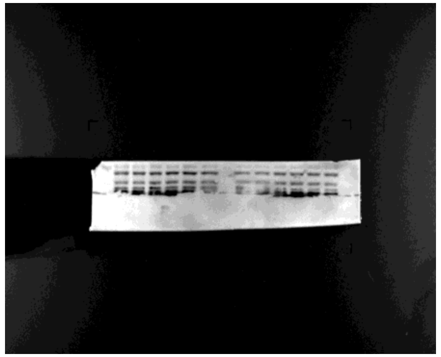

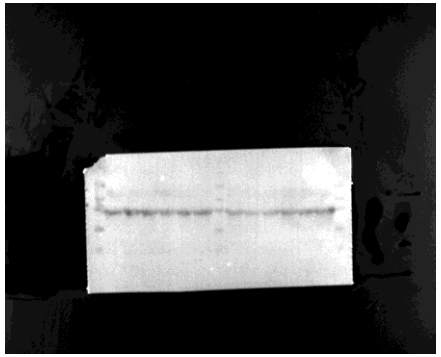

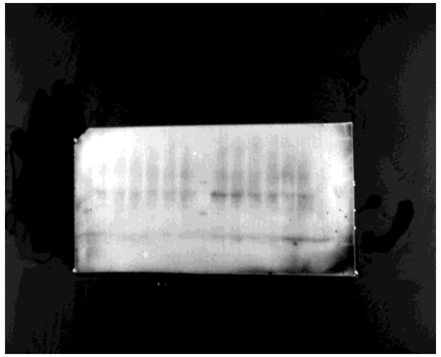

14day AT1

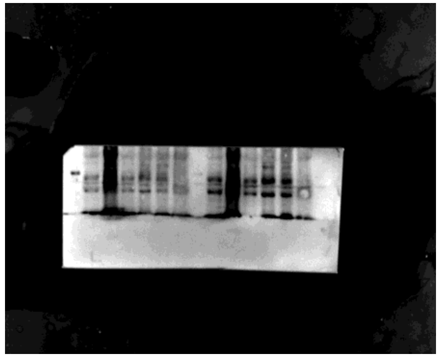

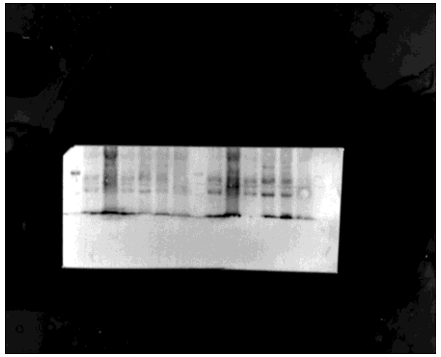

14day MMP2

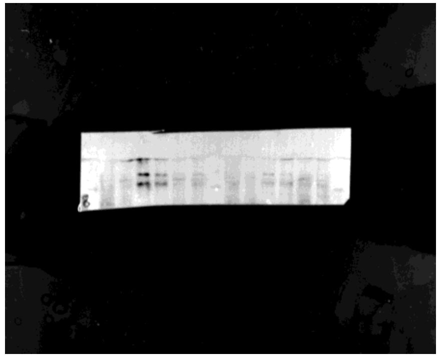

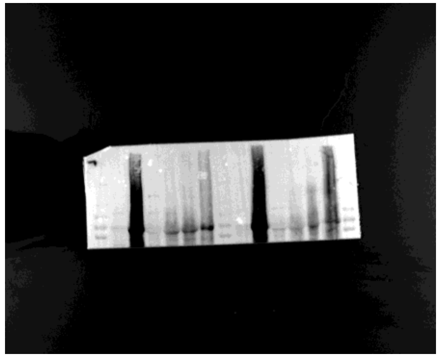

14day MMP9

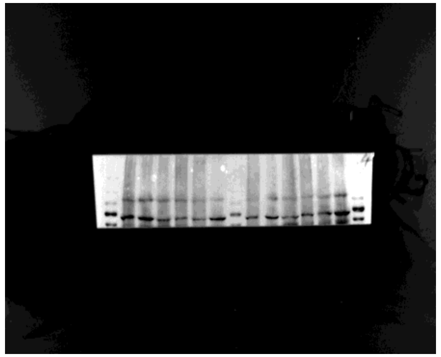

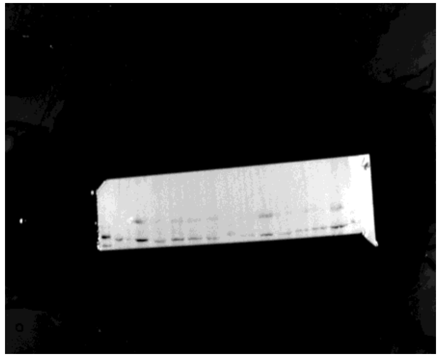

14day P-Smad2

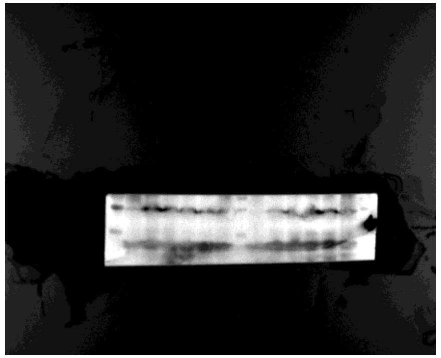

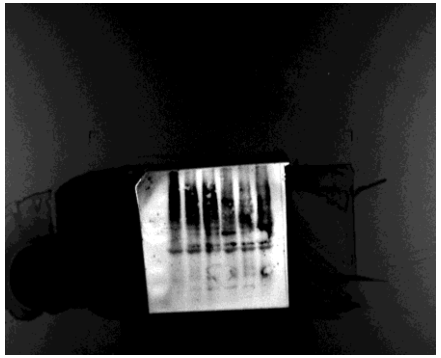

14day P-Smad3

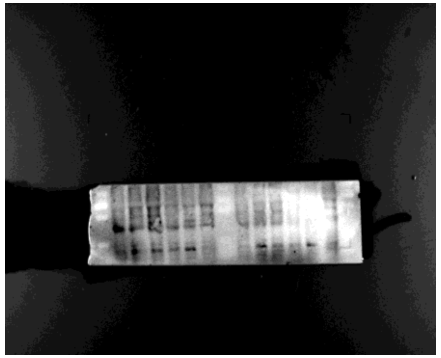

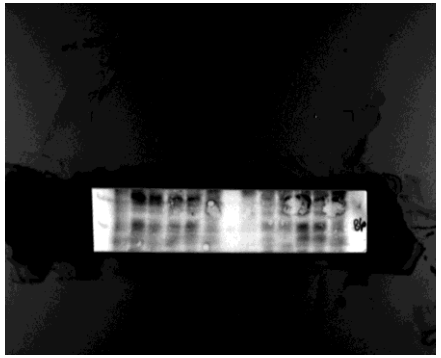

14day Smad2,3

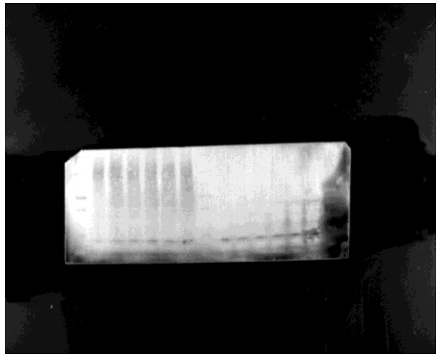

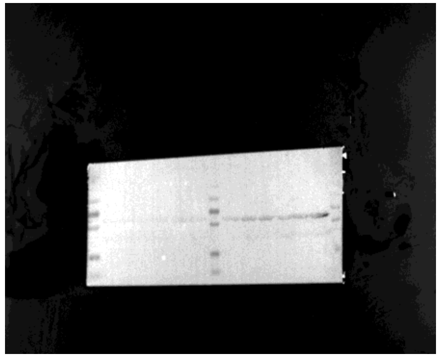

14day Snail

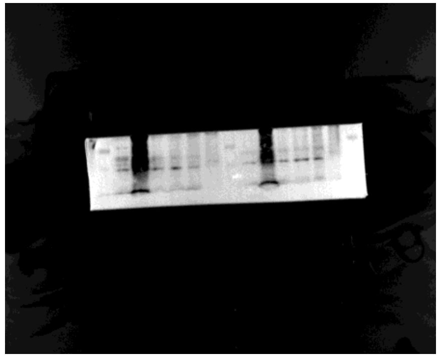

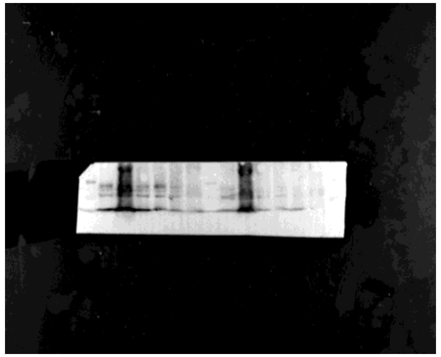

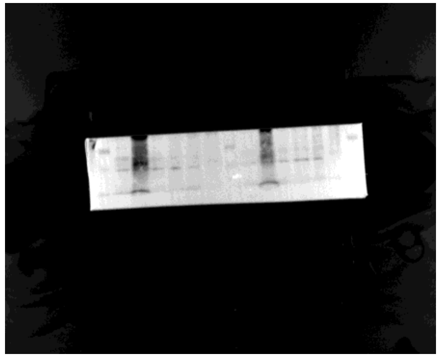

14day TGF-β1

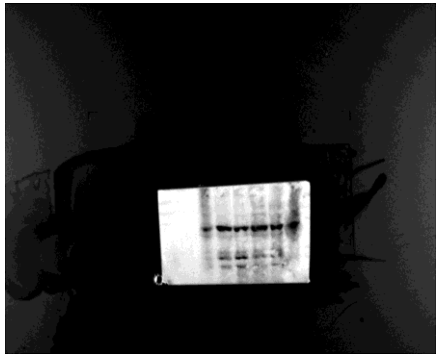

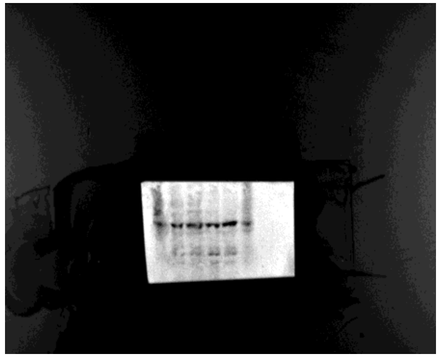

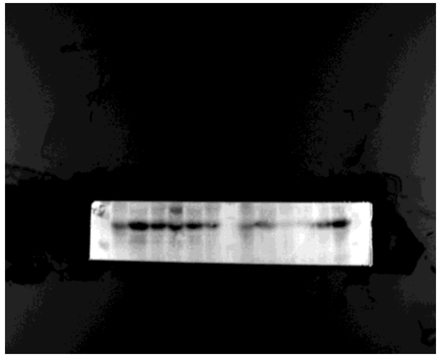

28day act

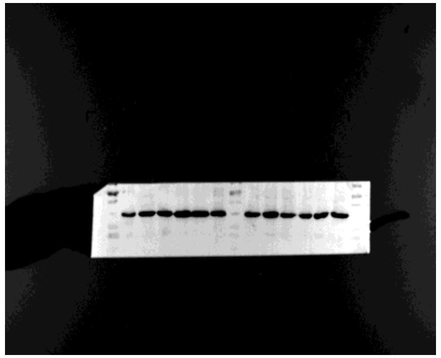

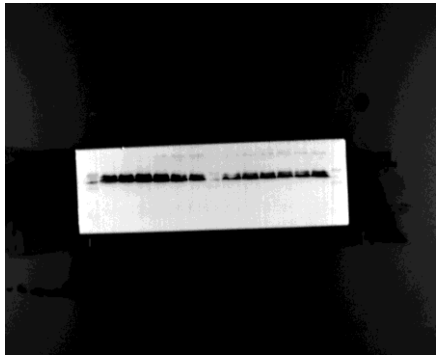

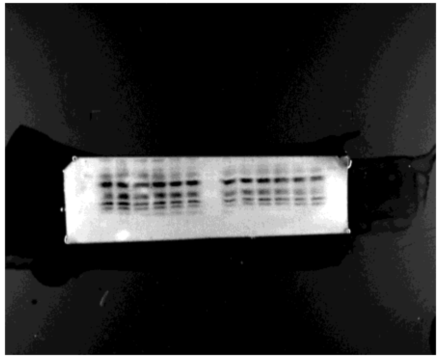

28day AT1

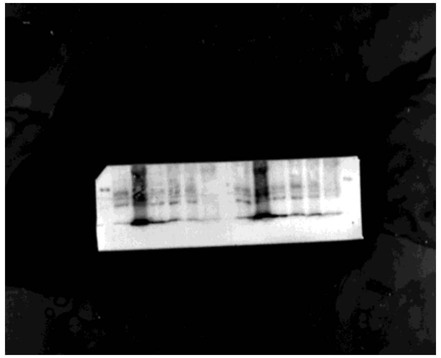

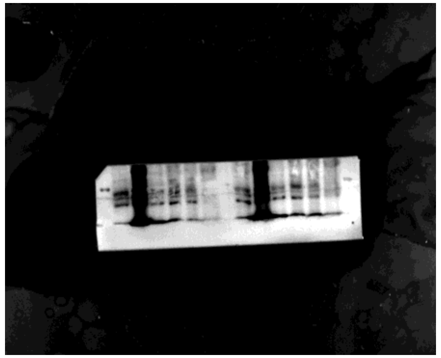

28day MMP2

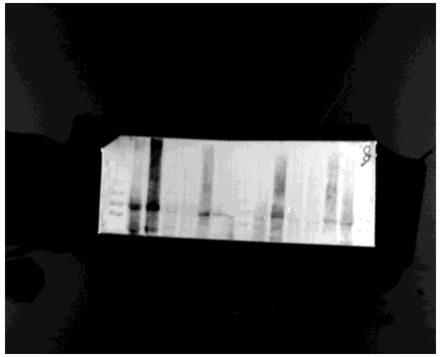

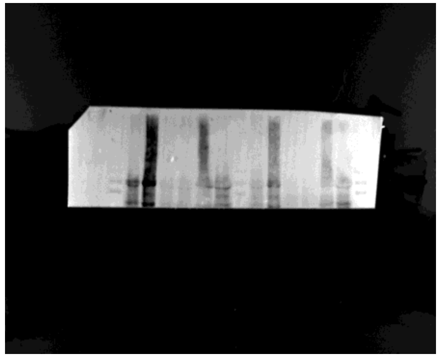

28day MMP9

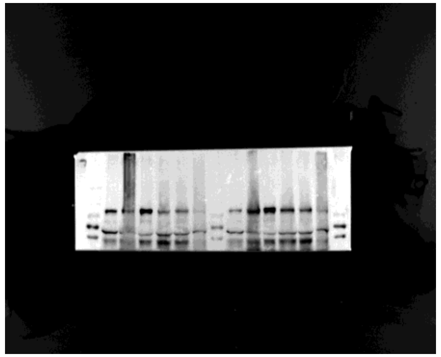

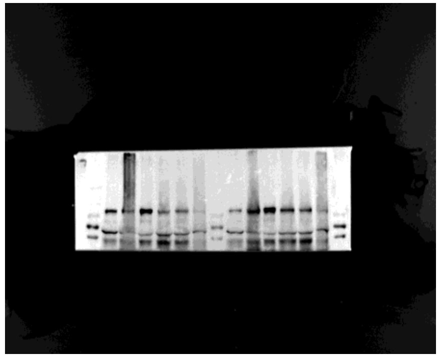

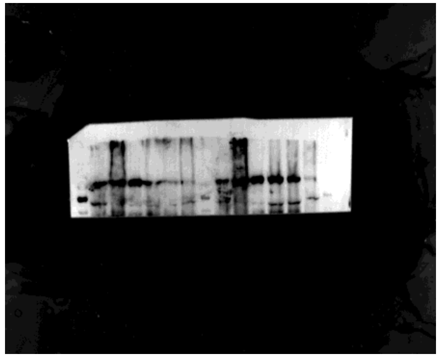

28day P-Smad2

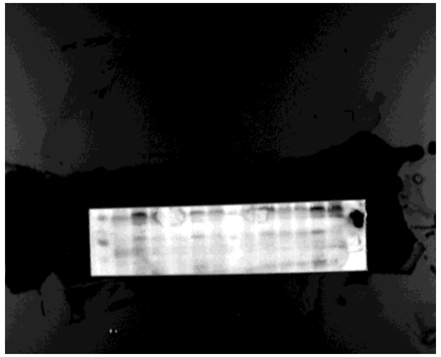

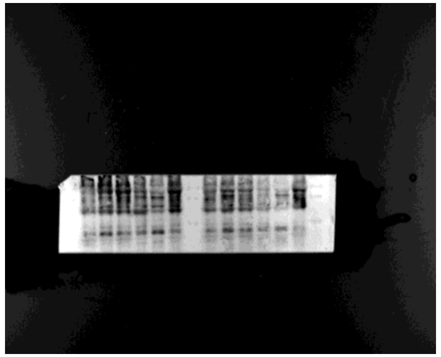

28day P-Smad3

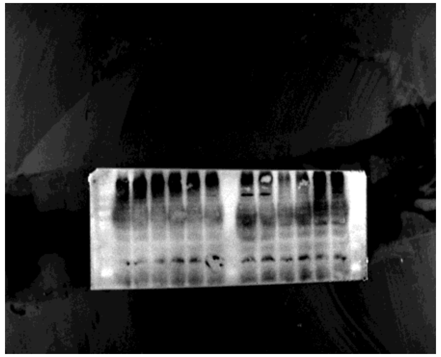

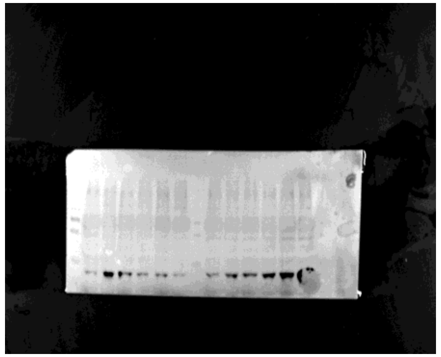

28day Smad2,3

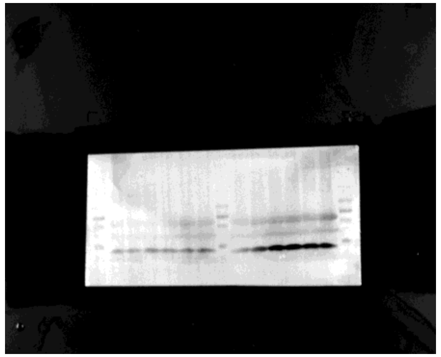

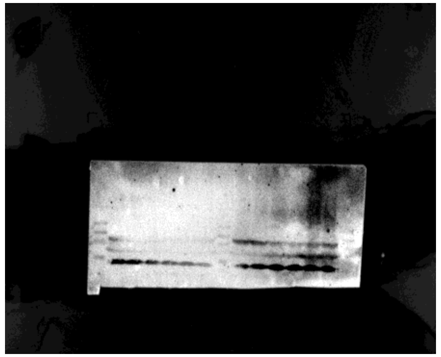

28day Snail

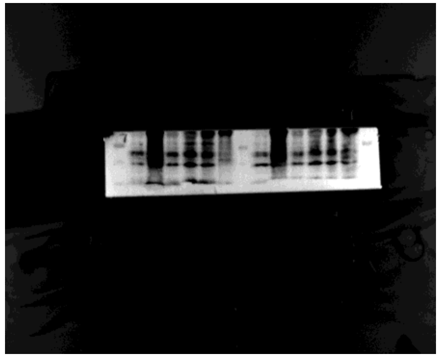

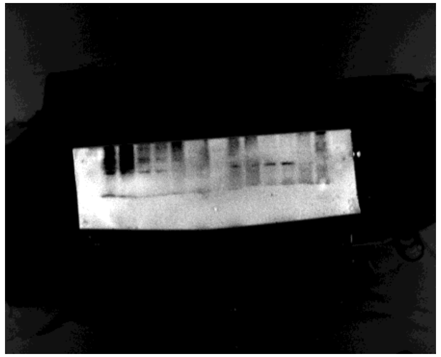

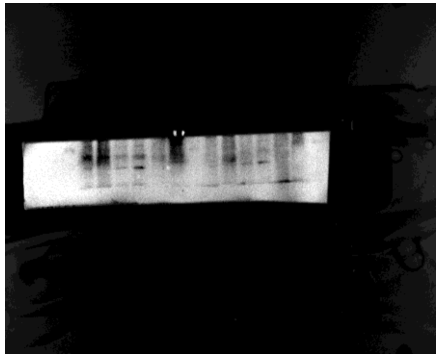

28day TGF-β1

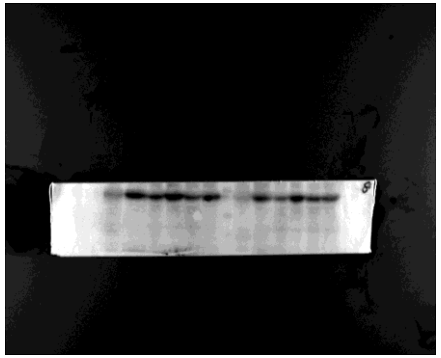

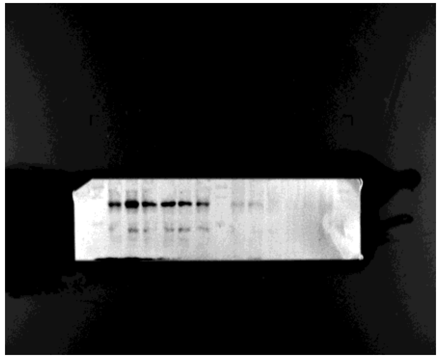

Supplement: Supplementary file 5 [file Data_Sheet_3.PDF]
