# Supplementary material for: Catalpol Attenuates Pulmonary Fibrosis by Inhibiting Ang II/AT1 and TGF-β/Smad-Mediated Epithelial Mesenchymal Transition
Source: Front Med (Lausanne). 2022 May 24;9:878601. doi: 10.3389/fmed.2022.878601 (PMC9171363; doi:10.3389/fmed.2022.878601)
Supplement: Supplementary file 6 [file Data_Sheet_4.DOCX]

**Table 1.** The relative expression of E-cadherin in lung tissue of mice in each group (x̅ ± SD)

|  | Control | BLM | BLM+L CAT | BLM+H CAT | BLM+PFD | BLM+TEL |
| --- | --- | --- | --- | --- | --- | --- |
| 7day | 9.46±3.63 | 5.54±2.54^###^ | 9.84±2.09*** | 9.03±1.46** | 9.01±1.60** | 9.49±3.34*** |
| 14day | 14.70±4.55 | 5.53±2.60^####^ | 13.92±4.35**** | 15.22±1.66**** | 12.35±4.19**** | 11.48±4.26*** |
| 28day | 15.73±4.04 | 4.46±1.88^####^ | 13.96±3.73**** | 8.71±3.82*** | 11.04±4.67**** | 12.48±3.55**** |

^###^*P* < 0.001, ^####^*P* < 0.0001 compared with the Control group, ***p* < 0.01, ****p* < 0.001, *****p* < 0.0001 compared with the BLM group (ANOVA with Dunnett’s post-hoc analysis).

**Table 2.** The relative expression of N-cadherin in lung tissue of mice in each group (x̅ ± SD)

|  | Control | BLM | BLM+L CAT | BLM+H CAT | BLM+PFD | BLM+TEL |
| --- | --- | --- | --- | --- | --- | --- |
| 7day | 4.50±2.33 | 9.76±2.41^####^ | 4.09±0.77**** | 4.08±1.42**** | 4.12±0.69**** | 3.81±0.69**** |
| 14day | 4.10±1.09 | 6.96 ±3.25^####^ | 5.03 ±1.04** | 3.79 ±0.59**** | 4.17 ±1.24**** | 3.85 ±1.34*** |
| 28day | 4.28±1.13 | 7.47±1.82^####^ | 4.47±1.09**** | 4.06±0.98**** | 2.69±0.71**** | 3.06±0.88**** |

^####^*P* < 0.0001 compared with the Control group, ***p* < 0.01, ****p* < 0.001, *****p* < 0.0001 compared with the BLM group (ANOVA with Dunnett’s post-hoc analysis).

**Table 3.** The relative expression of α-SMA in lung tissue of mice in each group (x̅ ± SD)

|  | Control | BLM | BLM+L CAT | BLM+H CAT | BLM+PFD | BLM+TEL |
| --- | --- | --- | --- | --- | --- | --- |
| 7day | 4.88±1.05 | 10.08±3.19^####^ | 5.84±1.89**** | 4.92±0.96**** | 4.97±1.58**** | 4.39±1.91**** |
| 14day | 4.18±0.93 | 9.62±2.91^####^ | 5.08±0.66 **** | 5.49±0.77**** | 4.33±0.31 **** | 4.88±2.71 **** |
| 28day | 4.29±0.86 | 7.79±1.09^####^ | 4.93± 0.85**** | 4.51±1.04**** | 4.52±1.18 **** | 4.47±0.63 **** |

^####^*P* < 0.0001 compared with the Control group, ****p* < 0.0001 compared with the BLM group (ANOVA with Dunnett’s post-hoc analysis).

**Table 4.** The content of Ang II in lung tissue of mice in each group (x̅ ± SD, ng/mL)

|  | Control | BLM | BLM+L CAT | BLM+H CAT | BLM+PFD | BLM+TEL |
| --- | --- | --- | --- | --- | --- | --- |
| 28day | 759.42±99.36 | 962.35±126.72^####^ | 744.07±39.59**** | 798.91±26.67*** | 776.38±46.02**** | 779.87±50.94**** |

^####^*P* < 0.0001 compared with the Control group, ****p* < 0.001, *****p* < 0.0001 compared with the BLM group (ANOVA with Dunnett’s post-hoc analysis).

**Table 5.** The content of HYP in lung tissue of mice in each group (x̅ ± SD, μg/mg)

|  | Control | BLM | BLM+L CAT | BLM+H CAT | BLM+PFD | BLM+TEL |
| --- | --- | --- | --- | --- | --- | --- |
| 7day | 0.37±0.05 | 0.51±0.11^#^ | 0.48±0.13 | 0.48±0.15 | 0.54±0.11 | 0.51±0.17 |
| 14day | 0.33±0.10 | 0.53±0.14^#^ | 0.37±0.11 | 0.38±0.05 | 0.34±0.12 | 0.38±0.14 |
| 28day | 0.38±0.13 | 0.69±0.20^##^ | 0.55±0.17 | 0.40±0.10** | 0.48±0.10 | 0.40±0.10*** |

^#^*P* < 0.05, ^##^*P* < 0.01 compared with the Control group, ***p* < 0.01, ****p* < 0.001 compared with the BLM group (ANOVA with Dunnett’s post-hoc analysis).

**Table 6.** The relative expression of AT_1_ in lung tissue of mice in each group (x̅ ± SD)

|  | Control | BLM | BLM+L CAT | BLM+H CAT | BLM+PFD | BLM+TEL |
| --- | --- | --- | --- | --- | --- | --- |
| 7day | 0.08±0.01 | 0.10±0.01 | 0.08±0.01 | 0.09±0.01 | 0.08±0.01 | 0.10±0.04 |
| 14day | 0.19±0.05 | 0.45±0.05^##^ | 0.26±0.07* | 0.23±0.06* | 0.22±0.07** | 0.18±0.12** |
| 28day | 0.06±0.01 | 0.16±0.01^####^ | 0.08±0.01**** | 0.07±0.02**** | 0.07±0.00**** | 0.08±0.02**** |

^##^*P* < 0.01, ^####^*P* < 0.0001 compared with the Control group, **p* < 0.05, ***p* < 0.01, *****p* < 0.0001 compared with the BLM group (ANOVA with Dunnett’s post-hoc analysis).

**Table 7.** The relative expression of MMP2 in lung tissue of mice in each group (x̅ ± SD)

|  | Control | BLM | BLM+L CAT | BLM+H CAT | BLM+PFD | BLM+TEL |
| --- | --- | --- | --- | --- | --- | --- |
| 7day | 0.10±0.02 | 0.10±0.03 | 0.09±0.01 | 0.09±0.01 | 0.08±0.02 | 0.08±0.01 |
| 14day | 0.07±0.04 | 0.47±0.09^####^ | 0.15±0.09*** | 0.18±0.02** | 0.22±0.03** | 0.22±0.10** |
| 28day | 0.04±0.02 | 0.15±0.04^###^ | 0.06±0.03** | 0.04±0.03*** | 0.05±0.01** | 0.05±0.01** |

^###^*P* < 0.001, ^####^*P* < 0.0001 compared with the Control group, ***p* < 0.01, ****p* < 0.001 compared with the BLM group (ANOVA with Dunnett’s post-hoc analysis).

**Table 8.** The relative expression of MMP9 in lung tissue of mice in each group (x̅ ± SD)

|  | Control | BLM | BLM+L CAT | BLM+H CAT | BLM+PFD | BLM+TEL |
| --- | --- | --- | --- | --- | --- | --- |
| 7day | 0.08±0.01 | 0.11±0.04 | 0.11±0.06 | 0.10±0.05 | 0.07±0.03 | 0.08±0.04 |
| 14day | 0.14±0.05 | 0.38±0.03^####^ | 0.26±0.06 | 0.27±0.03* | 0.26±0.03* | 0.29±0.05* |
| 28day | 0.06±0.01 | 0.14±0.04^##^ | 0.10±0.01 | 0.09±0.02 | 0.08±0.01* | 0.06±0.01** |

^##^*P* < 0.01, ^####^*P* < 0.0001 compared with the Control group, **p* < 0.05, ***p* < 0.01 compared with the BLM group (ANOVA with Dunnett’s post-hoc analysis).

**Table 9.** The relative expression of p-Smad2 in lung tissue of mice in each group (x̅ ± SD)

|  | Control | BLM | BLM+L CAT | BLM+H CAT | BLM+PFD | BLM+TEL |
| --- | --- | --- | --- | --- | --- | --- |
| 7day | 0.17±0.02 | 1.03±0.33^###^ | 0.31±0.25** | 0.30±0.14** | 0.33±0.17** | 0.16±0.02*** |
| 14day | 0.73±0.53 | 2.13±0.11^###^ | 1.28±0.25* | 1.17±0.16** | 1.09±0.18** | 1.36±0.27* |
| 28day | 0.11±0.01 | 0.33±0.06^####^ | 0.20±0.03** | 0.20±0.06** | 0.20±0.01** | 0.15±0.01*** |

^###^*P* < 0.001, ^####^*P* < 0.0001 compared with the Control group, **p* < 0.05, ***p* < 0.01, ****p* < 0.001 compared with the BLM group (ANOVA with Dunnett’s post-hoc analysis).

**Table 10.** The relative expression of Smad2/3 in lung tissue of mice in each group (x̅ ± SD)

|  | Control | BLM | BLM+L CAT | BLM+H CAT | BLM+PFD | BLM+TEL |
| --- | --- | --- | --- | --- | --- | --- |
| 7day | 0.76±0.04 | 0.65±0.08 | 0.76±0.12 | 0.77±0.13 | 0.77±0.12 | 0.67±0.03 |
| 14day | 0.24±0.07 | 0.24±0.06 | 0.26±0.06 | 0.35±0.11 | 0.33±0.14 | 0.35±0.19 |
| 28day | 0.69±0.11 | 0.70±0.07 | 0.70±0.08 | 0.72±0.13 | 0.70±0.10 | 0.70±0.10 |

(ANOVA with Dunnett’s post-hoc analysis).

**Table 11.** The relative expression of Snail in lung tissue of mice in each group (x̅ ± SD)

|  | Control | BLM | BLM+L CAT | BLM+H CAT | BLM+PFD | BLM+TEL |
| --- | --- | --- | --- | --- | --- | --- |
| 7day | 0.08±0.01 | 0.15±0.00^##^ | 0.11±0.03 | 0.11±0.03* | 0.09±0.01* | 0.09±0.01* |
| 14day | 0.10±0.08 | 0.49±0.02^####^ | 0.15±0.04*** | 0.13±0.06*** | 0.14±0.10*** | 0.12±0.08*** |
| 28day | 0.05±0.00 | 0.13±0.02^###^ | 0.08±0.01* | 0.06±0.01*** | 0.07±0.01** | 0.05±0.03*** |

^##^*P* < 0.01, ^###^*P* < 0.001, ^####^*P* < 0.0001 compared with the Control group, **p* < 0.05, ***p* < 0.01, ****p* < 0.001 compared with the BLM group (ANOVA with Dunnett’s post-hoc analysis)

**Table 12.** The relative expression of TGF-β1 in lung tissue of mice in each group (x̅ ± SD)

|  | Control | BLM | BLM+L CAT | BLM+H CAT | BLM+PFD | BLM+TEL |
| --- | --- | --- | --- | --- | --- | --- |
| 7day | 0.43±0.04 | 0.99±0.14^##^ | 0.70±0.08 | 0.57±0.16* | 0.06±0.25* | 0.47±0.12** |
| 14day | 1.01±0.34 | 1.83±0.48^##^ | 1.44±0.40 | 1.48±0.35 | 1.24±0.15 | 1.60±0.71 |
| 28day | 0.31±0.07 | 0.97±0.07^####^ | 0.58±0.17*** | 0.66±0.04** | 0.53±0.02*** | 0.55±0.06*** |

^##^*P* < 0.01, ^####^*P* < 0.0001 compared with the Control group, **p* < 0.05, ***p* < 0.01, ****p* < 0.001 compared with the BLM group (ANOVA with Dunnett’s post-hoc analysis)

**Table 13.** The relative expression of p-Smad3 in lung tissue of mice in each group (x̅ ± SD)

|  | Control | BLM | BLM+L CAT | BLM+H CAT | BLM+PFD | BLM+TEL |
| --- | --- | --- | --- | --- | --- | --- |
| 7day | 0.19±0.05 | 0.61±0.06^####^ | 0.16±0.04**** | 0.27±0.04**** | 0.11±0.00**** | 0.10±0.03**** |
| 14day | 0.28±0.05 | 0.43±0.08 | 0.32±0.09 | 0.30±0.05 | 0.33±0.08 | 0.32±0.09 |
| 28day | 0.14±0.04 | 0.41±0.07^##^ | 0.19±0.07** | 0.20±0.10* | 0.20±0.07** | 0.16±0.13** |

^##^*P* < 0.01, ^####^*P* < 0.0001 compared with the Control group, ***p* < 0.01, *****p* < 0.0001 compared with the BLM group (ANOVA with Dunnett’s post-hoc analysis)
